# Highlighting membrane protein structure and function: A celebration of the Protein Data Bank

**DOI:** 10.1016/j.jbc.2021.100557

**Published:** 2021-03-18

**Authors:** Fei Li, Pascal F. Egea, Alex J. Vecchio, Ignacio Asial, Meghna Gupta, Joana Paulino, Ruchika Bajaj, Miles Sasha Dickinson, Shelagh Ferguson-Miller, Brian C. Monk, Robert M. Stroud

**Affiliations:** 1 Department of Biochemistry and Biophysics, University of California San Francisco, San Francisco, California, USA; 2 Department of Neurology, University of California San Francisco, San Francisco, California, USA; 3 Department of Biological Chemistry, School of Medicine, University of California Los Angeles, Los Angeles, California, USA; 4 Department of Biochemistry, University of Nebraska–Lincoln, Lincoln, Nebraska, USA; 5 DotBio Pte. Ltd, Singapore, Singapore; 6 Department of Bioengineering and Therapeutic Sciences, University of California San Francisco, San Francisco, California, USA; 7 Department of Biochemistry and Molecular Biology, Michigan State University, East Lansing, Michigan, USA; 8 Sir John Walsh Research Institute and Department of Oral Sciences, University of Otago, North Dunedin, Dunedin, New Zealand

**Keywords:** membrane protein, structure-function, lipid mimetics, bioenergetics, membrane protein biogenesis, transmembrane transport, channel, receptor, drug discovery, protein design, -NPA-, -Asparagine-Proline-Alanine, 2D, Two-Dimensional, 3D, Three-Dimensional, ACE2, Angiotensin Converting Enzyme 2, AmT, Ammonia Transport, AQP, Aquaporin, aR, Aromatic Arginine, BAM, *β* -Barrel Assembly Machinery, cCpE, C-terminal Domain of CpE, CF, Cystic Fibrosis, CFTR, Cystic Fibrosis Transmembrane-Conductance Regulator, CHIP28, Channel-like Integral membrane Protein of 28 kDa, ChR, Channel Rhodopsin, CLDN, Claudin, COVID-19, Coronavirus Disease 2019, CpE, *Clostridium perfringens* Enterotoxin, cpEGFP, Circularly Permutated Enhanced Green Fluorescent Protein, CPR, NADPH-Cytochrome P450 Reductase, DA, Dopamine, DAT, Dopamine Transporter, dLight, Dopamine Fluorescent Sensor, ECS, Extracellular Segment, EMC, Endoplasmic Reticulum Membrane Complex, ENC, Extracellular Negative Clusters, ER, Endoplasmic Reticulum, GABA R, Gamma-Amino Butyric Acid Receptor, GET, Guided-entry of Tail-Anchored, GFP, Green Fluorescent Protein, GLUT, Glucose Transporter, GPCR, G-Protein-Coupled Receptor, HCS, Hydrophobic Constriction Site, IA, Influenza A, IBD, Inflammatory Bowel Disease, ICH, Intracellular Helix, iGluSnFR, Intensity-Based Glutamate-Sensing Fluorescent Reporter, INC, Intracellular Negative Clusters, KcsA, K^+^-Channel of *Streptomyces lividans* A, K_v_, Voltage-Gated K^+^-channel, LacY, Lactose Permease, LCP, Lipidic Cubic Phase, LeuT, Leucine Transporter, LGIC, Ligand-Gated Ion Channel, MAOIs, Monoamine Oxidase Inhibitors, MBP, Maltose-Binding Protein, MD, Molecular Dynamics, MFS, Major Facilitator Superfamily, MP, Membrane Protein, Na_v_, Voltage-Gated Na^+^-channel, NavAb, Voltage-Gated Na^+^-Channel from *Arcobacter butzleri*, NBD, Nucleotide-Binding Domain, NET, Noradrenaline Transporter, NSS, Neurotransmitter Sodium Symporter, PDB, Protein Data Bank, *Pf*, *Plasmodium falciparum*, PTEX, Plasmodium Translocon of Exported proteins, Rh, Rhesus Factor, RNC, Ribosome-Nascent Chain, SARS-Cov, Severe Acute Respiratory Syndrome Coronavirus, SERT, Serotonin Transporter, SF, Selectivity Filter, SLC, Solute Carrier, SR, SRP receptor, SRP, Signal Recognition Particle, SSRI, Selective Serotonin Reuptake Inhibitors, TA, Tail-Anchored, TC, Targeting Complex, TCA, Tricyclic Antidepressant, TJ, Tight Junction, TM, Trans-Membrane *α*-helix, TMD, Trans-Membrane Domain, TPC3, Two-Pore channel 3, TRP, Transient Receptor Potential, VGIC, Voltage-Gated Ion Channel, VSD, Voltage-Sensing Domains, VP, Viroporin

## Abstract

Biological membranes define the boundaries of cells and compartmentalize the chemical and physical processes required for life. Many biological processes are carried out by proteins embedded in or associated with such membranes. Determination of membrane protein (MP) structures at atomic or near-atomic resolution plays a vital role in elucidating their structural and functional impact in biology. This endeavor has determined 1198 unique MP structures as of early 2021. The value of these structures is expanded greatly by deposition of their three-dimensional (3D) coordinates into the Protein Data Bank (PDB) after the first atomic MP structure was elucidated in 1985. Since then, free access to MP structures facilitates broader and deeper understanding of MPs, which provides crucial new insights into their biological functions. Here we highlight the structural and functional biology of representative MPs and landmarks in the evolution of new technologies, with insights into key developments influenced by the PDB in magnifying their impact.

As membranes were critical to the separation of chemistries essential to the origin of life, membrane proteins (MPs) are key players in some of the most important physiological processes in living organisms. Characterizing MPs structurally and functionally is still extremely challenging due to their frequent low abundance, and difficulties in purifying functional MPs intact from their native membrane, though it is going through an exciting revolution now due to several key factors. It took several decades to obtain the structural information that now allows pursuit of understanding MP function in health and disease, to manipulate them as drug targets, and to engineer them into new powerful tools to fuel discovery. We highlight some of the landmarks in this endeavor that drove or depended on the discovery of new technologies required specifically for structural studies of membrane, *versus* soluble proteins.

Previously, hand-written letters delivered by post requested coordinate sets that were not always readily given. The vision of Hamilton, Myer, Koetzle, and the joint venture between the Cambridge Crystallographic Data Center in the United Kingdom and the Brookhaven National Laboratories at Stony Brook University led to the Protein Data Bank (PDB). Then for the first time, one could begin to ask questions with all the relevant structures at hand. In celebrating 50 years of the PDB, we focus on some of the ingeniously crafted inventions and discoveries that led the way for entire classes of MPs, and those new approaches that now promise structures of large and complex machines from their native cellular environments, in action.

In the following Historical perspective, we provide a brief historical perspective of some MP insights (other than the crucial G-protein-coupled receptors (GPCRs) that are the subject of a dedicated review in this volume) and consider the value of the PDB in disseminating this information. Seemingly insurmountable difficulties were often overcome with invention of new technologies to reveal important structural features of classes of MPs that make the fabric of today's approaches.

In How do membrane proteins accomplish key physiological functions?, we describe how the structures of several of the major MP classes were uncovered, which often required technological developments that are now woven into the fabric of structural biology. First, how are the *α*-helical, tail-anchored, and all *β*-sheet MP broad categories correctly targeted to and inserted into membranes and allowed to fold correctly? We progress to landmark discoveries involving the roles of MPs in physiology and some of the critical barriers that had to be overcome to realize these achievements. How do water channels conduct water at diffusion limited rates without allowing leakage of protons (H^+^) or hydronium ions (H_3_O^+^) or any other ions? How do potassium channels conduct K^+^ ions, but not the Na^+^ ion of similar charge and smaller ionic radius? How are epithelial cells held together side by side to make a selectively permeable sealed layer of cells? The question of how substrates are transported across membranes using energy then follows. How does one superfamily of “primary” transporters, the “ABC transporters” that directly harness ATP hydrolysis on their cytoplasmic side, move materials across membranes? How does another superfamily of “secondary” transporters, the Major Facilitator Superfamily (MFS), use ion or proton electrochemical gradients to drive nutrient import, export, or efflux of xenobiotics? We progress to consider a specialized class of essential viral proteins that form channels that are essential for viral virulence.

In Membrane protein structures instruct drug design and protein engineering, we focus on a few examples of therapeutics and opportunities for engineering of MPs. Critical to any therapeutic drugs are the membrane-attached cytochrome P450s that are discussed in the light of their key roles in sterol metabolism, but as metabolizers of therapeutic drugs, and therefore as drug targets themselves. Structures help elucidate mechanisms of action of therapeutics to modulate MP activities. The Cystic Fibrosis Transmembrane-Conductance Regulator (CFTR) is used as an example of the impact structural studies can have on the understanding and treatment of rare diseases. Another example addresses glucose import as an anticancer target opportunity. A key example in neurology is the class of ligand-gated ion channels, represented by GABA_A_ receptors (GABA_A_R) and their ligands. Then the Leucine Transporter (LeuT), representing several classes of transporters with a common core of ten trans-membrane *α*-helices (TMs), is used to illustrate how major antidepressants work. Finally, some exciting developments in MP engineering focus on channelrhodopsins where light can trigger cellular responses and engineered dopamine sensors.

We hope that this necessarily limited perspective on the impact of selected MP structure classes may encourage opportunities in a broader context. We hope to communicate the value of MPs as guardians of the health of cells and how their structures, through the PDB, contribute important insights into many crucial aspects of physiology.

## Historical perspective

### A brief history of membrane protein structures and the PDB

**Figure 1:**
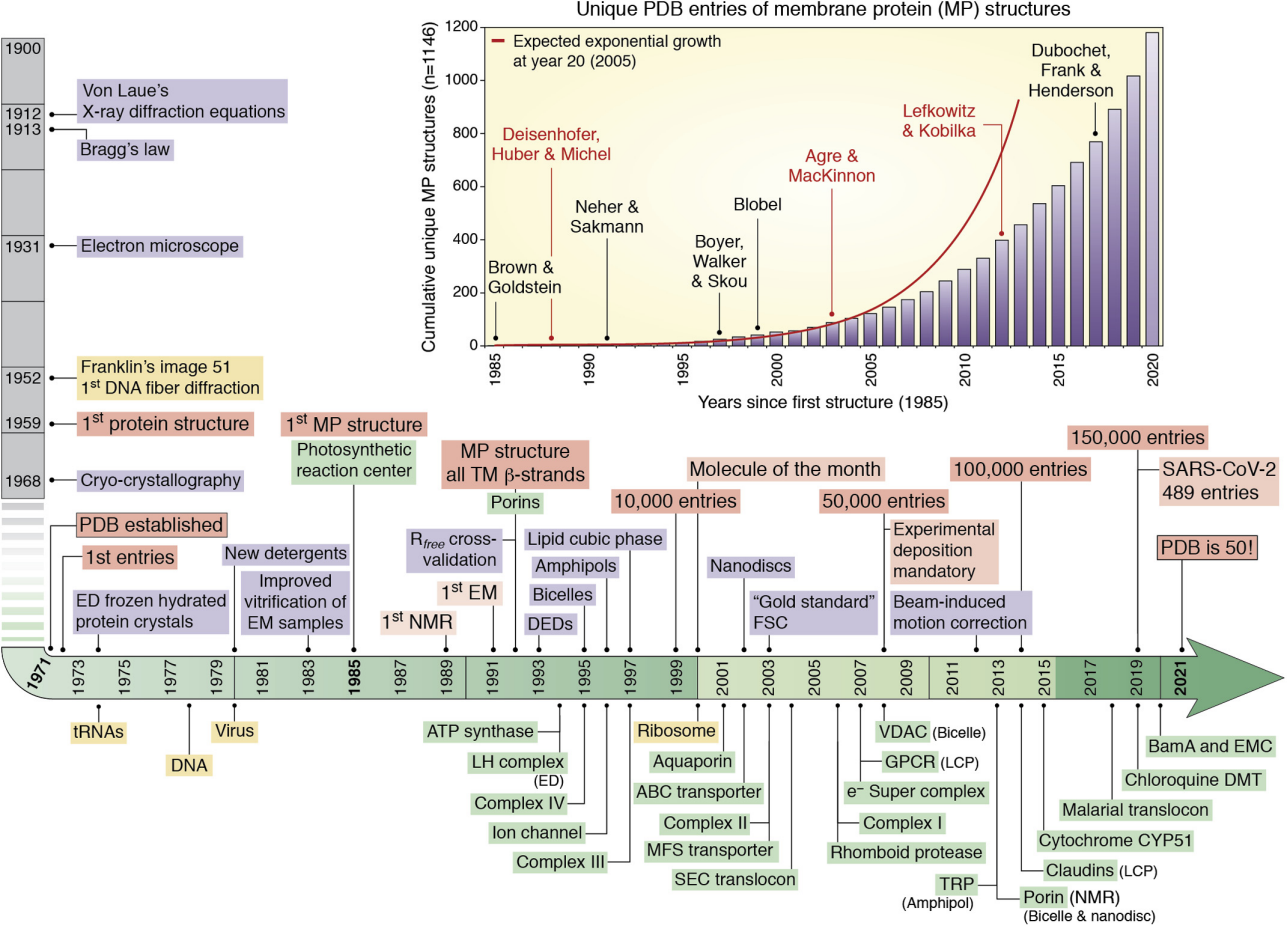
**Timeline depicting the progress in structural biology since the PDB was established in 1971 and evolution of membrane protein structural biology since 1985.** The curve showing the cumulative total of *unique* MP structures deposited in the PDB (mostly by X-ray crystallography and cryo-EM) as a function of time is adapted from the *mpstruct* website of Stephen White at the University of California, Irvine at https://blanco.biomol.uci.edu/mpstruc/. The *red curve* models the expected exponential growth at year 20 (2005). Dates corresponding to key scientific or technical discoveries that fostered breakthroughs in deciphering and understanding (membrane) protein structure and function are indicated. Some of the first protein structures reported for important classes are indicated along the timeline. Many of the first MP structures were solved in detergent but later in lipidic cubic phase bilayers LCP, bicelles, amphipols, and nanodiscs. ED: electron diffraction. DED: direct electron-counting detector. R_*free*_: test set cross-validation for crystallographic refinement. FSC: Fourier shell correlation for cryo-EM single-particle reconstruction validation. LH: light harvesting complex. Chloroquine DMT: chloroquine-resistance drug/metabolite transporter. This list does not pretend to be exhaustive. The *Protein Structure Initiative* from the National Institute of Health in the United States spanned the years 2000 to 2015 (shaded in *light green*). Nobel prizes awarded for discoveries related to MP biology and structure are indicated. The central motivation behind the “Molecule of the Month” (35 MPs since its creation) is to provide a user-friendly introduction to a rich body of data, providing users with a path to get started with finding and exploring the many available structures and thus offering a resource for molecular biology educators and students around the world at http://pdb101.rcsb.org (81).

With soluble proteins beginning with myoglobin in 1961, followed by lysozyme, hemoglobin, and digestive proteases, these were available only from large animals or from bacteria. Long before overexpression systems became available, the number of soluble macromolecular structures followed an exponential growth. This pattern was noted by Dickerson in a letter written to the PDB in 1978 (1). The first integral MP structure was not determined until 25 years later in 1985. The number of MP structures determined and deposited in the PDB since that time also increased exponentially, but with a smaller exponent reflecting the considerable challenges that pertain to MP structural biology. The amount of time for the number of unique MP structures to double was about 3 years compared with 2.4 years for soluble macromolecules (2). This doubling time for MPs slowed to ∼5 years recently (Fig. 1). Most obvious challenges are functional expression in a limited lipid membrane environment *versus* expression in a soluble volume, followed by the requirement for detergents, amphiphiles, and lipids during extraction and purification of functional proteins from the membrane. There were critical breakthroughs necessary for many new classes of MPs over the last three decades. As a reflection, on January 15th_,_ 2021, there were a total of 4569 coordinate files for MPs in the PDB, barely 2.6% of the total for all proteins, and of these 1203 represent unique structures. This is brilliantly tracked and annotated by Stephen H. White’s invaluable *mpstruct* database of MPs of known structure (Fig. 1) (2) (https://blanco.biomol.uci.edu/mpstruc/), and by statistical and thermodynamic analyses from Thomas Newport, Mark Sansom, and Phillip Stansfield in their *MemProtMD* database (3). One of our aims here is to celebrate some of these developments in association with the breakthroughs by those that made them possible.

Like those in the 1960s who worked on the soluble proteins available in quantity from the tissues of large animals or bacteria, work on the first MP structures also focused on rich natural sources. The purple membrane of archaebacteria was first described in 1971 by Oesterhelt and Stoeckenius, who showed that it contained a light-driven proton pump. This validated the Mitchell hypothesis, the revolutionary concept that biological energy could be stored as a proton gradient across a membrane (4, 5). By freeze-fracture electron microscopy, they showed that “bacteriorhodopsin” formed an in-plane trigonal two-dimensional (2D) lattice. This was pursued structurally by Henderson and Unwin using electron diffraction of the arrays formed in the membrane (6), by Michel working with Oesterhelt, who attempted to crystallize the protein using detergents (7, 8, 9, 10), and by Blaurock using X-ray scattering to demonstrate that *α*-helices were oriented perpendicular to the membrane plane (11, 12). Henderson and Unwin produced the first electron diffraction patterns from the trigonal latticed membranes, purified from the natural source, by sustaining its single bilayer in a glucose solution to prevent dehydration in the electron microscope (6). In a towering landmark, in 1975, they phased the patterns based on the images and reconstructed a 7 Å resolution 3D structure that beautifully demonstrated that the protein is comprised of seven TMs (6). This first discovery using electron microscopy/diffraction was at the heart of the concepts later recognized by the Nobel prize in chemistry to Henderson, Frank, and Dubochet in 2017. Soon after, in 1977 to 1979, the amino acid sequence of bacteriorhodopsin was determined by the groups of Khorana (13) and Ovchinikov (14, 15) using chemical sequencing methods. Agard and Stroud developed computational ways to extend resolution perpendicular to the membrane plane in attempts to reveal the interhelix linker regions to map sequence into the structure from electron diffraction data (16). With Glaeser, who pioneered low-temperature electron microscopy (17, 18), Hayward and Stroud developed a way of aligning microcrystalline regions to extend resolution of the reconstructions to 3.8 Å in-plane resolution (19). As a reflection of the extreme challenges for membrane *versus* soluble proteins, the bacteriorhodopsin structure reached atomic resolution by electron diffraction 20 years after the 1975 breakthrough in 1996 (20, 21) and by X-ray diffraction in 1997 (22) (Fig. 1).

The cytochrome oxidase from bovine tissues (23, 24) and the acetylcholine receptor from electric rays and eels (25, 26, 27, 28, 29), respectively, key to oxidative phosphorylation in mitochondria (see Early breakthroughs in High Resolution Structures of Bioenergetic Membrane Complexes and Lipid Interactions) and function of the neuromuscular junction, also provided highly important sources of MP complexes. Beginning in 1971, the first 3D surface shapes of the acetylcholine receptor ∼300 kDa complex of five homologous subunits in a quasi-fivefold pentameric complex *αβαγδ* were revealed using low-dose negative stain EM and small-angle X-ray scattering from stacked membranes by Stroud and Unwin (30, 31, 32, 33, 34, 35). The primary sequence, determined initially by Michael Raftery using amino acid sequencing for 50 amino acids of each of the four homologous subunits (28, 36), and in parallel by Jean-Pierre Changeux for the *α*-chain N-terminal sequence (27, 29), led to the cloning and sequencing of the genes (37). It became clear that like bacteriorhodopsin, it too contained TMs. The right-handed *α*-helix first delineated by Pauling (38) provided the necessary shielding of all polar groups, namely the carbonyls and amides of the polypeptide backbone. Hence, a sequence of ∼19 hydrophobic amino acids became a recognizable signal for TMs, while strongly amphipathic helices signal membrane surface-seeking helices (39). Remarkable insights followed from the few MP structures already determined, including the finding that their hydrophobic helices formed well-ordered 3D structures in lipid bilayer membranes and their retention even after gentle extraction in amphiphiles and detergents. Yet it took 45 years to obtain the first atomic structures for the acetylcholine receptor. This required screening of many specific genetic constructs of several members of this receptor family (40, 41).

The first atomic resolution structure of any integral MP came from the group of Michel in 1985 (42). After many years trying to crystallize bacteriorhodopsin, Michel and Oesterhelt had appreciated the value of colored proteins such as bacteriorhodopsin. Visible bands on columns aided purification, and more importantly, where the color of unique spectral features reflected the integrity of the MP (43) and signaled for monitoring structural preservation in harsh solubilizing detergents. Using photobacteria as a rich source of photosynthetic proteins, Michel and colleagues determined the first MP structure from crystals that diffracted to atomic resolution (3 Å and then at 2.3 Å). The structure of the *Rhodopseudomonas viridis* photosynthetic reaction center of 145 kDa published in 1984 (42) remains an amazing achievement. It showed that the predominantly hydrophobic *α*-helices are often longer than enough to span the 40 Å bilayer and can be correspondingly tilted at different angles to harbor the many chromophores that harness the light that supports life. This breakthrough discovery was recognized by the Nobel prize in chemistry in 1988 to Michel, Deisenhofer, and Huber (Fig. 1).

Ion channels in membranes were in the limelight because they accounted for the currents across membranes that are key to the nervous system, as described by Hodgkin, Huxley, and Katz in 1952 (44, 45, 46), and beautifully elaborated since then. The key was that Na^+^ ions are consistently pumped out of the mammalian cell using energy from ATP hydrolysis, while potassium ions remain inside to balance the electrochemical potential across the plasma membranes according to the Nernst equation. In neurons, however, the need for fast communication relies on rapid conduction of the Na^+^ ions inward that is signaled by the release of neurotransmitters at neuronal synapses that depolarize the plasma membranes of the target cell. This is then rapidly followed by release of K^+^ ions though highly selective channels that do not leak the smaller Na^+^ ions, but restore the transmembrane electrical potential. How is such selectivity accomplished? The alkali metal Na^+^ and K^+^ ions differ in ionic radius, yet why don’t K^+^ channels leak the smaller Na^+^ ion? The answer came in MacKinnon’s finding that bacterial K^+^ channels existed and could be extracted for structure determination. In 1998, he reported the first K^+^ channel structure (KcsA) from the bacterium *Streptomyces lividans* (47). The structure showed precisely how its selectivity was achieved. The key was in the precise arrangement of a fourfold arrangement of lines of carbonyl oxygens that surround a “selectivity filter” (SF) in the pore entry. They provide the precise counterpart for the normal water hydration shell around the K^+^ ion, but were too far apart to provide similar energy balance for the Na^+^ ion. MacKinnon was awarded the Nobel Prize in chemistry for this landmark discovery in 2003 (Fig. 1).

The 2003 Nobel prize in chemistry was shared with Agre, recognizing another remarkable discovery, that of water channels (48). In 1985, Benga *et al.* (49, 50) demonstrated that red blood cells had water channels that were inhibitable by mercurials, implying that a sulfhydryl containing protein was responsible. They showed that this was true across many (now all) species, and Nielsen and Agre (51) showed that water channels presented in all cells but for various physiological purposes. They showed that there were 13 different variants in human tissues and named them Aquaporins (AQPs) (52). These were more difficult to recognize than ion channels because there were no electrical properties to measure their activity. Their discovery was made while searching for the well-known rhesus (Rh) factors in red blood cells. They discovered a second major MP in these cells, and then showed that it conducted water, and at a speed up to the diffusion-limited values for a pore of the requisite size (see below). Structures of the aquaporins were determined in 2000 by electron imaging (53) and at atomic resolution by X-ray crystallography (54), showing precisely how these channels excluded passage of protons (H^+^) or hydronium ions (H_3_O^+^), while allowing a single file of water molecules to progress, hydrogen bonded to themselves, and to eight key carbonyl oxygen atoms that act like pitons along the walls of the channel (55).

An outstanding landmark was the structure of the F_1_F_o_-ATPase by Walker in 1994 (56) and its dynamo-like rotation that harnessed proton gradients to drive the synthesis of ATP in mitochondria. These critical structures of a ∼500 kDa complex provided the amazing link to elucidate the subunit stoichiometry of eight different protein chain types needed to harness proton gradients to produce ATP, the major source of energy in all cells. The discoveries led to the Nobel Prizes in chemistry in 1997 to Boyer and to Walker, who reported the atomic structure of the complex in 1994 (56) (Fig. 1).

The LeuT structure determined by Gouaux's group signaled a new approach to several related transporters that are critical to mental health, responses to neurotransmitters, to therapeutic and street drugs (57, 58, 59, 60). This first structure provided a landmark insight into the transporters that are required to repackaged and reabsorb neurotransmitters back into the neuronal cell after release into neurological synapses. Dopamine, serotonin transporters, and others from the same family are all targets of therapies for the critically important spectrum of neurological disorders (61). These transporters are also key to the control of major mental diseases including such poorly understood disorders as schizophrenia and bipolar disease that together affect ∼1/50 persons globally. The PDB now provides a critical catalyst for disciplines that need to “know what we are dealing with” in human health *versus* disease. It creates a scenario in which we can think about and design strategies for modulating therapeutic responses as well as providing an ongoing reminder of how evolution has separated chemistries in order to provide the essence of life.

With increasing impact on prospects for drug discovery, structures of membrane embedded proteases that cleave transmembrane regions to release components are critical. One of the first key examples was discovered by Brown, Goldstein, and colleagues in the critical regulation of cholesterol metabolism. In recognition of their many contributions to the broader field of cholesterol metabolism, they were awarded the Nobel prize in Physiology and Medicine in 1985. They subsequently showed that a membrane-embedded S1P serine protease cleaves the sterol regulatory element-binding protein SREBP. A second intramembrane protease S2P, a zinc metalloprotease, liberates a transcription factor from the N terminus of SREBP that then upregulates cholesterol synthesis. These proteases also coordinate fatty acid synthesis that is also required for membrane biogenesis (62, 63, 64). Rhomboid proteases constitute another superfamily of ubiquitous intramembrane proteases involved in a wide range of biological processes and were the first structures of intramembrane proteases to be determined (65). Another critical example of intramembrane proteolysis is the cleavage of the *β*-amyloid precursor protein (APP) by the *β*-site cleaving enzyme (BACE1) that converts APP to the pathogenic forms A*β*42/40. Validated by mouse gene knockouts, BACE1 and related *γ*-secretase (66, 67, 68) are thus targets for drugs (69, 70). In spite of extremely difficult clinical trials, these targets remain as promising modulators of Alzheimer's disease (71). Many of the intramembrane proteases destabilize the *α*-helical regions that characterize transmembrane proteins and often don’t rely on specific amino acid side chains for specificity as do the soluble proteases.

As a portent of the impact of electron microscopy, the discovery of the TRPV1 channel structure came after several groups had purified and tried extensively to crystallize Transient Receptor Potential (TRP) channels for many years in seeking the structural basis for their roles in pain sensing (72). As cryo-EM technologies improved, driven by single electron counting “direct detectors” for cryo-EM (73), TRPV1 finally became tractable. This breakthrough by Cheng working with Julius heralded the maturation of cryo-EM applied to MPs (72, 74). It was harnessed by structural biologists particularly with integral MPs where the challenges of crystallography and pressure for discovery were already high (75, 76, 77, 78, 79, 80) (Fig. 1).

As a critical library of the molecular bases for MP function, the PDB provides a historical timestamp of the progression in our understanding of these important proteins. It uniquely affords a quantitative visual imprint of the mechanisms of these proteins as the gatekeepers, importers, and exporters of the cell and of organelles within the cell, to the benefit of current and future generations.

### Early breakthroughs in high-resolution structures of bioenergetic membrane complexes and lipid interactions

The story of MP structure determination and its profound impact on understanding biological systems is a fascinating illustration of the power of technology development and the value of rapid communication of detailed structural information through the PDB. The problem of transferring MPs from lipid environments to an aqueous phase is substantial and was perhaps initially underestimated due to the prevalent concept that the detergents needed to accomplish this feat were intrinsically disordered and simply required to create a not-necessarily-homogeneous hydrophobic phase to cover newly exposed membrane-embedded regions. Indeed, the importance of pure, well-defined detergents was not immediately recognized, and when it was, the chemistry for creating and purifying these amphipathic compounds was challenging. But gradually it became clear that achieving high-resolution MP crystal structures required a protein sample that had not only native activity and a high degree of purity but also was dependent on the specific structure and purity of the detergent itself for achieving these ends. In many cases, success also depended on retaining a subset of structurally important lipids (82).

**Figure 2:**
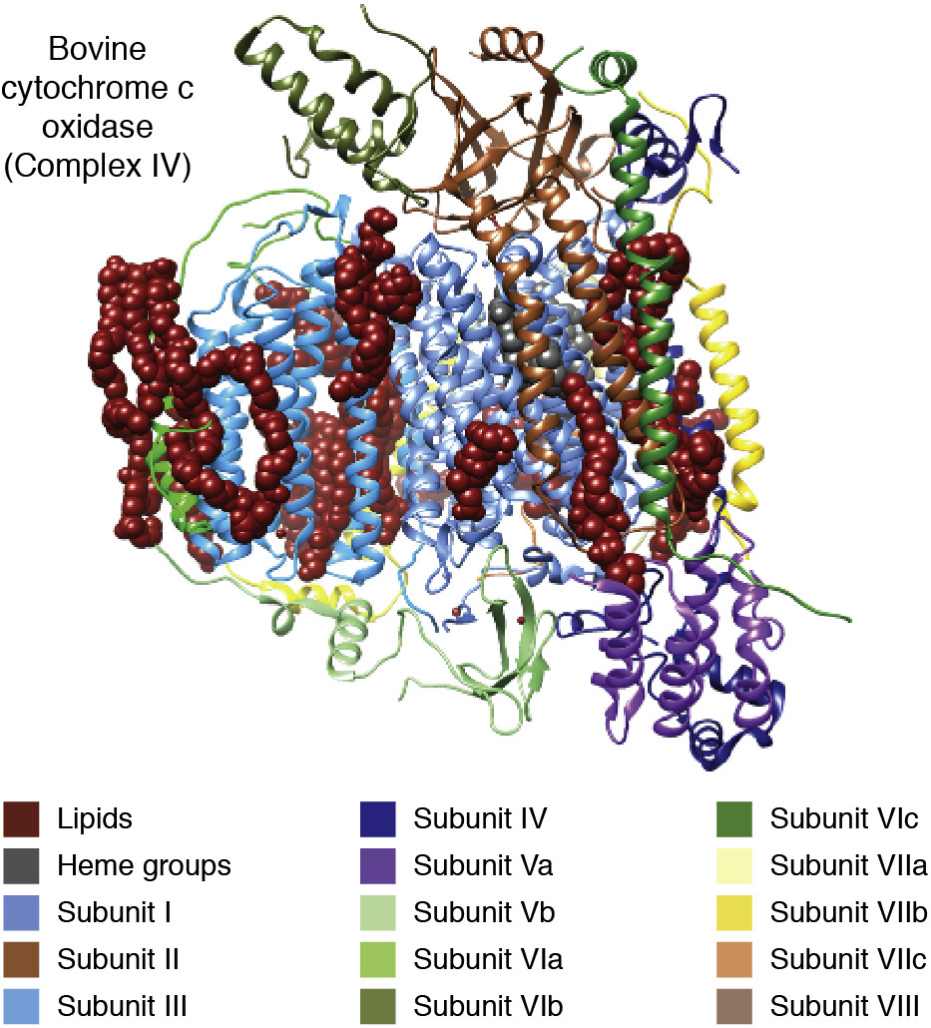
**Bovine cytochrome *c* oxidase (Complex IV) monomer with specifically bound lipid.** The 13-subunit monomer structure (PDB 2EIL chains N–Z) is shown, with associated lipids in *dark red*, buried hemes in *dark gray*, and membrane region delineated by lipid placement. Subunits: I, *cornflower blue*; II, *sienna*; III, *blue*; IV, *navy*; Va, *purple*; Vb, *light green*; VIa, *green*, VIb, *olive*, VIc, *forest green*; VIIa, *yellow*; VIIb, *gold*; VIIc, *orange*; VIII, *khaki*.

Following the advent of the earliest high-resolution MP structure (42), the importance of specific lipid interactions became increasingly apparent: the lipidic detergent molecules as well as native lipids were not just satisfying the need for a disordered hydrophobic phase, but many were actually fitting into defined, conserved slots in the protein structure. The most dramatic illustrations of this fact came initially from the complexes of the electron transport chain. One of the first of these to be obtained at high resolution was the structure of mammalian cytochrome *c* oxidase, also known as Complex IV (83), in which many lipids were resolved, with up to 13 bound to each monomer of ∼200 kDa molecular weight. These lipids, characterized by mass spectrometry, were found to be specific not only in their placement, occupying the same positions in each preparation, but also in head group and tail length and degree of saturation (82) (Fig. 2). Many of the same lipid-binding sites were also found to be conserved in structures of bacterial cytochrome *c* oxidase (84, 85, 86, 87). In the bacterial enzyme structure, several of the lipid grooves were occupied by detergent (84), demonstrating that the lipid/detergent-binding sites play important and specific roles in the native protein structure. Similar results were found in crystal structures of mammalian and yeast cytochrome *bc*_*1*_ (Complex III of the electron transport chain) (88, 89, 90) and the plant homolog, cytochrome *b*_*6*_*f* (91, 92). The retention of resolved lipids in conserved positions emphasizes the importance of lipid and detergent structure in the maintenance of the native state.

As a variety of new, pure, chemically defined detergents became available (93, 94), including the successful sugar-based decyl and dodecyl maltosides (95, 96) and many more designs (94, 97), the field moved rapidly forward despite the challenging fact that proteins buried in membranes often have flexible conformations required for their complex roles as receptors and transporters (98, 99). Although achieving very high-resolution structures (<2 Å) remains difficult, high resolution is essential where function involves conformational change or depends on the positioning of water. Again, the large multisubunit complexes of the mitochondrial electron transport chain have provided insightful success stories, in part due to their distinct spectral characteristics that aided purification of native forms. These membrane-embedded, spectrally unique proteins were among the first to be structurally defined at high resolution, after the groundbreaking achievement of the bacterial reaction center by Deisenhofer *et al.* (42), and helped move the field of bioenergetics into a new era of mechanistic understanding. The structures of these large complex proteins revealed the exact positioning of their many prosthetic groups, enabled the subunit composition to be confirmed, and allowed meaningful interpretation of spectral changes and fast kinetic analyses of electron transfer events. Their unique UV–visible characteristics were powerful tools for assessing the native state of the purified proteins and for analyzing the rates and ordering of electron and proton transfer events. Without structures that showed water positions and sometimes distinct hydrogen-bonded water chains (84, 100), their kinetics could not be definitively interpreted. High-resolution structures led to the clarification of the proton transfer process, by the Grotthus mechanism, first suggested in 1806 by Theodor von Grotthuss (101, 102) and brought us to our current understanding of how coupling of electron transfer, oxygen reduction, and proton transfer leads to energy conservation in most living organisms. But the supreme success story from the bioenergetics field was capturing the machinery that completes the energy transducing process, the ATP synthase (56) whose structure enabled the deciphering of how rotational motion could be driven by the proton motive force to generate ATP. Beautiful structures of this amazing machine from the Walker group and others, initially without its membrane-embedded region but still the largest asymmetric protein structure known at the time, continue to reveal new and surprising aspects of how energy is transduced (103, 104, 105).

With the understanding of the importance of specific lipid interactions came increased efforts to create more lipid-mimetic detergents (97) and to devise methods of retaining native lipids and lipid-like molecules in the purified MPs. One of the successful approaches was creating a lipidic “cubic phase” (LCP) or meso-phase (22, 106, 107) that involved mixing a specific ratio of lipid (such as monoolein) and aqueous phase to form a membranous lipid organization into which the protein could insert, maintaining a membrane-like environment. Otherwise difficult-to-crystallize proteins are able to diffuse and form well-diffracting crystals in this phase, with many success stories (107, 108, 109) including bacteriorhodopsin, the thermophilic covalent cytochrome *c*-cytochrome *c* oxidase complex (110), and many GPCRs (111) (see review by Stevens *et al.* in this issue).

Another important achievement stimulated by the need for specific lipid interactions was the design of bicelles and nanodiscs (112, 113) and surrogates to membrane lipids such as amphipols (114, 115). Nanodiscs were created by encircling a small portion of lipid and a captured MP using scaffold proteins derived from apolipoproteins (113) or with chemical polymers such as styrene-maleic acid (116). Bicelles are similar but are self-assembling small model membrane systems edged by bile salt lipids. Bicelles were initially designed to aid in aligning MPs in a magnetic field suitable for NMR analysis (112). Nanodiscs, with added stability and control of size, have been useful for many types of analysis including single-molecule studies, activity studies, and resolving aggregation state issues, as well as facilitating the use of cryo-EM. This latter major technical development has further enabled MP structure determination, with the use of single-particle cryo-EM and the nanodisc method showing great success (117).

A revolution in cryo-EM detector methodology and computational analysis has facilitated new levels of resolution of very large complexes in different conformations even in a single sample (Fig. 1). Again, the electron transfer chain has been the subject of dramatic early success stories, with the most impressive being complex I (118, 119, 120), the largest respiratory chain component with 45 subunits. These structures also show specifically bound lipids and reveal the likely proton routes through the membrane segment. Having the Complex I structure was essential, in turn, to solve the atomic resolution structures of supercomplexes up to 1.7 MDa.

The story of supercomplexes of the electron transport chain is a long and controversial one (121, 122, 123, 124). It required the new high-resolution cryo-EM technology to finally convincingly establish their existence. But their functional significance remains much debated (125, 126, 127). One key to successfully defining the atomic resolution structures of these unusually large assemblies has been the availability of X-ray structures of all the components, along with sophisticated computational fitting methods. A number of different assemblies have been discovered (117, 121), some with Complexes I-IIIx2-IV, others with two of complex IVs (but not associated as a dimer). A recent version of the human supercomplex denoted the megacomplex (128), which shows an assembled dimer of two supercomplexes and suggests a place into which Complex II, the only missing member of the chain, would fit. The continuing issue of the functional significance of these assemblies remains. The question of what is the true native assembled state and how dynamic it is contributes to this issue (129). A recent finding is that the cytochrome *c* oxidase (Complex IV) monomer form appears to be significantly more active than the dimer (130, 131). Since Complex IV appears as a monomer in all the supercomplexes so far established, this may be revealing an important new regulatory mechanism.

These amazing structures of the respiratory machinery have led the way in illuminating not only unique protein structures and functions, but also the fundamental role of lipid in MP integrity. Their accessibility through the PDB in the most useful formats continues to allow us to test hypotheses and to excite our imagination, stimulating new modeling efforts and new concepts in the field of bioenergetics and pushing the boundaries of our understanding of energy metabolism in health and disease. These early results provided the initial demonstration that it was possible to obtain crystal structures of MPs of even massive dimensions and expanded our understanding of lipid environments that were needed to permit this achievement.

## How do membrane proteins accomplish key physiological functions?

Residing in the boundaries of cells and their organelles, MPs and their complexes underpin many important aspects of cell physiology that can be addressed through structures held in the PDB. A representative repertoire of these physiological functions includes how MPs are correctly chaperoned and inserted into their correct host membrane, how some proteins are secreted across membranes. How is osmotic balance maintained by water channels that do not leak any protons or ions, and how ions are specifically passaged by gated channels? How are cells held together in surfaces of the skin and organs with appropriate paracellular transport? The bioenergetic transformations required for ATP synthesis (respiratory electron transfer complexes, ATP synthases), maintenance of membrane potential (P-type ATPases), production and control of ion gradients (ion channels), sequestration and export of key regulators such as calcium ions (sarcoplasmic reticulum and plasma membrane calcium ATPases), transport of nutrient, toxin and xenobiotic through primary active transporters driven by ATP hydrolysis (ABC transporters) and secondary active transporters driven by proton plus electrochemical gradients. We summarize the MFS transporters and also describe viroporins (VPs), small viral MPs that form channels necessary for virulence. These structures offer routes to drug discovery that are often broader than simply inhibition, such as modulation or even enhancement of activities. While the examples we have chosen illuminate the value of the PDB in providing structure–function insight into basic biological phenomena and their possible application, our goal is to provide an instructive vision rather than a comprehensive oversight.

### How are membrane proteins targeted, inserted, and folded correctly?

How do MPs come to be? How do MPs find their final destination and fold properly in a lipid bilayer? This “*chicken and the egg*” question has been partially answered over the last 50 years since the PDB was established in 1971. Like all proteins, MPs begin their journey as nascent polypeptides that emerge from the ribosome with their final destination encoded in their primary structures. Following the discovery that proteins have intrinsic signals encoded in several amino acids near the emergent N terminus, that govern their transport and localization in the cell by Sabatini and Blobel in 1971 (132), a large body of biochemical and functional studies have identified the machinery and dissected the steps involved in the sorting, targeting, and chaperoning of MPs through the cell toward their final destination. Experimental validation of the “signal hypothesis” led to powerful and widely used computational methods aimed at predicting the diverse protein-sorting signals (133) for subcellular compartments or organelles such as the endoplasmic reticulum (ER), mitochondria, lysosomes or chloroplasts, and other plastids in all living organisms.

The structural biology of MPs has not only provided remarkable insights into the architecture and mechanisms of action of channels, transporters, pumps, and receptors but also elucidated key aspects of the molecular machinery involved in their biogenesis: from the targeting and sorting as they emerge from the ribosome until their translocation across or insertion into biological membranes. The translocation/insertion step is catalyzed and assisted by translocons, a general term referring to the MP machinery facilitating this process. The Sec61/SecY *universal* translocon (Sec standing for *Secretory*) is central to the translocation of most membrane and secreted proteins while other “specialized” translocons can be highly substrate-specific.

Most proteins are thermodynamically stable in their native state. The process of translocating a polypeptide across or into a membrane is thermodynamically unfavorable. This is due to the large entropic costs associated with *i)* moving polar or charged amino acids across the inner hydrophobic part of the lipid bilayer and, conversely, *ii)* moving hydrophobic amino acids across the outer membrane surface layers consisting of the phospholipid polar head groups (134, 135). It is particularly significant for integral MPs with large soluble loops or domains and complex topological arrangements of hydrophobic TMs. In the last two decades, the PDB has been the repository of increasing numbers of structures of soluble and membrane proteins that help MPs reach their final destination and fold properly, thus contributing to deciphering the general principles and mechanisms underlying “protein-assisted” MP biogenesis.

#### The signal sequence-dependent universal targeting pathway of membrane proteins

**Figure 3:**
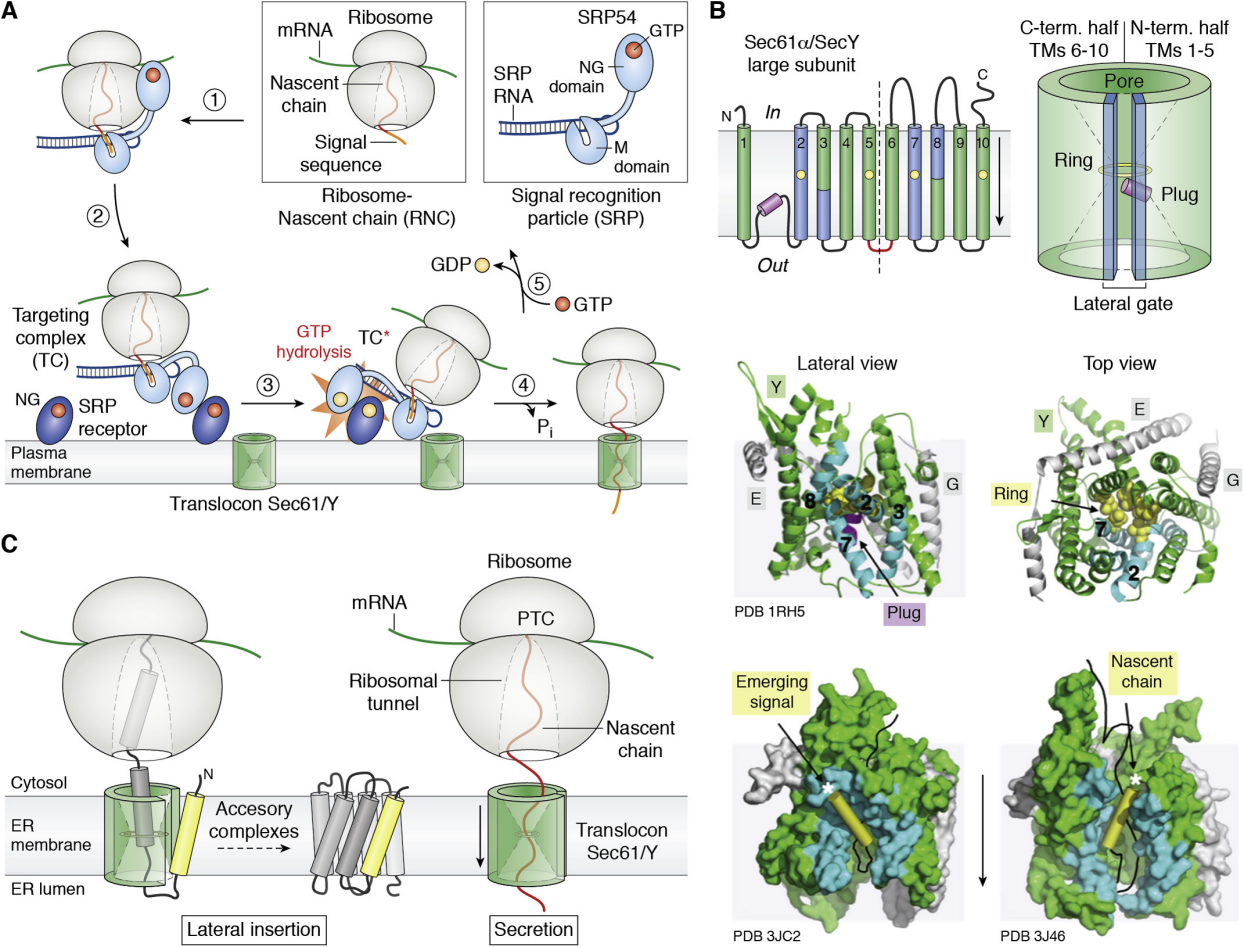
**The machinery of the cotranslational secretion/membrane insertion pathway.***A*, the SRP-dependent protein targeting pathway (in bacteria). (1) SRP binds the signal sequence as it emerges from the RNC. (2) GTP-dependent formation of the TC between SRP and its SR receptor at the membrane delivers the RNC to the translocon (3). The translocon stimulates rearrangement and GTP hydrolysis within the TC and transfer of the RNC from the now activated TC∗ to the channel (4) for subsequent secretion or insertion of the translated protein. (5) GTP displaces GDP in SR and SRP to reset the targeting machinery. *B*, topology and schematic structure of the protein-conducting Sec61α/SecY large subunit. The ring, plug, and lateral gate of the channel open in order to accommodate a TM and provide an exit path into the lateral plane of the bilayer. The structure of the closed archaeal SecY (PDB 1RH5) channel shows where the lateral gate is closed and the pore occluded by the plug helix (*magenta*) and sealed by the ring of hydrophobic residues (*yellow*). Structures of mammalian Sec61 (PDB 3JC2) and bacterial SecY (PDB 3J46) translocons engaged with a nascent chain show the signal sequence (*yellow*) and nascent chain (*black*) emerging from the lateral gate. *C*, models for the lateral insertion of integral MPs in the lipid bilayer or secretion across the membrane. TMs of nascent integral MPs initiate their folding within the ribosome tunnel before moving to the Sec61/SecY translocon. In contrast, an unfolded nascent chain of a protein secreted across the membrane might not trigger opening of the lateral gate. The ring of hydrophobic residues at the constriction of the hour glass-shaped pore maintains a tight seal around the nascent chain. PTC, peptidyl-transferase center of the ribosome. Accessory complexes of Sec61/SecY are required for the insertion and chaperoning of multipass MPs.

Most MPs follow the Signal Recognition Particle (SRP) dependent cotranslational targeting pathway (136). At the core of this evolutionarily conserved machinery is the catalytic and structural SRP RNA associated with the SRP54 protein. It is involved in complex assembly, signal sequence recognition, and nascent chain transfer from the ribosome to the Sec61/SecY translocon (Fig. 3*A*). The presence of an RNA component hints at its ancient origin when RNAs played more prominent roles as catalysts and structural scaffolds in primordial cells (137). Targeting relies on three crucial steps; *(i)* the interaction between the Methionine-rich (M) domain of the soluble SRP54 protein that recognizes and binds N-terminal “greasy” signal sequences as they first emerge from the ribosomal tunnel (138, 139), *(ii)* the GTP-dependent interaction between the NG (for N-terminal and *ras*-like GTPase) catalytic domains in SRP54 and the homologous domains of its membrane-associated SRP receptor (SR) (140, 141) to form a targeting complex (TC) at the ER or plasma membranes resulting in *(iii)* the delivery of the ribosome-nascent chain (RNC) from the SRP54/SR complex docked to the translocon upon structural rearrangement of the SRP54/SR core on its SRP RNA scaffold and the channel-stimulated reciprocal GTP hydrolysis that dissociates SRP from its receptor (142, 143) (Fig. 3*A*).

The conserved heterotrimeric channels, Sec61*αβγ* in eukaryotes or SecYEG in prokaryotes, act as portals and gatekeepers for the insertion of most of the MPs and secreted proteins. The large subunit forms the protein-conducting pore itself and is composed of ten TMs, where the first five TMs are related to the second five TMs by a twofold quasi-symmetry in the plane of the membrane (144). This internal pseudo-symmetry divides the channel into two halves articulated around a hinge between TMs 5 and 6 (Fig. 3*B*). This channel has a unique architecture with two conducting paths oriented perpendicular to each other: An hour-glass-shaped pore for the secretion of proteins across the membrane and a lateral gate opening to chaperone TMs of integral MPs destined for insertion into the bilayer and delineated by TMs 2, 3, 7, and 8 (Fig. 3*C*). The protein-conducting pore is occluded by a short plug helix and sealed by a ring of hydrophobic residues (Leu, Ile, and Val) at its constriction point that prevent leakage of water and ions (145). Structures of free SecY channels with their lateral gates in different states (146, 147) and structures of Sec61/SecY channels engaged with different RNCs beautifully illustrate the insertion path followed by nascent TMs of integral MPs during their biogenesis (148, 149, 150). The funnel-shaped cytoplasmic vestibule of the translocon is large enough to accommodate incoming folded helices while soluble loops can extend at the interface between ribosome and channel. The *α*-helical signal sequence of the nascent chain emerges, opening the lateral gate as it displaces and supplants TM2. As lateral gate TMs shift, the ring and plug helix are widened and displaced/unfolded, respectively, to make room for this nascent chain that contains a folded secondary structure (Fig. 3*B*).

While the Sec61/SecY protein-conducting channel provides a clear path across or into the membrane, it is not the sole arbitrator of MP insertion topology. Proteins start to fold as soon as space allows, and the ribosome also plays an important role as the nascent chain can either fold or not, while it elongates from within the ribosomal exit tunnel into the Sec61/SecY channel (151, 152). Thus, *in vivo*, rather than start folding inside a lipid bilayer environment, MPs begin this process within the ribosome tunnel and the Sec61 translocon, where secondary structure elements can form and associate into smaller kernels of tertiary structure (153) and where, perhaps more importantly, the insertion topology is assigned as the nascent polypeptide enters into the membrane plane by lateral diffusion. Prokaryotic and eukaryotic integral MP topologies generally follow the so-called “positive inside” rule for TM orientation as established by Sipos and von Heijne (154). In this regard, the Sec complex associated with a ribosome forms an *Anfinsen* cage around the nascent chain, surrounding the nascent polypeptides with a protective “enclosure” and environment where folding occurs unhindered, thus preventing aggregation or misfolding (155). Although the folding steps occurring in Sec61 remain poorly understood, lipids do play a role through direct interactions with the nascent MP in the Sec61 channel (156) (Fig. 3*C*).

Numerous structures of Sec61/SecY complexes with other important “accessory” MP complexes such as the Sec62/63, TRAP (TRanslocon-Associated Protein), and OST (Oligo-Saccharyl Transferase) (157) in eukaryotes or SecDF (158) and YidC (159) in prokaryotes have been solved. These structures reveal that the Sec61/SecY translocon is part of a highly dynamic “MP assembly line,” sometimes referred to as the *holo* translocon, with “quality control” mechanisms (160). The human genome encodes about 2500 multipass MPs (such as GPCRs, ion channels, and ABC transporters) of considerable topological complexity. Lateral insertion and folding of such polytopic MPs require the intervention of Sec61-accessory complexes such as the Endoplasmic Reticulum membrane protein complex (EMC). Although our understanding of the mechanisms governing protein secretion and insertion has progressed, the specifics of MP complex assembly into homo- or hetero-oligomers still elude us.

#### The targeting of tail-anchored proteins and new paradigms in membrane protein biogenesis

**Figure 4:**
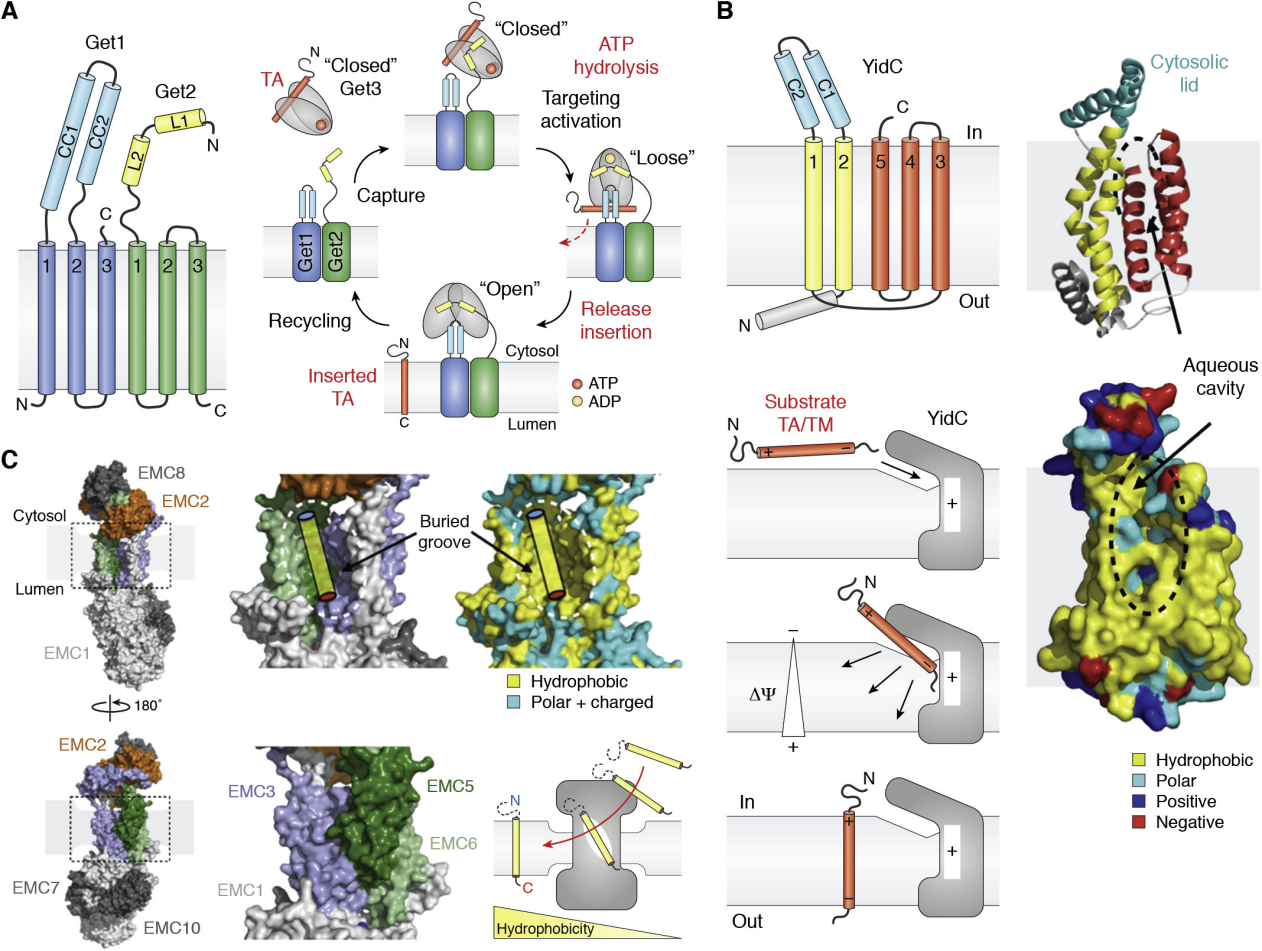
**The Guided-entry of tail-anchored proteins pathway.***A*, simplified mechanism of the Get pathway and topology of proteins Get1 and Get2. *B*, bacterial YidC structure (PDB 6AL2) showing a lipid-exposed hydrophilic groove between TMs 1, 2, and 5 that can be cross-linked to nascent TM-containing substrates. A simplified mechanism for YidC (and possibly Get1/2)-assisted insertion of TMs and TA segments into the membrane. For bacterial YidC, membrane thinning and membrane potential facilitate and drive insertion. *C*, the EMC (PDB 6WW7, 6WB9, 6Z3W) translocates multipass MPs and some TA proteins. TMs follow a gradient of increasing hydrophobicity from the hydrophilic cytosolic vestibule into the intramembranous hydrophobic groove acting as a lateral opening for insertion into the lipid bilayer.

Most membrane-inserted proteins harbor an N-terminal signal sequence, which defines the main route for protein targeting and secretion/insertion. A significant subset of MPs uses different routes and distinct protein machineries. Tail-anchored (TA) proteins (proteins with a single C-terminal TM anchor) constitute about 5% of any given eukaryotic proteome and are targeted with exquisite specificity to the diverse organelles (*e.g.*, ER, mitochondria, lysosomes) within the eukaryotic cell. The yeast Guided-entry of tail-anchored proteins (Get) pathway, known as TRC in mammals, was discovered in 2008 (161). It represents a perfect example of a posttranslational MP targeting system in the ER. TA proteins do not follow the SRP targeting pathway because they lack an N-terminal signal sequence. Their targeting signal consists of their single C-terminal hydrophobic TM, which only emerges from the ribosomal channel once the rest of the polypeptide has been synthesized. In yeast, the Get pathway involves at least six proteins. Among these, the soluble ATPase Get3 and the two MPs Get1/Get2 are involved in the final steps of TA targeting to, and insertion into, the membrane, respectively (162, 163, 164). At the late stage of Get targeting, a Get3 dimer binds the TA of a newly translated TA protein in a hydrophobic groove rich in flexible methionine residues (165), much akin to the M domain of SRP54. The long N-terminal cytoplasmic end of Get1 captures the Get3/TA complex and targets it to the membrane where the cytoplasmic coiled-coil extension of Get2 interacts with the docked Get3/TA complex to stimulate the release of its TA cargo before insertion into the membrane (166) (Fig. 4*A*).

The exact mechanism of TM insertion by Get1 (with Get2) has yet to be elucidated. However, Get1 is a member of the YidC/Oxa1/Alb3 superfamily of proteins that insert proteins into membranes in bacteria, mitochondria, and chloroplasts, respectively (167). This superfamily not only includes the bacterial insertase YidC but also the widely conserved ER membrane complex (168, 169, 170), which is involved in the cotranslational insertion of polytopic MPs, such as GPCRs, in association with the Sec61 translocon, and also the posttranslational insertion of some more hydrophilic TA proteins. In particular, EMC assists Sec61 with the insertion of MPs with complex topologies and destabilizing features in their TMs such as charged residues (171). Remarkably, the structure of another Sec61-associated complex containing five accessory factors (TMCO1-CCDC47 and Nicalin-TMEM147-NOMO), distinct of EMC but also involved in the biogenesis of hundreds of multipass MPs was recently solved (172). TMCO1 is also a member of the YidC/Oxa1/Alb3 superfamily. The structures of these machineries involved in complex MP biogenesis were determined within the last 2 years.

Unlike the posttranslational insertase Get1/2, YidC, and EMC cooperate with the SecY/Sec61 translocon for the insertion of MPs. Based on the structure of bacterial YidC (173), a model for the insertion of TMs and TAs has emerged. A “hydrophobic slide” is created between TMs 1, 2, and 5, while the hydrophilic environment generated by the groove can recruit the extracellular regions on substrates into the low-dielectric environment of the membrane, thus facilitating insertion (Fig. 4*B*). Furthermore, the protein architecture and its interactions with membrane phospholipids result in an asymmetric thinning of the membrane on the cytoplasmic side near the aqueous membrane cavity (174).

The nine-subunit structure of EMC reveals that TMs from subunits EMC3, 5 and 6, form a lumen-sealed lipid-exposed intramembranous groove large enough to accommodate a single TM, similar to YidC. Furthermore, protein translocation involves a cytosolic hydrophilic vestibule (EMC2/EMC8-9) at the interface between EMC2 and EMC3, EMC5 and EMC6, and methionine-rich loops on EMC3 to probably accommodate the bulkier and more hydrophilic ends of TA proteins and capture their hydrophobic TA, respectively. EMC uses spatially distinct yet coupled regions including lipid-accessible membrane cavities and cytosolic surfaces to function as an insertase for TA proteins and a protein holdase-chaperone for complex polytopic MPs (175). Increasing hydrophobic interaction between the hydrophilic cytoplasmic vestibule and the buried hydrophobic membrane groove guides the translocating TAs for insertion (170). Membrane thinning around the EMC further lowers the energy barrier for translocation (Fig. 4*C*). While the Get machinery targets client proteins with highly hydrophobic TAs, the EMC seems to prefer TA proteins with lower hydrophobicity. Thus, triage of TMs and TAs into the different insertion machineries (*i.e.*, EMC *versus* Get) relies, at least in part, on their relative hydrophobicity (171, 176). These recent structures illustrate the roles of electrostatics, hydrophobicity, and protein architecture in establishing the topology of TM translocation through shaping and thinning of the surrounding membrane to facilitate insertion.

#### Folding it backward: The case of *β*-barrel transmembrane proteins

Helical MPs are ubiquitous in prokaryotes and eukaryotes. Transmembrane *β*-barrel proteins constitute another functionally important class of integral MPs composed mostly of membrane-spanning *β*-strands; they are exclusively found in the outer membranes of Gram-negative bacteria and the membranes of mitochondria, chloroplasts, and other plastids in eukaryotes. The transmembrane *β*-barrel scaffold is based on an antiparallel sheet of *β*-strands (usually an even number) arranged into a cylindrical structure delineating a central and functional pore or cavity. The first transmembrane *β*-barrel protein structures, PhoE and OmpF, were solved in 1991 to 1992 by the Jansonius/Rosenbusch and the Schulz groups (177, 178). Because of their cellular localization, the biogenesis of *β*-barrel proteins is essentially posttranslational (179). What about membrane insertion of *β*-barrel proteins?

**Figure 5:**
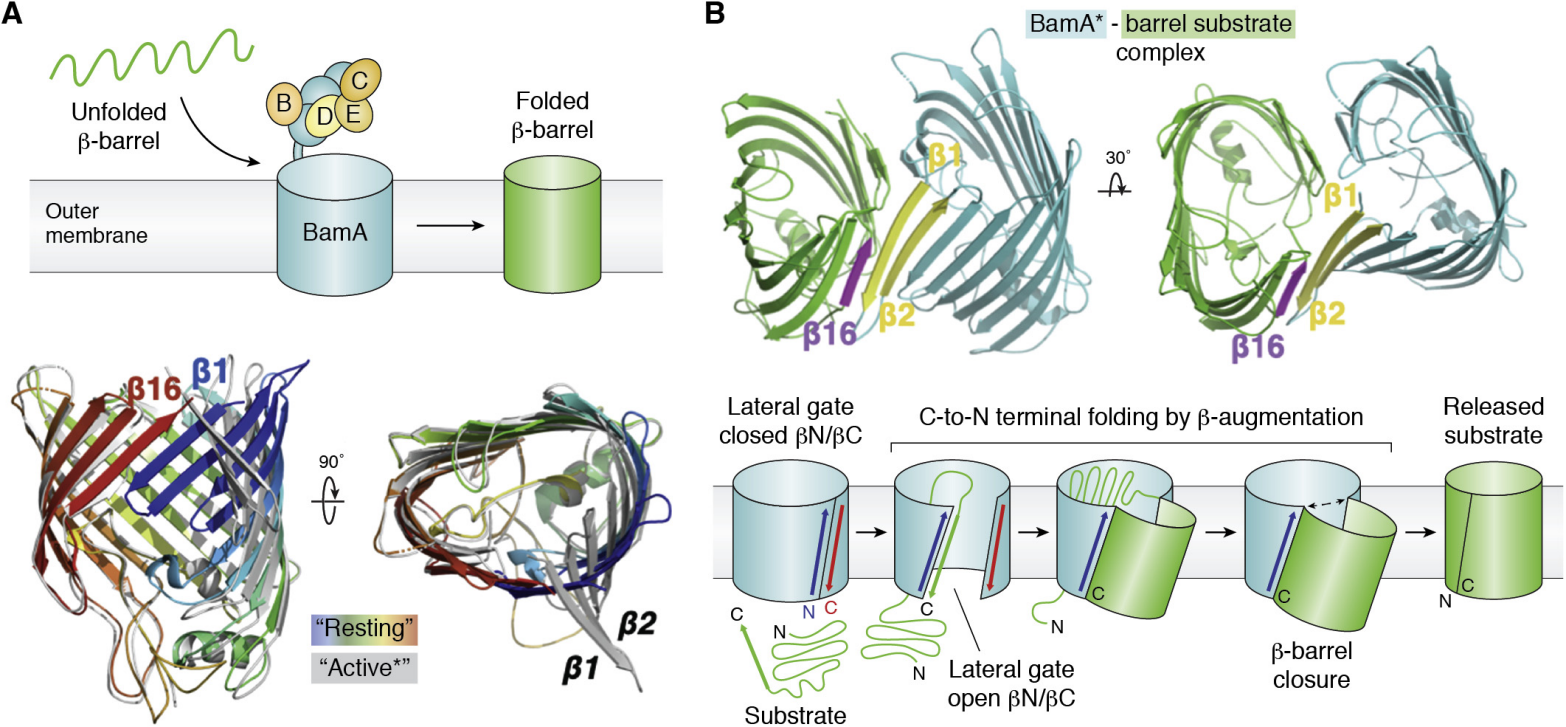
**Bacterial *β*-barrel membrane proteins biogenesis by BAM.***A*, the BAM complex catalyzes posttranslational insertion of transmembrane *β*-barrel proteins from the periplasm into the bacterial outer membrane. Superposition of the *β*-barrel domain of BamA in the resting (PDB 4N75) or active (∗) states (PDB 6V05) with a nascent *β*-barrel protein. *B*, C-to-N terminal *β*-barrel folding by sequential *β*-augmentation.

The bacterial *β*-Barrel Assembly Machine (BAM) (180), related to the mitochondrial Sorting and Assembly Machinery, accelerates folding without using exogenous energy (such as ATP or membrane potential). A recent cryo-EM structure (181) reveals a “*templating*” mechanism based on sequential *β*-augmentation for the folding and insertion of *β*-strands into membranes and the growth of the cylindrical *β*-barrel structure. The BAM complex is composed of the membranous subunit BamA and four lipid-binding soluble proteins (BamBCDE) (180, 182). The central component BamA is itself a *β*-barrel protein that forms a closed 16 *β*-stranded antiparallel barrel when not engaged with a substrate (Fig. 5*A*).

The structure of BamA trapped with a model *β*-barrel substrate (a modified version of its barrel) shows that the active BamA barrel is splayed open along its lateral gate, exposing the two N-terminal strands *β*1 and *β*2. The N-terminal *β*1 strand no longer hydrogen bonds with C-terminal strand *β*16, as seen in the resting structure of folded BamA. Instead, it forms six hydrogen bonds with the C-terminal strand *β*16 of its barrel substrate protein (Fig. 5*B*). However, the resulting hybrid barrel is asymmetric since the C-terminal strand *β*16 of BamA does not pair with the N-terminal *β*1 strand of the barrel substrate. By binding the N-terminal edge of BamA as an extended *β*-strand, the C terminus of the substrate forms a new edge that serves as a template to guide binding and folding of the subsequent *β*-strand by *β*-augmentation. As a consequence, folding is directional and proceeds from C- to N terminus (Fig. 5*B*). Interestingly, the six hydrogen bonds between strands in the membrane environment form a very stable interface between the two proteins, BAM and the nascent *β*-barrel substrate. Such stability enables folding but results in a high kinetic barrier for substrate release once folding is complete. Sequence features at each end of the substrates overcome this barrier and favor substrate release by stepwise exchange of hydrogen bonds (181). More recently, two cryo-EM structures of the Sorting and Assembly Machinery, structurally and functionally related to BAM, also revealed an opening of the lateral gate as substrates get inserted and suggested that entire precursor proteins might fold using a *β*-barrel switching mechanism (183, 184).

The general dogma is that translocons must harness some form of energy to lower the energetic barrier associated with the translocation of a polypeptide and to function as translocases and/or insertases. The most common energy sources for such processes are the binding and hydrolysis energy of nucleotides (*i.e.*, ATP and GTP) and also proton gradients or membrane potential that can impart directionality. In particular, ATPases such as the SecA ATPase, which energizes the SecY-dependent posttranslational protein secretion in bacteria (185), or AAA^+^ ATPases specific to secretion systems found in pathogenic bacteria or protozoa, couple ATP binding and hydrolysis energy to generate mechanical forces used to unfold and translocate their substrates. These translocons use so-called polypeptide “clamps”; they engage the polypeptide main chain nonspecifically and keep it unfolded as it is threaded through a proteinaceous pore traversing the membrane. The recent structures of the EMC and BAM machineries reveal in exquisite detail how protein-assisted translocation and insertion combine mechanisms such as *membrane thinning, hydrophobic sliding*, and *protein-templating* to decrease the energetic barrier associated with translocation across or insertion into the bilayer of a polypeptide without apparent expenditure of energy.

#### Most extreme translocation: Effector protein and virulence factor secretion in pathogens

**Figure 6:**
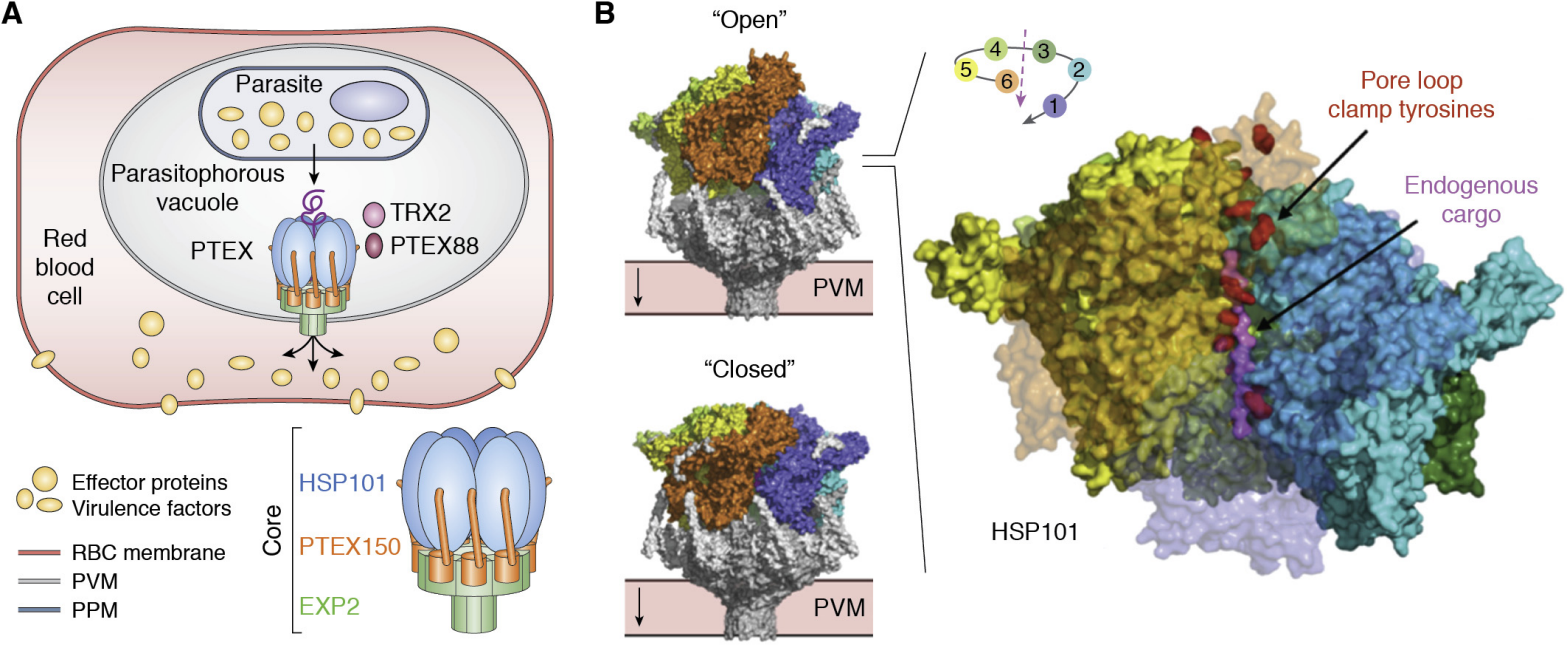
**The *Plasmodium* translocon of exported proteins acts as the essential nexus for protein export and host cell remodeling by the malaria parasite.***A*, effector proteins and virulence factors are secreted across the encasing parasitophorous vacuole membrane (PVM) inside the host red blood cell (RBC) by the genus-specific *Plasmodium* Translocon of Exported proteins (PTEX) whose core is composed of subunits EXP2, PTEX150, and HSP101. *B*, cryo-EM structures of two distinct states (PDB 6E10, 6E11) of the ternary PTEX core complex. Both are trapped with *endogenous* cargo polypeptide engaged in the translocation path of the spiral staircase hexameric assembly of HSP101 (rainbow subunit-coloring pattern) sitting atop of the membrane inserted PTEX150/EXP2 tetradecamer (*gray*). The labile auxiliary subunits TRX2 and PTEX88 were not present in the final reconstructions. Slice-through view of the HSP101 ATPase in the open state showing endogenous cargo (*pink*) clamped by each tyrosine of the pore loops (*red*) arranged into a spiral staircase.

The journey of some secreted proteins across the cell can be more arduous in the extreme cases of intracellular pathogens such as the deadly malaria parasite *Plasmodium falciparum (Pf)*. Malaria is a disease mentioned in Sumerian and Egyptian ancient texts (1550 BC). In 2018, *Plasmodium* infected some 230 million humans and claimed about 435,000 lives. Upon infection of its human host, this obligate intracellular parasite dwells within a parasitophorous vacuole derived by invagination through the membrane of infected hepatocytes or erythrocytes. *Plasmodium* is a master cell renovator; it exports hundreds of effector proteins and virulence factors across the PV membrane into the host cell in order to gather nutrients, eliminate waste, persist and thrive in its host, and evade the immune response (186, 187). Some of these exported proteins belong to a *Plasmodium* “transportome” (188)—a compendium of transporters, pumps, and channels involved in ion flux, nutrient/metabolite import, and waste/drug efflux within the parasite and the infected host cell such as the hexose transporter *Pf*HT1 and the chloroquine-resistance transporter *Pf*CRT. In 2009, de Koning-Ward *et al.* (189) discovered the complex responsible for vacuolar secretion and named it *Plasmodium* translocon of exported proteins (PTEX) (Fig. 6*A*).

PTEX is composed of three essential core subunits: The pore-forming protein EXP2, the AAA^+^ protein unfoldase HSP101, and the adaptor PTEX150. Two accessory subunits, PTEX88 and TRX2, complete the assembly (190, 191). Its cryo-EM structure revealed the architecture of its ternary core (192), including the novel MP structure of EXP2 that forms the protein-conducting pore (Fig. 6*B*). Most of the effector proteins are essential to parasite survival. Thus, the vacuolar secretion pathway, with its *Plasmodium*-specific and unique vacuolar translocon, provides prime targets for the design of novel antimalarial drugs (191, 193, 194).

The 1.6 MDa PTEX complex solved by Ho and Beck was obtained from endogenous sources, expressed at its natural (low) abundance, and extracted directly from human red blood cells infected with *Plasmodium* parasites CRISPR/Cas9-edited to express HSP101 bearing an affinity purification tag at its C terminus. While purification from endogenous sources is not a novel strategy *per se*, the clever use of CRISPR/Cas9 gene editing by Beck and Goldberg (195) on an “unruly” organism such as *Plasmodium*, traditionally recalcitrant to facile genetics, opens new frontiers to tackle the structures of scarcely available complex molecular machines that could not be reconstituted using more traditional approaches based on recombinant expression. Furthermore, extraction of the endogenous PTEX structure in the presence of the slowly hydrolysable nucleotide analog ATP*γ*S allowed the complex to be caught “in the act” under two distinct states engaged with endogenous cargo trapped in the translocation channel of the HSP101 ATPase (Fig. 6*B*). It provided valuable insights into the mechanism of effector secretion. PTEX is an example of purely posttranslational translocon and, in contrast with Sec61/SecY, it seems devoid of insertase function and exclusively provides a path for posttranslational secretion across the vacuolar membrane (191). This example illustrates the power of a “shotgun” approach combining cryo-EM (or cryo-ET) and Mass-Spectrometry-based proteomics to isolate, identify, and resolve ensembles of large macromolecular assemblies in complex mixtures such as crude cellular extracts (196).

Each of these structures brings us back to our “*chicken and the egg*” problem. During the emergence of life from primordial macromolecular systems, it is likely that primitive MPs spontaneously inserted in simple lipidic bilayers. Spontaneous insertion of very hydrophobic TMs has been observed for MPs with relatively simple topologies. The emergence of a translocon with its accessory factors and protein quality control machinery probably paralleled increasing complexification of MP folds, cellular compartmentalization, and the associated evolutionary pressure to evolve more sophisticated and dynamic proteinaceous systems to catalyze and chaperone their folding and proper biogenesis. In-depth analyses of the YidC and SecY structures and of the requirements for the biogenesis of different classes of integral MPs and secreted proteins shed light on the origins and evolution of the most ancient “translocators.” Lewis and Hegde speculate that SecY originated as a YidC homolog formed of two hydrophilic grooves juxtaposed within an antiparallel homodimer (197). The proposed “molecular filiation” between the two major MP biogenesis factors offers a new perspective on a fundamental step in the evolution of macromolecular biological systems and cells.

### Water transport by aquaporins and ammonia gas transport by the Rh family

The AQPs provide a striking example of how, over two decades, the PDB coordinated structures into functional knowledge. Because life depends on water, regulation of water movement across cells without leakage of any ions or protons is an essential function. It is encoded in a family of highly regulated water channels that are expressed in the organ, tissue, and cell-specific locations (198). By the 1960s, there was evidence that water permeated membranes faster than through lipid bilayers. Red blood cells conducted water with a low activation energy barrier, while oocytes had very high resistance to conductance. Benga and colleagues showed in 1986 that this water conductance could be inhibited by *p*-chloromercuribenzoate. The reversal of this inhibition by reducing agents implied a protein channel that contained a sulfhydryl accessible to mercury ions (49, 50, 199). While radiation inactivation correctly predicted a channel of ∼30 kDa in size (200), Agre (201), in a search for Rh blood group antigens in highly porous red blood cells, found two protein bands—one of 32 kDa and the other 28 kDa. Expression cloning in frog oocytes of the 28 kDa “Channel-like Integral membrane Protein of 28 kDa” (CHIP28) (202, 203) from an erythroid library showed that transfected oocytes responded to reduced osmolarity by swelling and lysing, while control oocytes barely swelled at all (48). The gene responsible was shown to be similar to others in microbes and plants (204), and Agre named the family the Aquaporins. CHIP28 became AQP1. It was shown to be a tetramer (205, 206) with a permeability estimated at a remarkable 3 × 10^9^ water molecules per second (207, 208), which equated to the diffusion limit for a pore the size of a single file of water molecules. More AQP homologs were discovered and the family provided essential functions represented in all living species from bacteria to humans (198, 209).

In the same family, the aquaglyceroporins that conduct small organic alcohols including glycerol as well as water are interesting because of the clear need for this function (210). AQP3 is permeable to glycerol and water while AQP9 has even broader specificity. There is also an ion-conducting AQP. AQP6 conducts water poorly and is unique in conducting ions through the same water-conducting channel, supported by altered sequence in the pore, or possibly through the fourfold axis of the tetramer (54, 211).

**Figure 7:**
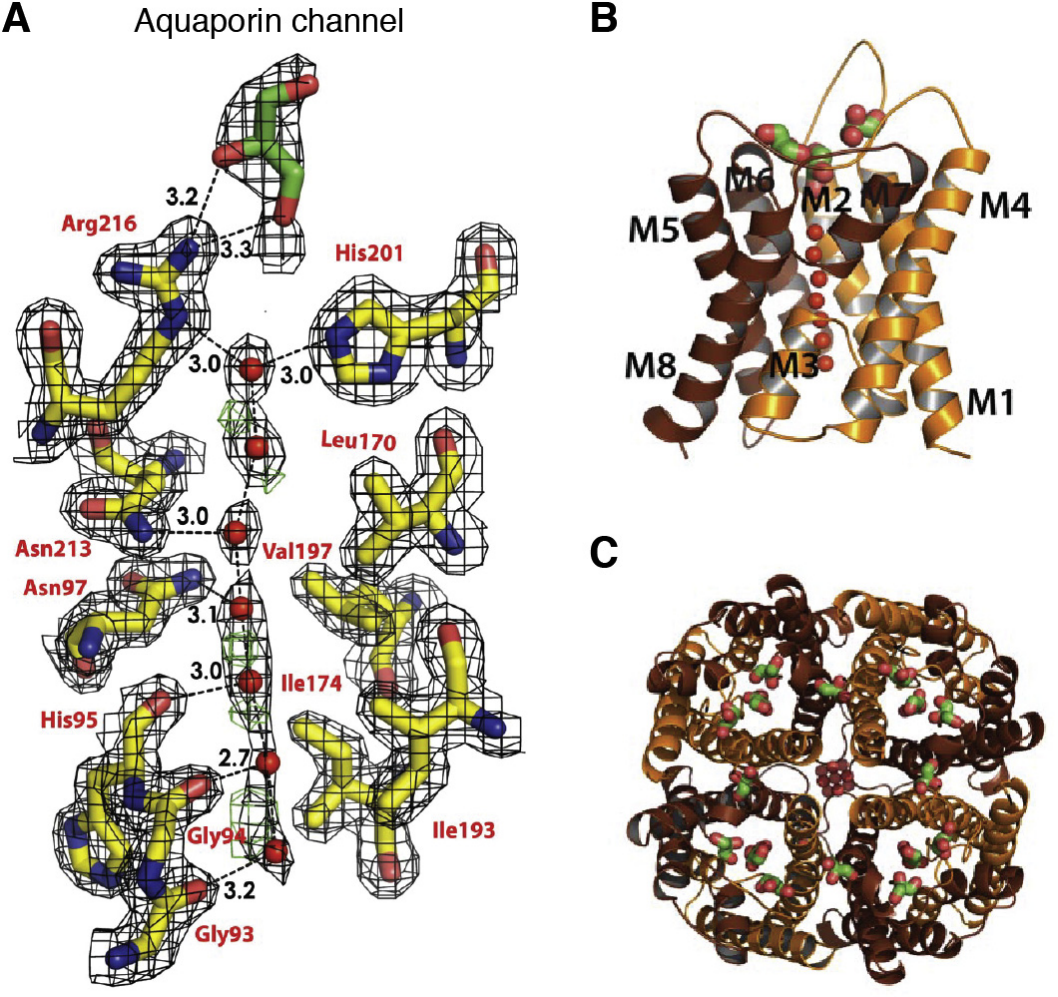
**The aquaporin channel.***A*, line of waters in human AQP4 at 1.8 Å resolution (PDB 3GD8). *B*, single monomer of AQP4. *C*, the tetrameric arrangement in all AQPs places four channels in each tetramer. Panels derived with permission of the PNAS from Ho *et al.* (55).

The first atomic structure of an AQP was determined in 2000 for the Aquaglyceroporin GlpF by X-ray diffraction at 2.2 Å resolution (54). AQP1 was defined by electron crystallography of 2D crystalline arrays of AQP1 at 3.8 Å resolution (53), followed closely by the X-ray structures of AQP1 at 2.2 Å (212, 213) and bacterial AqpZ at 2.5 Å (214). The structures showed AQPs to be fourfold symmetric tetramers in which the water channels lie within each monomer and not between monomers or along the symmetry axis (Fig. 7). There are now ∼60 structures reported in the PDB with some at sub Å resolution (215). AQP structures AQP0 and AQP4 were determined by X-ray crystallography (55, 216) and by electron crystallography (217, 218) of natural 2D crystalline arrays of the tetramers that are found in certain specialized environments. Natural arrays from an ordered region of the eye lens led to ∼1.9 Å structure for AQP0 that showed the position of lipids that supported the crystalline array in membranes.

The structures show an uninterrupted line of permeant waters within each monomeric channel across the entire membrane. These water molecules are hydrogen bonded to each other in line and sideways as hydrogen bond donors to a line of eight carbonyl oxygens of successive amino acids—like pitons that form a line all the way to and from the polarized central pair of water molecules (219, 220). Substrates entering from the outside meet an SF formed by a highly oriented guanidinium side chain of arginine that projects hydrogen bond donors and positive charge into the pore and against a hydrophobic aromatic corner opposite the arginine. This region, termed the aromatic arginine (aR) region SF, distinguishes between water alone and water plus other small substrates. Aquaglyceroporins are specialized by two aromatic side chains that form a hydrophobic corner opposite to the aR in this region to conduct small organic neutral molecules including glycerol and urea as well as water. While compensated for by surrounding acidic side chains, the aR positive charge presents an essential electrostatic barrier to passage of all positive ions (Fig. 7*A*).

The central channel waters are oriented by two highly conserved asparagine N-H hydrogen donors within highly conserved -asparagine-proline-alanine- (-NPA-) regions that abut each other in a “fireman’s grip” structure, to force the central water molecules to be hydrogen bond acceptors and orient their hydrogens always away from the center. This is augmented by the positive dipoles of the two half helices that begin with the -NPA-region and stretch diagonally outward to the two surfaces of the bilayer. This arrangement polarizes the central water molecules so that their hydrogen atoms point away from the center, then polarizing the entire line of water molecules out to the membrane surface (54, 215, 221). Early simulations over ∼300 ps time scales validated these proposals, showing that “turning off” the helix dipoles reduced the tendency to bipolarize the channel waters (219). The absolute rejection of any ions, even hydronium ions or protons, through the line of waters has attracted confirmatory molecular dynamic (MD) simulations up to over 100 ns using increasingly sophisticated empirical valence bond and electrostatic approaches (222, 223).

The 13 AQPs in humans each have highly differentiated roles in physiology. This is even more so in plants with 38 AQPs in *Arabidopsis thaliana* (224)! Genetic knockouts allowed insight into the unrecognized importance of some of these aquaporins (225). AQP structures became robust models for MD simulations due to their range of conductance and specificities for small neutral organic molecules. MD simulations and electrostatic calculations allow understanding of the impact of dynamics, electrostatics, and dehydration penalties in Biology, readily accounting for water conductivity at close to the experimental levels of ∼10^9^ molecules per second, and specificities *e.g*., how conversion of one histidine in the water-only aromatic arginine SF to a hydrophobic side chain allows small neutral organic compounds to pass in specific aquaglyceroporins.

How do the AQPs exclude passage of hydronium ions or protons hopping through the line of waters by the Grotthus mechanism (101, 102)? The theoretical energy profile seen by a proton or a hydronium ion passing through the channel shows that the highest barrier is an electrostatic one where the two -NPA-regions meet, along with the helical dipoles that augment the polarization of the central water (226, 227, 228, 229). Mutations and simulations show that the SF also provides electrostatic repulsion to positive ions or protons (229). However, a hydronium (or any other) ion, on approaching the entry to the channel, needs to be partially dehydrated to enter. This requires a potentially insurmountable large energy cost that would need to be compensated for by the structure of the protein pore (230). In addition when a proton is forced through the channel, the protein requires sufficient time in the simulation to adapt to stabilizing the proton and reduce the apparent barrier at the channel center (231). The negative charge on the membrane surface may also contribute to repulsion of ions. It is argued however that the “reduced solvation” requirement is the dominant energetic term in aquaporins (232).

How is it that other channels also support a continuous line of water molecules and do readily conduct hydronium ion? The Gramicidin D (A, B, C) channels, antibacterial channels consisting of opposed helices of 15 amino acids that meet in the mid-membrane plane, kill cells by freely conduct protons or hydronium ions. So where is the key difference? The carbonyls of amino acids in Gramicidin generally point more outward than in the AQPs and therefore better accommodate the incoming hydronium ion hydration shells (233, 234, 235, 236). While the dehydration cost for ions limits access to these channels too, it is the polarization of waters that defines aquaporin selectivity for water and prevents leakage of any charged ion or protons. Such examples exquisitely illustrate how structural information informs critical aspects of the function that were otherwise not accessible or understandable any other way.

The phylogenetic tree shows that AQPs diverged into the water and the water plus glycerol branches among bacteria and then diverged into its many tissue-specific variants that perform essential functions. The common scaffold provides a remarkable data set of interactions that show how specificity is encoded in subtle variations in structure. The AQPs provide the most intricate insights into the character and properties of water–protein interactions and the barriers to proton transfer through hydrogen bonded lines or waters in all of biology.

Ammonia like water has a similar size and dipole moment as water and its management is crucial in physiology. How is it conducted? Ammonia/ammonium conduction is mediated by a separate ammonia transport family (AmT family in bacteria, Rh family in humans) from a genomic background completely unrelated to the AQPs. AmT/Rh proteins are all trimers of 12 TM proteins (237, 238), in which each monomer conducts neutral NH_3_, although there is a controversy that they might also act partially as ammonium ion transporters. In the AQPs specificity against the ammonium ion lies in the cost of dehydrating the ionic NH_4_^+^ form. The more hydrophobic channel in AmT/Rh helps by destabilizing the ionic form by reducing the p*K*a of ammonium ion from ∼10 to <5 as it enters the Amt pore.

### How do ions cross the membrane? Insights from structures of ion channels

The intrinsic impermeability of the membrane to ions creates two challenges for biology. First, how do ions cross the membrane? And second, how are such movements controlled? As we illustrate here, the availability of the structures of MPs (*i.e.*, ion channels) controlling such processes have played key roles in elucidating how nature’s design harnesses the unique properties of membranes for biological functions.

**Figure 8:**
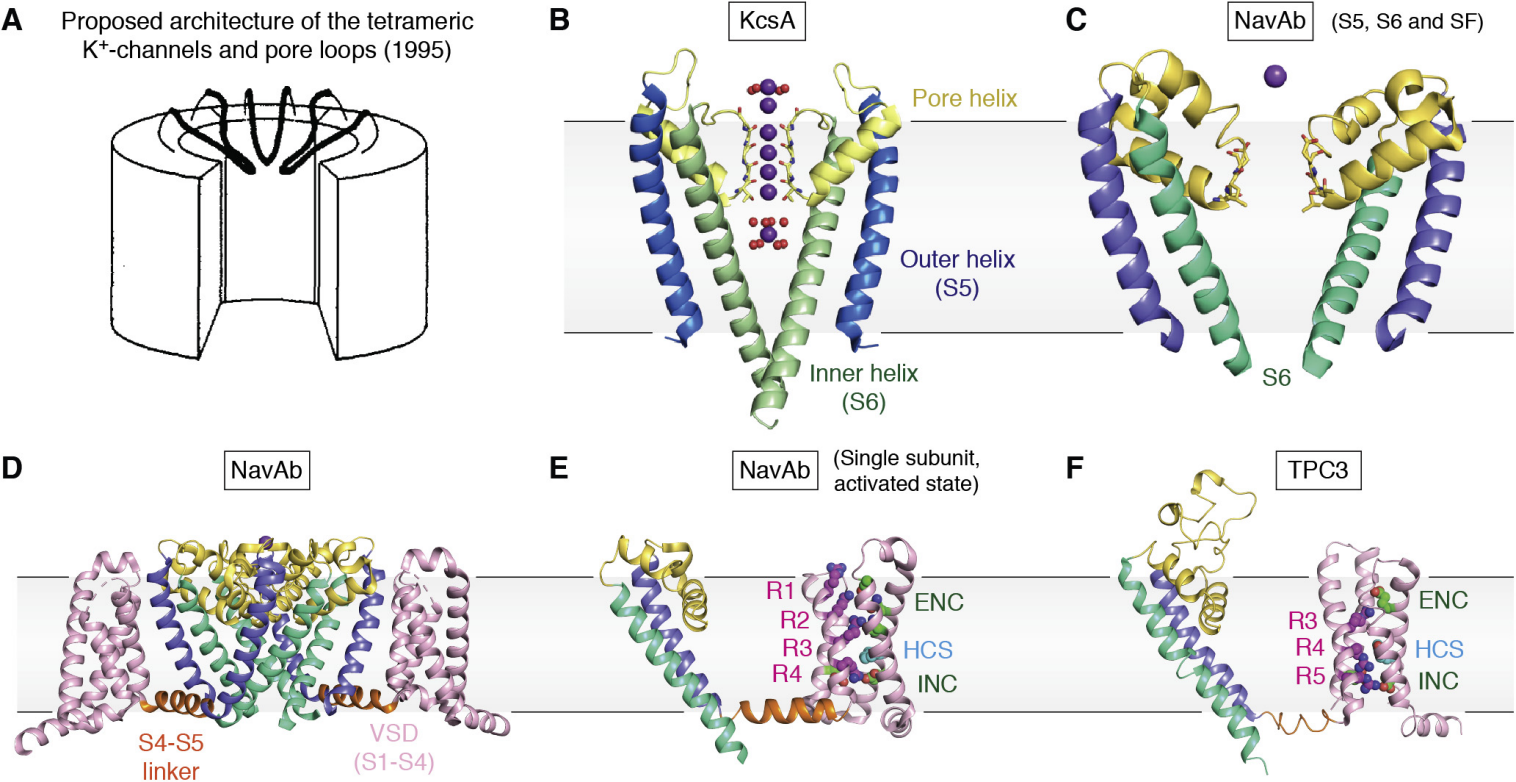
**Architectures of ion channels revealed by structures.***A*, the proposed architecture of the tetrameric K^+^-channels and their pore loops before structures were available (reproduced with permission (263)). *B*, high-resolution crystal structure of KcsA with bound K^+^ ions in and around the SF (PDB 1K4C). Four dehydrated K^+^ ions are coordinated by backbone carbonyls from the SF (Although only 2 K^+^ ions occupy position 1, 3 or 2, 4 in the SF at any given time, all four are observed in the crystal structure as the structure represent the average between the two states). One K^+^ ion coordinated by eight waters is observed right below the SF in the cavity while two more K^+^ ion, one completely dehydrated and one partially dehydrated, are observed above the SF. *C*–*E*, crystal structure of NavAb (PDB 5VB8). Structure of S5–S6 and the SF is shown in (*C*) while the structure of NavAb is shown in (*D*). Water molecules bound to the Na^+^ were not resolved in the structure. For clarity, the two subunits in the front and back are removed in (*B*) and (*C*) while the two VSDs in the front and back are removed in (*D*). The outer helix of KcsA (the equivalent of S5 of Shaker) is colored in *light blue* while the inner helix (the equivalent of S6 of Shaker) is colored in *light green* and the pore region is colored in *light yellow* in (*B*). K^+^ ions are shown as *purple spheres* while waters are in *red*. In panel (*C*–*F*), the VSDs composed of S1–S4 are colored in *pink*; S4–S5 linker is colored in *orange*; S5 is shown in *blue* while S6 is shown in *green*; the pore region is colored in *yellow*. *E*, a single subunit of NavAb in the activated state where R1–R3 are above the HCS. *F*, the functional subunit of TPC3 (PDB 6V1Q) in the resting state where only R3 is above the HCS. The voltage-sensing residues located on S4 are shown as *magenta sticks* in panel *E* and *F* while the acidic residues they interact with at the ENC and INC are colored in *green*. The residues forming the HCS are colored in *cyan*. Membranes are indicated with the *black line*.

Ever since Hodgkin and Huxley described their electrical properties in the 1950s (44, 45, 239, 240, 241), proteins that enable the axon membrane to change its permeability to Na^+^ and K^+^ have been under intense scrutiny. In the 1970s, Armstrong and Hille first demonstrated that Na^+^ and K^+^ cross the membrane through different proteins with pores that select for their respective ions, *i.e.*, the Na^+^-channels and K^+^-channels (242, 243, 244). With the advancements in molecular cloning methods, the genes encoding Na^+^-channels and K^+^-channels were cloned in the 1980s (245, 246, 247, 248, 249). Meanwhile, the ingenious use of natural toxins and small molecules that bind and inhibit K^+^-channels and Na^+^-channels also allowed the groups of MacKinnon, Miller, and Catterall to purify K^+^- and Na^+^-channels and define their architectures (250, 251, 252, 253, 254, 255). Biochemical investigations together with sequence analysis of additional channels plus mutagenesis studies quickly led to the identification of the signature sequences for the pore, the SF, and the gate (256, 257, 258, 259, 260, 261, 262) in the late 1980s. Using knowledge acquired over 40 years by numerous investigators in the field, MacKinnon concluded in 1995 that K^+^-channels must be tetrameric with their pore loops entering the central conductance pathway encircled by the four subunits (Fig. 8*A*) (263). However, how ion channels achieved both incredible selectivity and conductance at the same time remained a mystery. In particular, this model could not answer the critical question of how K^+^-channels conduct K^+^ ions with a Pauling radius of 1.33 Å but exclude the very similar Na^+^ ion with a smaller Pauling radius of 0.95 Å. MacKinnon concluded, “the answer to this question would require knowing the atomic structure formed by the signature sequence amino acids” (264). The atomic structure of a bacterial homolog of the mammalian K^+^-channels, KcsA, indeed provided the answer just 3 years later in 1998 (47, 263). The SF of KcsA perfectly replaces the bound water molecules around a K^+^ ion entering the channel while the carbonyl oxygens of the SF are too far apart to correctly hydrate the Na^+^ ion.

Subsequent high-resolution structures of KcsA and Na^+^-channels confirmed this hypothesis and provided further molecular detail (265, 266, 267, 268). The structures of the SF of the K^+^-channels are composed of four backbone carbonyl groups from each subunit arranged into a long neck that perfectly matches the coordination configuration of a K^+^ ion in water (Fig. 8*B*). Dehydrated K^+^ ions in the filter are coordinated by eight oxygen atoms provided by the carbonyl groups, replacing the eight water molecules lost. This results in a minimum energy cost for a K^+^ ion to lose its bound waters in the solvent and to enter the SF. On the other hand, the preference of Na^+^ ion to bind to six water molecules in an octahedral configuration does not match the structure provided by the SF in the K^+^-channels. The energetic cost for Na^+^ to lose its bound water thus excluded it from entering the narrow pore. The long SF provides 4 K^+^ binding sites. High conductance is achieved through electrostatic repulsion between the positively charged K^+^ ions that pushes the ions through the filter. As a result, sites 1, 3 and 2, 4 are occupied by 2 K^+^ ions in an alternating fashion. Na^+^-channels on the other hand conduct Na^+^ ions in their partially dehydrated form with a wider and much shorter SF filled with water (Fig. 8*C*). The highly negatively charged filter and pore in Na^+^-channels would attract positively charged anions. Compared with K^+^ ions, the smaller Na^+^ ions would approach more closely this site of high field strength located at the extracellular mouth of the channel, with their preferred partial dehydration enabling entry into the SF and ion conductance pathway.

Activities of ion channels can be regulated in various ways (269). The nonequilibrium charge distribution due to the impermeability of the membrane itself results in an electric field across the membrane. As a result, regulation by voltage is one of the most common ways ion channels control their activities. Some channel proteins, such as the Na^+^-channels represented by the voltage-gated Na^+^-channel from *Arcobacter butzleri* (NavAb), can sense this electric field using charged residues embedded within the bilayer that move in response to changes in the magnitude or polarity of the field (269, 270). This conformational change can be coupled to dilation of the central pore for ions to diffuse through, as in the case of voltage-gated ion channels (VGIC). In some cases, enzymatic activity or other unexplored biological functions are used. All known so-called voltage-sensing domains (VSDs) are composed of four TMs arranged in a bundle (pink in Fig. 8, *D*–*F*), with at least one positively charged residue (magenta in Fig. 8, *E* and *F*) located within the bilayer. If a VSD contains multiple gating charges, these are typically arranged in an i, i+3n pattern along the helical path (Fig. 8, *E* and *F*).

In addition to gating charges that project into the four-helix bundle, VSDs often share a number of common structural and biophysical characteristics (Fig. 8, *E* and *F*). The charge-bearing gating helix (S4 in the Shaker cassette) often adopts a 3_10_ helix in order to stack the basic residues above one another. The four-helix bundle also contains a hydrophobic seal, also called the hydrophobic constriction site (HCS) or gasket (cyan in Fig. 8, *E* and *F*), which lowers the dielectric constant of the bundle and prevents leakage of ions and water through its center. Finally, negatively charged residues, denoted as the extracellular and intracellular negative clusters (ENC and INC), are often situated at either extreme of the VSD and can establish salt bridge interactions with the gating charges.

The conformational response of VSDs to changes in the trans-membrane electric field has been the subject of decades of biophysical and structural investigation. Armstrong and Bezanilla were the first to report “capacitive gating currents,” whereby the current elicited by gating charges moving across the membrane was directly observed (271, 272). These currents suggested that the gating charges likely move in a direction coincident with the transmembrane electric field vector. While the voltage-dependent dynamics of VSDs can be partially inferred by fluorescence measurements or chemical modification experiments (273, 274), direct visualization of these conformational transitions has not been possible simply because it is not possible to impose electrical gradients across the VGIC in either a lattice state suitable for X-ray diffraction or as a dispersion of single particles for cryo-EM. Therefore, the only states of the voltage-dependent specimen accessible to structural techniques are those that exist at 0 mV potential.

Early structures of voltage-gated K^+^-channels (K_V_ channels) at 0 mV potential suggested that the vertical motion of S4 couples directly to the S5 pore helix *via* a S4-S5 linker helix, which is situated in the plane of the membrane (275). As S4 moves upward, it tugs on the S4-S5 linker and in turn S5, causing the pore to dilate in a process known as electromechanical coupling (Fig. 8, *E* and *F*). Later structures of prokaryotic voltage-gated Na^+^-channels (Na_V_ channels) allowed for structural comparison of VSDs (266, 276, 277). While the overall structure of the VSDs was remarkably conserved, the observed orientations of the VSD with respect to the pore domain differed between channels. This was confirmed in the structures of two-pore channels where the VSD can move as a rigid body with respect to the pore domain, in addition to undergoing canonical charge transfer (79, 278). Unlike most VGICs that are activated at 0 mV, the two-pore channel 3 (TPC3) is only activated at extremely high voltage (V_50_ ∼ + 75 mV). The recently determined cryo-EM structure of TPC3 thus provides a unique opportunity to “see” a VGIC at resting state at 0 mV where the cryo-EM structure is captured (278). Consistent with the earlier hypothesis, S4 adapts a down position where only one out of the three arginine residues is above the HCS (Fig. 8*F*). In comparison, three out of the four arginine residues on S4 of NavAb are above the HCS, representing an activated state (Fig. 8*E*). Both structures confirm that S1–S3 also play critical roles in voltage sensing by providing residues in the ENC and INC that would stabilize the S4 in either the activated or resting state through salt bridge formation involving the arginines.

The ability to see the inner working of an ion channels by determining their atomic structures was critical for understanding how these amazing machines elegantly perform their functions that underly various essential biological processes. With more than 852 structures of ion channels in the PDB, we now understand how different ions selectively cross the membrane through specific ion channels and how their activities are regulated. As many ion channels have been implicated in various diseases, molecular understanding of their mechanisms and ready accessibility of their structures in the PDB are playing critical roles in the hope of treatments that target ion channels.

### How do vertebrate tissues prevent the passage of molecules through the spaces between neighboring cells? Claudins are molecular gatekeepers between cells.

In epithelium and endothelium, spaces exist between cells but molecules are restricted from unfettered transport. The restrictions imposed on these molecules force them to enter cells *via* transcellular transport mechanisms instead of passing between them. Vertebrate epithelium and endothelium regulate molecular movements between adjacent cells, paracellular transport, at narrow strands beneath the apical surface at specialized cellular contacts called tight junctions (TJs). As compartments within tissues require molecular exchange to maintain homeostasis, TJs assist here by forming membrane-associated multiprotein complexes to govern paracellular transport. The assembly of several families of MPs at TJs, specifically, creates either barriers or charge- and size-selective pores to small molecules and ions. The molecular properties of a given tissue or cell type, therefore, are determined by the MPs that assemble their TJs. As some molecules are only transported by paracellular means, regulation of this transport mechanism by TJs is critical to certain biological processes. Molecular transport through the paracellular space may have evolved in animal tissues as an energy conservation strategy, as a result of aerobic respiration. Impairing paracellular transport in mouse kidney results in normal ion absorption but increased oxygen consumption and hypoxia, demonstrating that the transcellular transport pathway alone is less energy efficient and that paracellular transport enhances kidney efficiency (279).

TJs were identified in 1963 by EM analyses of mammalian epithelial cells (280), but it was not until 1993 (281) and 1998 (282) when Tsukita and coworkers established the essential roles of integral MPs in TJ structure and function. Only one group of integral MPs, claudins (CLDNs), were capable of reconstituting TJ strands in TJ-less fibroblasts (283), distinguishing them as the structural backbone of TJs. Soon after, these were shown to be cell/cell adhesion molecules (284), which hinted at an ability to interact across paracellular space. The discovery of CLDNs led to newfound insights into the diverse functions of TJs and how these functions were enabled by CLDNs. These functions range from maintaining salt/water balance in fish gills (285), forming barriers in human skin to prevent dehydration (286), to sealing the blood–brain barrier—highly selective interfaces that provide a defense for the brain against pathogens and toxins in the blood (287). The plethora of TJ-linked diseases further demonstrates the diversity of TJ functions and how errors in TJ protein assembly contribute to these disorders, which include cancer (288, 289, 290), Alzheimer’s (291, 292), Parkinson’s (293) and Huntington’s (294) disease, amyotrophic lateral sclerosis (295), stroke (296), food poisoning (297) and irritable bowel disease (298, 299), hepatitis (300, 301), and diseases of the skin (302, 303, 304, 305), kidney (306, 307, 308), eyes (308, 309, 310), and ears (311, 312, 313).

Identification of the first two CLDNs led to subsequent classification of a CLDN family and the prediction of four TMs by cloning and sequence hydrophilicity analyses (314). In vertebrates, mammals have at least 24 subtypes, humans possess 27 (315), while pufferfish of the genus *Takifugu* have 56 claudins (316). Human CLDNs range in size from 23 to 34 kDa and are classified by a conserved WGLWCC motif. In addition to four TMs, most CLDNs contain a short N terminus and variable length C terminus and one intracellular and two extracellular segments (ECS). The two ECSs vary in length between subtypes, but ECS1 is larger and contains a conserved C54–C64 disulfide bridge. CLDN topology facilitates TJ assembly through: *i)* lateral CLDN/CLDN interactions within the same membrane (*cis*), *ii)* paracellular interactions with CLDNs on adjacent membranes (*trans*), and *iii)* cytoskeletal anchoring by soluble scaffolding proteins (317). *Trans* interactions between the ECS of adjacent CLDNs form paracellular barriers or pores to small molecules and ions. Because CLDN subtypes have specific permeability characteristics and tissue expression patterns, it is thought that the combinations and ratios of CLDN subtypes in given tissues’ TJs determine tissue barrier or pore properties. CLDNs are indeed classified by their barrier or pore-forming nature (318) and can be further delineated by levels of homology outside of the WGLWCC motif (319).

In April 2013, Suzuki *et al.* (320) uncovered the first ∼7 Å structure of a CLDN-like protein from the single-cell flagellate *Euglena* by electron crystallography. This protein, IP39, was predicted to have four TMs and a WGLWCC motif. Although the resolution of the IP39 structure obtained from 2D crystals was insufficient to determine the fold, the 3D EM density map revealed unique arrangements of IP39 in the lattice. IP39 molecules were packed in strands of antiparallel double rows, with trimeric units longitudinally polymerized, and one molecule of the trimer rotated 180° from the other two. These interactions were hypothesized to be important for linear polymerization of IP39 on *Euglena* membranes and potentially for TJ strand formation based on IP39’s sequence and topology similarity to CLDNs.

**Figure 9:**
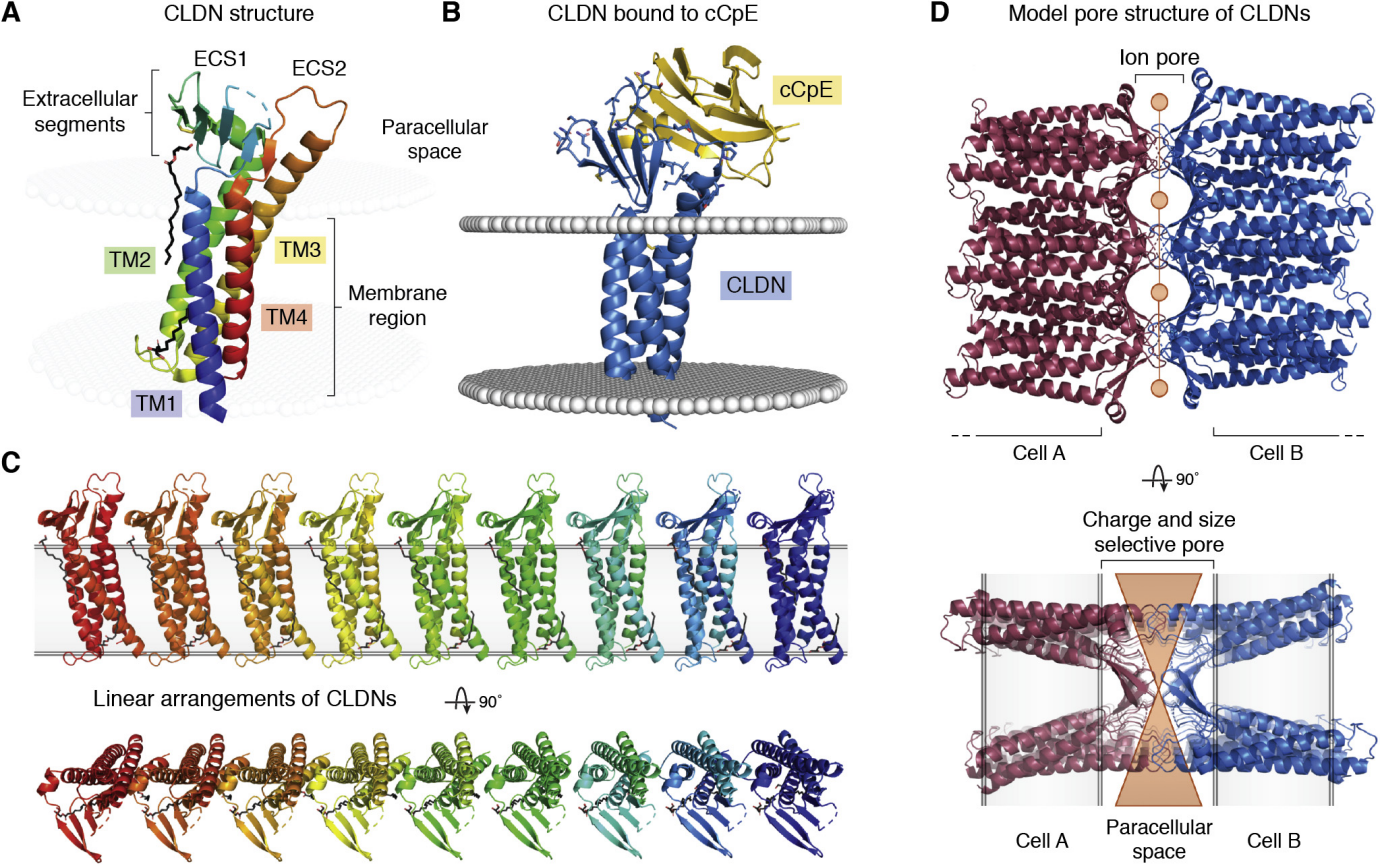
**Claudin structures and models for assembly at tight junctions.***A*, 3D structure of mCLDN-15 (PDB 4P79). *B*, 3D structure of hCLDN-9 in complex with cCpE (PDB 6OV2). *C*, the proposed linear arrangements of CLDNs as observed from the LCP crystal structure of mCLDN-15. *D*, structural model of CLDN-based paracellular ion pores.

One year later, Suzuki *et al.* (321) determined the first structure of a CLDN by X-ray diffraction at 2.4 Å resolution. Mouse CLDN-15 (mCLDN-15) is a cation-selective pore-forming CLDN (322, 323). To obtain 3D crystals of mCLDN-15, they employed a newer MP crystallization strategy, LCP (324), using a construct truncated of 33 amino acids at its C terminus and lacking four palmitoylation sites. The structure revealed the CLDN fold, which is composed of four TMs arranged in a left-handed bundle, one unstructured intracellular segment connecting TM 2 to 3, and two ECS linked together by a 5-stranded anti-parallel *β*-sheet (Fig. 9*A*). Unresolved in the structure was the loop connecting strand *β*1 to *β*2 and the N terminus, while two monooleins were resolved and located near F11 and W49. The crystal structure suggested a potential pathway for paracellular transport by revealing that a negatively charged electrostatic surface exists in the C-terminal half of ECS1, specifically the area between *β*3 and *β*4. As mutations to residues in this area were known to reverse ion charge selectivity (325), the structure explained how CLDNs may govern ion selectivity in TJs. In addition, like IP39, the mCLDN-15 packing within the lipid-filled crystal lattice suggested how CLDNs may polymerize. It was hypothesized that lateral *cis* assembly of CLDN-based TJ strands *in vivo* resembles the linear arrangements found *in crystallo* between certain symmetry mates. These packing arrangements would be used as a basis for modeling of larger TJ polymer strands and paracellular pores.

Throughout animal evolution, CLDNs and the paracellular barrier have likely been subjected to many attempts at disruption by pathogenic organisms seeking entry into epithelial tissues. The spore-forming Gram-positive bacterium *Clostridium perfringens*, for example, secretes a 35 kDa enterotoxin (CpE) within the gastrointestinal tracts of humans and domesticated animals that binds select CLDN subtypes through recognition by its C-terminal domain (cCpE) (326). The CLDN ECS2 alone was thought to be used for this recognition (327, 328). However, structure determination of CLDNs in complex with cCpE has revealed that cCpE binding encompasses both ECSs (Fig. 9*B*). The cCpE has proven to be an important tool for structural determination of CLDNs because the toxin fragment adds a ∼15 kDa hydrophilic mass to hydrophobic CLDN/detergent complexes, which allows for formation of 3D crystals *via* traditional vapor diffusion methods. The cCpE has enabled structural determination of mCLDN-19 (329), human CLDN-4 (hCLDN-4) (330), mCLDN-3 (331), and hCLDN-9 (332). To date, 89% of CLDN structures in the PDB are CLDN/cCpE complexes, highlighting cCpE’s value to CLDN structural biology.

In addition to enabling structural determination of several CLDNs that were recalcitrant to crystallization alone, cCpE-bound CLDN structures facilitated our understanding of both fundamental and clinical aspects of TJ biology. CpE binding to CLDNs is thought to disable both lateral and paracellular CLDN/CLDN interactions, inhibiting paracellular pore and cell adhesion functions (329, 330, 331, 332). Crystal structures of CLDN/cCpE complexes explain how cCpE may disable both types of CLDN/CLDN interactions (Fig. 9*B*). By encompassing both ECS, cCpE can prevent CLDN/CLDN *trans* interactions through steric shielding. The cCpE can also disable lateral CLDN/CLDN interactions through cCpE-induced structural perturbations of two extracellular helices shown to be involved in *cis* assembly (329, 330, 331, 332). In drug development and in the clinic, the intermolecular interactions between cCpE and CLDNs revealed by these structures are empowering efforts to engineer cCpE for targeted destruction of CLDN-expressing cancer cells (333, 334, 335) and for improving drug delivery through the blood–brain barrier (336, 337).

Structures of CLDNs have allowed for the modeling of the other subtypes and their functional pore-forming assemblies using *in silico* methods. The linear polymers of mCLDN-15 observed in LCP-grown crystals, packed in crystallographic symmetry arrangements, form potential *cis* interactions using adjacent ECS domains (321) (Fig. 9*C*). Building complexity from this experimentally determined organization, a more complex model was proposed where a row of linear polymers could interact with a second row of linear polymers to arrange in antiparallel double rows, with the ECS of neighboring protomers creating *β*-barrel-like channels in paracellular space (338) (Fig. 9*D*). Since, researchers have computationally modeled an array of possibilities for how mCLDN-15 (339, 340, 341) and other CLDNs (342, 343, 344) may assemble into large complexes essential for paracellular barrier and pore function. These predictions have provided testable models of CLDN self-organization, pore structure and selectivity, and the *cis* and/or *trans* interfaces that enable these assemblies and functions. Although some models or portions of them have been verified *in vitro* and/or *in vivo* using cross-linking, mutagenesis, freeze-fracture EM, fluorescence confocal microscopy, and FRET (338, 341, 342), an experimentally derived structural basis for CLDN interactions and assemblies has yet to be elucidated.

Recent progress in determining structures of CLDNs has rapidly advanced our understanding of the interconnected structural and functional biology’s of CLDNs and TJs and the mechanisms by which a bacterial enterotoxin gains access to the mammalian gut. These advances are somewhat limited by a lack of structural information for the multimolecular CLDN assemblies required to form TJ barriers and pores and CLDN complexes with other TJ-constructing proteins. The dynamic associations between soluble proteins and MPs that properly assemble TJs under various physiological stimuli, which are thought to contain >40 proteins (345), make for a potentially arduous yet exciting future for the field. It is expected that structural biology will make critical contributions to deconvolute and demystify how TJs form and function at the subnanometer levels. Such discoveries will provide a rich source of information for making new inroads in the design and development of therapies to treat the plethora of TJ-linked diseases—like cCpE-based therapeutics targeting cancer and neurological disorders—effective, possibly, at the level of specific tissues, cell clusters, or even individual TJs.

### How do solutes cross the membrane?

One of the important categories of MPs are transporters for solutes to cross the lipid membrane (346). These solutes either become part of a metabolic cycle (*e.g.*, glucose transporters) and generate energy for the cell, or these solutes can act as signaling molecules (*e.g.*, Ca^2+^ ATPase that transfers Ca^2+^ ions) to activate various biological processes (347). Active transporters, which utilize energy to transport substrates, are classified into primary and secondary active transporters based on the energy source (348). Primary active transporters such as P-type ATPases (*e.g.*, Na^+^/K^+^-ATPases, Ca^2+^-ATPase, gastric H^+^/K^+^-ATPase, fungal and plant H^+^-ATPases), V-type (vacuolar-ATPase), and ABC transporters depend on ATP hydrolysis to fuel the transport of solute across the hydrophobic lipid membrane (349). Secondary transporters transport solutes against their gradients by using the potential energy from downhill electrochemical gradient of another solute (350). We describe the impact of the PDB in the field of one of the primary transporter classes, the ABC transporters (351). For secondary active transporters, we focus on the major facilitator superfamily (352).

#### ATP-binding cassette transporters

ABC transporters are one of the largest superfamilies of MPs, ubiquitously present in all prokaryotes and eukaryotes (353). They are involved in major physiological functions by transporting diverse types of solutes including sugars, ions, drugs, lipids, bile salts, amino acids, peptides, nucleotides across membranes (354). Mutations are associated with a number of diseases including cystic fibrosis, Tangier’s disease, Dubin–Johnson syndrome, and also account for multidrug resistance in human cancers (355). ABC transporters are classified as importers or exporters based on the direction of substrate transport relative to the cytosol (356). According to the new classification, based on different structural folds, ABC transporters are divided into type I to VII classes (357). These transporters consist of two transmembrane domains (TMDs) and two nucleotide-binding domains (NBDs). NBDs bind and hydrolyze ATP to power the translocation of substrate across the membrane through the transmembrane domains (358, 359). Some ABC importers also use accessory proteins to bind the substrate with high affinity and then transport it across the membrane (360). Structure entries since the 1990s demonstrate how the PDB remarkably impacted the field of ABC transporters (361).

**Figure 10:**
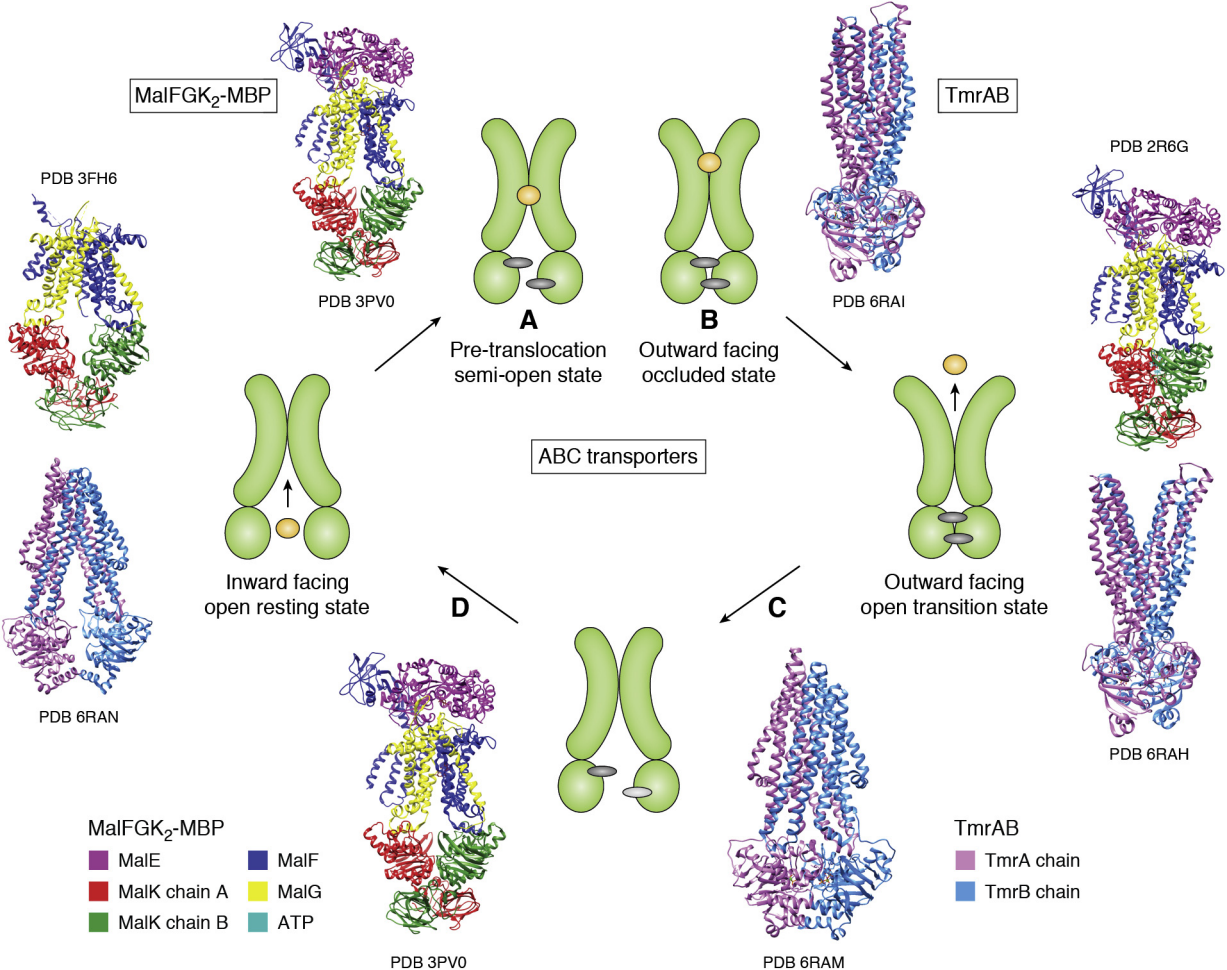
**The alternating access mechanism in ABC transporters deciphered by structural tools.** For simplicity, the cycle direction describes an exporter. Importers function in the opposite direction of the *arrows*. An ABC transporter in its inward-facing open resting state (PDB 3FH6 for MalFGK_2_, PDB 6RAN for TmrAB) binds to ATP in the presence (in the case of maltose transporter) or absence of substrate [A] to adopt an intermediate state (semiopen pretranslocation state (PDB 3PV0) in the case of the maltose transporter, or an outward open occluded state in case of TmrAB (PDB 6RAI)). NBDs dimerize for ATP hydrolysis in an outward-open transition state (PDB 2R6G for the maltose transporter, PDB 6RAH for TmrAB) [B]. ATP hydrolysis separates NBD domains, and the ABC transporter adopts an inward open post hydrolysis state (semiopen state for maltose transporter (PDB 3PV0), inward-facing unlocked state for TmrAB (PDB 6RAM)) [C], which resets the resting state [D]. To simplify the figure, the binding and release of substrate are shown only for the case of exporters. Substrate is effluxed outside at step [B] in the case of the exporters. In importers, the substrate is released inside at step [C and D]. Color coding: MBP (MalE) = *magenta*, MalK chain A = *red*, MalK chain B = *green*, MalF = *blue*, MalG = *yellow*, ATP = *cyan*, maltose = *brown*. TmrA chain = *pink*, TmrB chain = *sky blue*.

Structures of ABC transporters not only provided insights into the architecture of ABC transporters, but also contributed toward understanding the alternating access mechanism in the family. The *Escherichia coli* maltose transporter or MalFGK_2_, a well-characterized model system of the ABC transporter superfamily (362, 363), imports maltose or maltodextrins with lengths of up to 7 to 8 linked glucose units in coordination with cell metabolism (364). Crystallographic snapshots of the maltose transporter in different states demonstrate how it utilizes an alternating access mechanism to transport maltose from the periplasmic side to the cytosol using a periplasmic maltose-binding protein (MBP, also named MalE) to feed the transporter from the periplasm and ATP hydrolysis on the cytoplasmic NBD domains (365). These studies revealed that in the absence of MBP and nucleotide, the NBDs of maltose transporter are well separated and do not hydrolyze ATP; a state known as inward-open resting state (366). Binding of maltose-binding protein charged with maltose and ATP binding to the NBDs induces a conformational change that brings the transporter into a pretranslocation semiopen state in which the NBDs are not fully closed (Fig. 10*A*) (367). In the next step, it undergoes a concerted motion in which substrate is released into the intracellular cavity and NBDs dimerize or close completely to initiate ATP hydrolysis; a state known as the outward-open transition state (Fig. 10*B*) (368). ATP hydrolysis returns the maltose transporter to the resting state through an intermediate posthydrolysis semiopen state and releases the substrate to the intracellular side (Fig. 10, *C* and *D*) (365).

In addition to elucidating the catalytic cycle of the maltose transporter, structural biology of this transporter also explained the biphasic behavior of its MBP-independent mutants such as MalG511 (366) and informed about substrate specificity toward malto-oligosaccharides (369). MBP- independent mutants had high basal activity and showed an interesting biphasic behavior in maltose transport assays, as well as in ATPase assays in response to increasing equimolar concentrations of MBP and maltose (370, 371, 372). Equimolar concentrations of MBP and maltose had an activating effect at low concentrations, but inhibitory effects at higher concentrations on these mutants (371, 372). These MBP-independent mutants were hypothesized to resemble the transition state of the maltose transporter, with lowered activation energy and increased affinity toward MBP (372, 373). The mutated residues in these mutants are located at regions near a periplasmic gate involved in transition from the inward-open to the outward-open state (366, 368, 374). Mutations in these regions destabilize the maltose transporter in the resting state and shift the equilibrium toward a transition state, which explains the high basal activity of these mutants in the absence of MBP (366, 372). Higher affinity of MBP for the transition state *versus* the resting state explained the biphasic behavior of these mutants (372).

Along similar lines, TmrAB, a thermophilic bacterial homolog of the mammalian antigen-presenting ABC transporter TAP1/TAP2 (375), was one of the first ABC transporters with pseudo-twofold symmetry, whose subnanometer resolution structure was determined by Fab-assisted single particle cryo-EM (376). In a remarkable tour de force, crystallographic studies combined with cryo-EM studies determined the conformational landscape of TmrAB in lipidic environment during turnover conditions (375, 377). These studies demonstrated the inward-open and outward-open states as a part of general alternate access mechanism and showed induced conformational changes upon ATP binding and hydrolysis, coupled with substrate movement across the membrane (Fig. 10) (377). Advances in cryo-EM technology have enabled deciphering the alternating access mechanism in individual ABC transporters including the chloride transporter CFTR (378, 379, 380, 381, 382), MRP1 (383, 384, 385), P-glycoprotein (386, 387, 388), and BCRP (389, 390, 391, 392).

The PDB facilitated further understanding of the catalytic cycle of ABC transporters. As an example, the crystal structures (PDB 2R6G) of the maltose transporter (368) enabled detailed dynamics studies using EPR (393, 394). The dynamics of the maltose transporter determined by EPR complemented the crystallographic structures representing the catalytic cycle (395, 396, 397). Other ABC transporters including MsbA, LmrA, ModBC_2_, BtuCD, P-glycoprotein, and TmrAB were also probed by EPR spectroscopy to understand similarities in catalytic cycles and the differences that define unique features of each ABC transporter (398).

#### Major facilitator superfamily (MFS) transporters

The MFS transporters are the largest family of secondary transporters in which individual members translocate one of a diverse range of particular substrates. The substrates include sugars, ions, amino acids, and diverse metabolites (399, 400). This superfamily is ubiquitous, found in all kingdoms of life, and in all cells, and in humans many members are of direct medical and pharmaceutical significance.

**Figure 11:**
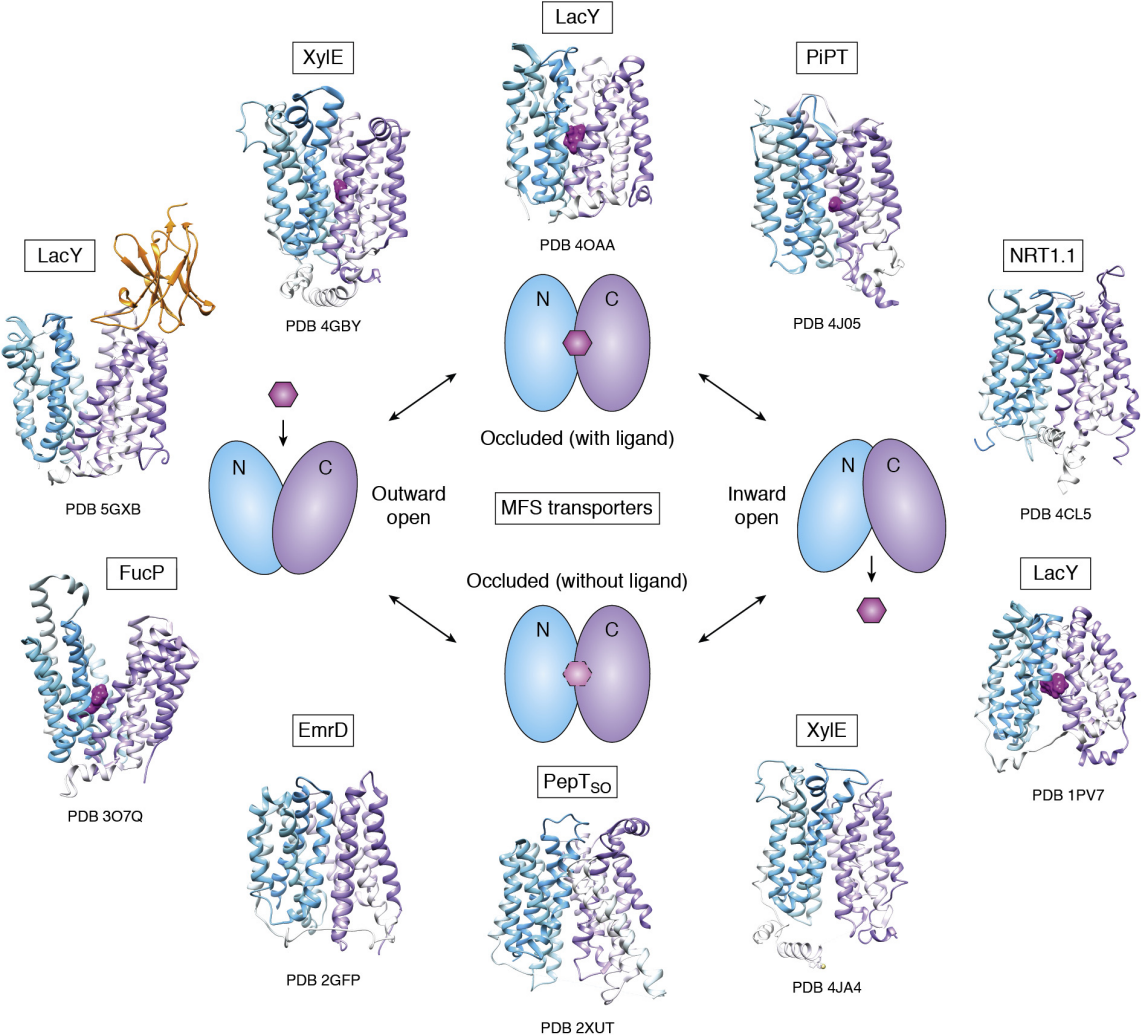
**Distinct conformations of major facilitator superfamily transporters.** Representative structures for the distinct conformations in the substrate transport cycle are shown. The cytoplasmic side of the membrane is “down” and the outside of the cell upward in this figure. In the text the cycle is described as if on a clock face. The structures of several MFS transporters are shown in their relative positions around the “clock.” A key point is that there is no leakage in these transporters. The 9 to 12 to 3 and in reverse (clockface numbers) indicate exchange 1:1 of substrate. The 3 to 6 to 9 and back to 6 to 3 indicate the substrate-free transfer, which in the case of LacY is the critical part that then delivers the proton.

The key aspect is that they alternately bind or release substrate on one side of the membrane and bind or release that one molecule on the other side, termed alternate access. If the cycle in Figure 11 is described as if a clock face, this is schematized in the 9 to 12 to 3 o'clock parts of Figure 11 (and vice versa). This process can be proved using radioactive substrate on one side of the membrane that appears on the other side of the membrane, even against a concentration gradient of total substrate in the opposite direction. It occurs between opposite sides of a membrane-enclosed volume without any ion gradient across the membrane. So-called “uniporters” do this without coupling to any other ion or proton gradient and so simply allow equilibration of substrate from one side to the other to achieve equilibrium where both sides eventually reach equal concentration. The glucose transporter GLUT1 falls into this category. In biology the metabolism of glucose inside the cell reduces cytoplasmic glucose inside and thus ensures that GLUT1 actually leads to net import of glucose.

In the vast majority of MFS transporters, transport is obligately coupled to an electrochemical proton or Na^+^ gradient. The gradient drives the transport of substrate against their substrate concentration gradient, using the downhill free energy change of one process (*i.e.,* the electrochemical ion gradient) to drive the uphill process of the other (*i.e.,* substrates). In transporters the movement of ion and substrate may be in the same direction, termed symporters, or in the opposite direction of the substrate transport, termed antiporters, and the processes are reversible, determined by the relative concentration gradients of the driving ion and of the substrate. In a closed system, they would reach a two-substrate equilibrium. In cell biology though, the gradients of the driving ions are continuously being generated by ATP-driven pumps that then determine the direction of transporters that use those gradients (352, 401). The PDB harbors ∼150 structure entries of full-length or truncated MFS transporters bound to substrates and small molecules determined by various methods, most by X-ray crystallography with a few by cryo-EM. These structures each outline different states around a transport cycle (Fig. 11) that shows how small are the energy barriers between different states around the Figure 11 clockface.

Mutations and misregulation of human MFS transporters contribute to human diseases. GLUTs (glucose uniporter family) are linked to various genetic and metabolic diseases. Essential for maintaining glucose homeostasis their loss of function or regulation can underlie type-2-diabetes (402). GLUT1 is also overexpressed in many cancer types due to the increased demand for glucose that plays a key role in tumor growth (403, 404, 405). The substrates of GLUTs are not restricted to glucose as some of the homologs transport urate or other sugars broadening their contribution to human physiology. As an example, GLUT9 transports urate and is implicated in hyperuricemia and gout (406, 407). Other human MFS transporters implicated in disease include human PEPT1 and PHT1. They are involved in transport of small peptides, and their misregulation can cause inflammatory bowel disease (IBD) (408). Organic anion and cation transporters transport large hydrophobic ions including neurotransmitters, hormones, various drugs and are associated with coronary heart disease, IBD, prostate cancer, rheumatoid arthritis, and other conditions (80, 409, 410, 411). Given the role of MFS transporters in human health and disease, their structures are an important driver for drug design.

The structures of MFS transporters shed light on their fundamental mechanism (352, 400, 412). They have a single central substrate-binding site and operate *via* an alternating-access mechanism that involves a rocker-switch type movement of the two halves of the protein (Fig. 11). The 12 TMs are arranged into two six-helix bundle domains (TM1-6 and TM7-12) and the motion of the N- and C-terminal domains results in alternating access, with the substrate-binding site as a pivot point. Structures guide the notion of a shared MFS fold, irrespective of their particular function as symporter, uniporter, or antiporter, or substrate (Fig. 11). Salt-bridge formation and breakage are involved in the conformational changes of the protein during transport (352, 401). The structures of MFS transporters suggest that there is more to the mechanism of substrate translocation than just rigid-body movement of the two transmembrane halves. A more compact arrangement of helices around the bound substrate is found in some structures (413, 414, 415). Homology modeling, MD simulations, and double electron–electron resonance measurements suggest a similarly compact structure for the substrate-bound lactose permease (LacY) (416, 417, 418). A comprehensive analysis of MFS transporter structures by Quistgaard *et al.* (401) revealed a possible gating mechanism for substrate transport. Therefore, the atomic resolution structures of these transporters describe the unique aspects of their functions and provide an overarching view of an underlying mechanism (Fig. 11).

Structural information helps classify MFS proteins into subfamilies (352, 400) as some proteins with no detectable sequence similarity may exhibit almost identical structural arrangements. Mutants designed using biochemical and biophysical analysis of MFS transporters help stabilize otherwise unachievable conformations within the transport cycle (419, 420).

The lactose permease LacY of *E. coli* is one of the best understood MFS transporters (421). It couples the proton electrochemical gradient from outside to inside across the plasma membrane of a bacterial cell to cotranslocation of a galactopyranoside against its concentration gradient, into the cell (422). The stability-conferring C154G mutant (in TM5) binds substrate in the same manner as the WT LacY but with negligible transport (423). This conformationally restricted mutant led to the first X-ray crystal structures of LacY, perhaps because it was locked into position by the mutation (424, 425). Later structures showed how LacY binds substrates without this mutation (426) and related the difference in binding of substrate *versus* water in the binding site to changes in the environment of a nearby arrangement of titratable side chains including E325, H322, K319, Y236, D240 that serve to capture and release protons (412). Surprisingly the binding (or unbinding) of substrate (the 9 through 12–3 position in Fig. 11) did not change the protonation state of the protein as measured by any release of protons upon binding substrate. And the *Kd* for binding substrate remains unchanged up to pH 10.5 (427). So where does the coupled movement of the driving H^+^ ion, that is, protonation and deprotonation, occur to drive the process? Proton delivery occurs only if the protein goes through alternate access *without* the substrate bound (6 o'clock in Fig. 11). The H^+^ ions get “squeezed out” from the side that closes (3–6 or 9–6) to reach the occluded state (6 in Fig. 11), but only when water rather than substrate fills the substrate site (6 in Fig. 11). Effectively the active substrate-binding site loses protons as water retunes the substrate site. Once on the “other side” of the membrane, the site rebinds protons from that “other side” (6–9, or 6–3 in Fig. 11) as the *pK*a again quickly rises from around p*K*a of ∼5 in the water-occluded state (6 in Fig. 11), to 10.5 when it opens to the other side (Fig. 11). The process is driven by a sharp drop in the *pK*a of the titratable substrate proximal region associated with glutamate E325 when water is in the site, *versus* when there is substrate in the site, *i.e.*, the loss of a proton from that conjugate site (428, 429). As the transporter closes, for example, from the cytoplasmic side, if no substrate is bound, the *pK*a of E325 system is lowered to release protons to the closing side, and those protons are rapidly moved away *via* water molecules.

In an effort to visualize the alternate access mechanism, two conserved Gly-Gly pairs (G46/G370 and G159/G262) in LacY were mutated to tryptophans to pry the alternate sides open. The mutants modified the structure and were investigated using transport and stopped-flow binding measurements (430, 431). The double Trp mutant G46W/G262W locked the LacY conformation into the outward (periplasmic)-open conformation and led to structures that defined interactions in the outward-facing state. A camelid-nanobody against the G46W/G262W double mutant was screened against WT LacY and selected for the locked conformation similar to the double mutant (430, 432) beautifully validating these structures as reflecting on pathway states of the “rocker switch” (419, 420) (Fig. 11).

The sugar-transporting MFS transporter XylE (433), a bacterial hGLUT1 ortholog, was one of the first MFS transporters for which the structures of multiple conformations were deciphered. XylE structures in complex with *D*-xylose and *D*-glucose led to the structural understanding of its specificity and provided the basis for subsequent mechanistic understanding of hGLUT1 specificity (see How does understanding of a glucose transporter selectivity and conformational changes inform the development of GLUT1-specific inhibitors?) (403, 434). A mixture of detergents and substrate during crystallization led to an inward-open conformation of XylE (435). Inspired by the double Trp mutant of LacY, a similar mutant designed for XylE (G58W/L315W) gave an outward-facing conformation (433).

Several general conclusions from MFS transporter structures ensue. Substrate binding, *versus* absence of substrate binding, changes the environment of the titratable/ion-binding sites nearby the substrate so that the affinities for proton/ion are altered. In LacY, the “active site” p*Ka* drops (releases a proton(s)) only when LacY returns to the other side empty of substrate. This mechanism beautifully explains how an intrinsically reversible transport function couples substrate delivery to one side (opening on that side), to “squeezing out” the proton(s) on that same side, when recycling empty of substrate to be available for another substrate to be bound on the other side. There is no exact stoichiometry of proton/ion to substrate, as determined by the p*K*a values and may involve several H^+^ in this case, or Na^+^ ions in eukaryotes. Thus the driving proton(s)/ion(s) and substrate transport are coupled but differently in space and in time (412).

Other superfamilies also work by alternate access. As an example, several other Na^+^-driven transporters from different gene families share a common fold with LeuT (see How do drugs control our minds? Molecular insights for psychiatric drugs) for the central core of ten TMs and seem to share a common mechanism for coupling to Na^+^ even though they are not conserved in sequence with LeuT itself. These include the clinically important Na^+^-driven glucose transporters of the SGLT family (436, 437, 438, 439, 440). Together these structures show how their structures, rather than their sequences, identify common mechanisms of transport.

### How small viral transmembrane proteins could be a linchpin of viral infection?

**Figure 12:**
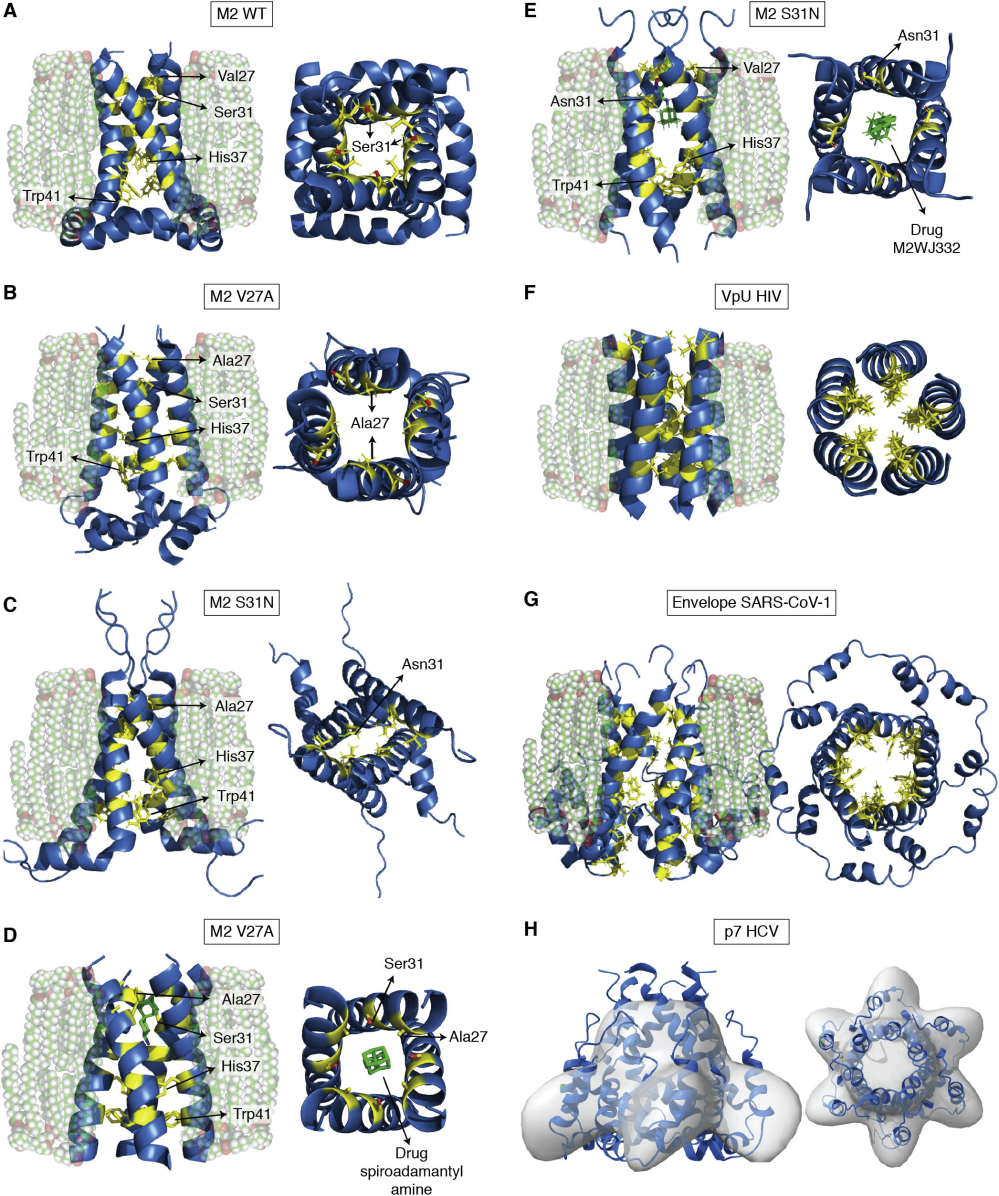
**Viroporin structures.***A*, M2 PDB 2L0J (wild-type). Pore residues V27, S31, H37, and W41 highlighted in *yellow*. *B*, M2 PDB 2KWX (V27A mutation). Pore residues A27, S31, H37, and W41 highlighted in *yellow*. *C*, M2 PDB 2N70 (S31N mutation). Pore residues V27, N31, H37, and W41 highlighted. *D*, M2 V27A mutant (PDB 6NV1) in the presence of the spiroadamantyl amine inhibitor. Drug in *green* and pore residues A27, S31, H37, and W41 in *yellow*. *E*, M2 S31N mutant (PDB 2LY0) in the presence of the drug M2WJ332. Drug in *green* and pore residues V27, N31, H37, and W41 in *yellow*. The mutants V27A and S31N are both resistant to amantadine, the S31N mutant is the most common and now present in most Influenza A infections. The structures in panels *C* and *E* differ by the length of the construct, the absence and presence of drug, and the environment in which the structures were solved. While the M2 S31N structure in panel *C* was solved in liquid crystalline lipids environment, the structure in panel *E* was solved in a micellar environment. *F*, VpU TM pentameric structure (PDB pi7) *side* and *top view* showing in *yellow* the residues V9, V13, I17, and V21 lining the channel pore. *G*, structure of SARS-Cov1 Envelope protein TM and C-terminal domain (PDB 5X29) with pore lining residues N15, L18, A22, F26, V29, and I33 highlighted in *yellow*. *H*, Hepatitis C Virus p7 protein structures PDB 2M6X (pentamer) and PDB 2MTS (monomer) fitted to the cryo-EM density map (emd_1661) using *ChimeraX*.

Now more than ever there is an increased interest in the study of viral proteins. The focus has been rightfully placed on replicases, polymerases, and receptors that enable viruses to pursue their dichotomic life cycle transitioning from a “lifeless” state as a viral particle to a prolific replicator when hijacking the host-cell machinery. In the particular case of membrane-enveloped viruses, the viral proteins essential to the ingress of the virus into the host cells are MPs that are important drug targets, as demonstrated with the spike protein from Severe Acute Respiratory Syndrome Coronavirus 2 (SARS-Cov2) (441, 442, 443). However, there is a class of membrane-embedded proteins termed VPs that are essential for virulence and infectivity (444, 445, 446) and have proven to be a valuable but yet elusive targets for therapies. VPs are small transmembrane proteins (60–120 amino acids) that oligomerize into ion channels capable of permeabilizing cell membranes (447). They are classified into class I for single pass TMs and class II for helix-turn-helix hairpin motifs (445, 447). Among the most studied VPs are the M2 proteins from Influenza A (IA) (448, 449, 450, 451, 452, 453, 454, 455) and Influenza B (456, 457), VpU from HIV (458, 459, 460, 461, 462, 463, 464, 465, 466, 467), the Envelope protein from Coronaviridae (468, 469, 470, 471), and the p7 protein from Hepatitis C virus (472, 473, 474, 475, 476) (Fig. 12). Although all of the above have structures of either the full-length protein or of the TM monomer deposited into the PDB, the oligomerization state and ion conductance mechanism of some of these ion channels remain elusive given that the channel pore lumen is often lined by hydrophobic amino acids (*e.g.*, VpU and Envelope protein SARS-Cov1 (467, 471)). Moreover, VPs play diverse roles during the viral life cycle, modulating different host cell functions by directly interacting with host proteins (445, 458) or, as in the case of Influenza A VP M2, effecting the membrane curvature associated with the maturation and release of viral particles (451).

The M2 proton channel of IA is a canonical VP with more than 30 structures determined over the years informing many biochemistry studies. This tetrameric ion channel is essential for the IA infection. Upon acidification of the endosome by the cell machinery, M2 is activated to shuttle protons inside the viral particle, triggering disassembly of the viral core protein M1 and subsequent release of the ribonuclear protein complex (477). The proton shuttling process from the virus exterior to its interior by VP M2 is dependent on the protonation of residue H37 (448, 450, 478, 479, 480). Another major player involved in the proton conduction is W41, which, unlike other MPs where the aromatic residues are located with polar moieties oriented out from the membrane surface to act as anchor, faces the interior of the pore lumen to function as the channel’s primary gate (481).The S31N mutation in M2 (Fig. 12*C*), first identified in 2006 and now prevalent in all strains of the virus, has rendered M2 IA resistant to amantadine and rimantadine, antivirals widely used for the treatment of flu (482). The functional core of this channel is comprised of a tetrameric helix bundle spanning the membrane and a juxtamembrane amphipathic helix that anchors the channel to the membrane and is essential for the process of nascent viral particle release from the host cell membrane (451) by inducing negative curvature around the nascent virion budding neck (483). The available structures of M2 IA have shown that the mechanism by which amantadine and rimantadine block ion conductance is by physically plugging the ion channel pore with their cage-like bulky adamantane chemical moiety. The S31N mutation seems to prevent such inhibition by placing a bulky Asn side chain in the pore entrance while the V27A mutant accomplishes this by removing the bulky Val side chain from the pore lumen that secures the inhibitor in place (Fig. 12, *B* and *C*). Many structures of the S31N mutant have been determined with the goal of understanding the mechanism by which this mutation blocks the entrance of a bulky ligand such as amantadine without affecting ion conduction (454, 484, 485). Recent studies, built on previous work by screening adamantane-derived compounds as well as non-adamantane compounds (485, 486, 487, 488, 489, 490, 491, 492) against the S31N mutant, have identified inhibitors blocking the channel *via* the same mechanism used by amantadine and rimantadine *i.e.*, obstructing the channel pore (Fig. 12*E*).

## Membrane protein structures instruct drug design and protein engineering

More than 50% of approved drugs target GPCRs and ion channels. Many drugs are metabolized by membrane-associated cytochrome P450s and/or exported by members of the solute carrier family (SLC) or ABC transporters involved in absorption, distribution, metabolism, and excretion of drugs (493, 494, 495, 496). The democratization of protein structures since the establishment of the PDB has significantly contributed to the development of new therapies and the understanding of their mode of actions. The availability of MP atomic coordinates that can be accessed free of charge, viewed and manipulated on any researcher’s computer opens endless possibilities such as *i)* designing mutagenesis studies to understand the roles of different residues in protein structure, function, and disease, *ii)* guiding rational protein engineering and design efforts aimed at improving the biophysical properties of protein targets to facilitate drug discovery and *iii)* modeling of putative binding sites. Structure-based drug development is one major area where PDB continues to foster and benefit the drug development process and ultimately leads to the more effective treatments for patients.

Protein engineering allows use of proteins for new applications including many advances in structural biology. Without the various purification tags/fusions we now use commonly, the PDB would still be limited to proteins from natural sources (497). Protein engineering also had significant contributions to the structural determination of many MPs themselves. The flourishing field of GPCR structural biology would not have been possible without the use of fusion proteins (such as modified T4 lysozyme) as crystallization chaperones (498). With increased knowledge of MPs, they can be used as powerful tools in many biochemical and biophysical applications. However, they need to be modified and optimized for applications under experimental conditions. The availability of structures of MPs is critical for optimizing properties of interest.

In this section, these contributions of the PDB to understanding MPs in the context of therapy and the new ways of understanding biological processes enabled by structure-based protein engineering of MPs will be explored through a few illustrative examples.

### How do structures of the membrane-bound cytochrome P450 enzymes inform the functional basis of monooxygenase activities, contribute to understanding of drug–drug interactions, and aid drug discovery?

Cytochrome P450 enzymes are monooxygenases with a highly conserved and unique fold (499). They have evolved to carry out specific metabolic reactions that help produce and modify metabolites including sterols, some lipids, and vitamins, or they can act as more broadly specific activators or detoxifiers of drugs. Despite this diversity of function and substrate clientele, all cytochrome P450s have the ability to generate, in a buried substrate specificity-conferring active site, an Fe(IV)-oxo-porphyrin cation radical (compound 1) that cleaves aliphatic carbon–hydrogen bonds by introducing an oxygen and producing water (500). When the PDB Molecule of the Month provided a succinct summary of current knowledge of the structure, function, and mechanisms of cytochrome P450s in 2006 (https://pdb101.rscb.org/motm/82), there were about 13,000 DNA sequences for cytochrome P450s described in databases and the PDB was dominated by crystal structures of microbial cytochrome P450s. The cytochrome P450s of higher eukaryotes had just begun to assume prominence in the PDB with the lodging of crystal structures of the catalytic domain of some membrane-bound drug metabolizing liver enzymes, such as cytochrome 3A4 in complex with various ligands (501). Now there are at least 600,000 cytochrome P450 DNA sequences and about 7000 NMR and crystal structures in the PDB for these molecules, primarily due to genome sequencing, the use of heterologous expression systems, and the exploration of substrate and ligand binding by academia and industry. This is enabling efforts to design more specific ligands for pharmaceutical and agricultural purposes (502, 503, 504), the use of mutagenesis and metal substitution to engineer new activities (505), and experiments that increase understanding of mechanistic features of cytochrome P450s.

Membrane-bound cytochrome P450s, which locate to the endoplasmic reticulum or mitochondria of eukaryotic cells, have catalytic domains with a comparable fold but were classified as significantly structurally different from the soluble P450s that occur in bacteria (506). This finding was based primarily on the interaction of the heme ring C propionate with the helices A–B loop in the case of the membrane-bound enzymes and with helix C for the soluble enzymes. The membrane-bound CYP51 enzymes provide an illustrative exception to this generalization. Both soluble and membrane-bound enzymes in this phylogenetically ancient cytochrome P450 family have their heme C propionate in an ionic interaction with a basic residue in helix C. A second factor used to discriminate between soluble and membrane-bound cytochrome P450s was the increased length and more complex disposition of the F–G helix region in the membrane-bound cytochrome P450s.

**Figure 13:**
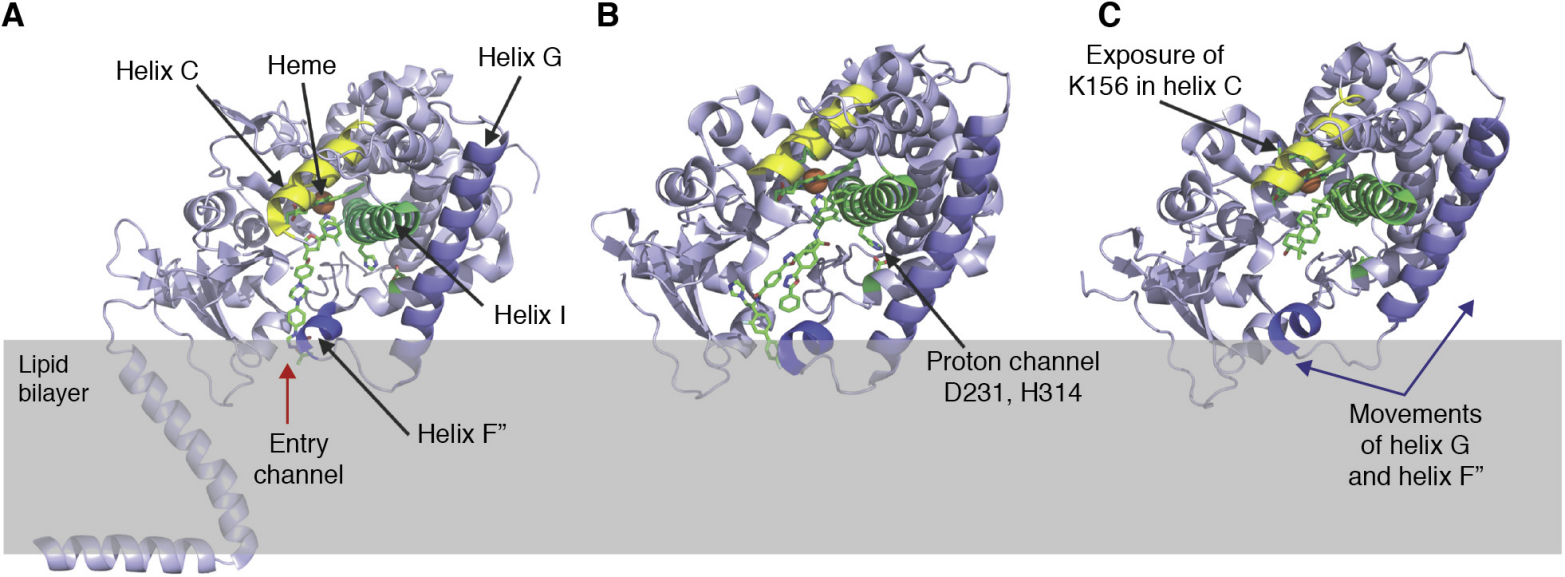
**Comparison of the full-length *Saccharomyces cerevisiae* CYP51 structure with structures of the human CYP51 catalytic domain.** Helix B and the B–C loop have been removed to visualize the interior of these molecules and their ligands. The catalytic domain of the full-length yeast CYP51 enzyme (*A*) in complex with posaconazole and the human catalytic domain in complex with two molecules of the inhibitor VFV (*B*) have essentially identical folds, with their helix C K151 and K156 sidechains, respectively, making ionic interactions with the heme propionate. Ablation of the proton transfer pathway in the human CYP51 D231/H314 mutant catalytic domain (*C*) gives substrate occupancy sufficient to visualize lanosterol bound in an inactive but catalytically competent state that demonstrates significant conformational change in comparison with *B*. K156 no longer binds to the heme propionate—its side chain projects above the enzyme’s proximal surface and the C-helix rotates slightly, uncoiling its center section, and facilitating binding of the cognate NADPH-cytochrome P450 reductase in the vicinity of helix C. A slight reorientation of helix I caused by interaction with the hydrophobic tail of lanosterol contributes to a significant repositioning of the F-G helices. It changes in the ionic and hydrogen bond interaction between D298 and 299D, at the N-terminal end of helix I, with basic residues (H257, K261, R272) and Y265 on helix G. This modifies the position of helix G so that the F” helix almost completely closes the entry to the substrate channel at the membrane surface. A positional change in the M487 side chain adjacent to helix F” is also involved in substrate entry channel closure. This includes the formation of a water-mediated hydrogen bond network involving M378 and I379, located in a substrate entry channel-lining internal loop, and the hydroxyl group of the bound lanosterol.

The short region linking the F–G helices together with the *β*1/*β*2 loop has considerable importance in membrane-bound CYP450s as they interact with the lipid bilayer surface and contribute to the mouth of a substrate entry channel through which hydrophobic substrates migrate from the membrane to the active site. While structures of recombinant N-terminal truncated eukaryotic cytochrome P450s, which present the enzyme’s catalytic domain, are consistent with this relationship, it can be more directly inferred from biochemical labelling studies, computer simulations, and most importantly, the crystal structures obtained for the full-length fungal CYP51 from *Saccharomyces cerevisiae* (Fig. 13, *A* and *B*) and the more recent crystal structures of *Candida glabrata* and *Candida albicans* CYP51s (507, 508). These molecules include an N-terminal membrane associated *α*-helix and a TM that also interfaces *via* hydrophobic and hydrogen-bonding interactions with a portion of the surface of the catalytic domain. This feature not only provides a membrane anchor but it also orients the catalytic domain so substrates and inhibitors can enter the substrate entry channel from the lipid bilayer. The substrate entry channel and active site have been mapped in numerous structures of *S. cerevisiae* CYP51 complexed with ligands including a range of antifungal azole inhibitors including posaconazole, itraconazole, fluconazole, voriconazole, and VT-1161 plus several azole fungicides used in agriculture (508, 509, 510, 511). These structures identify interactions between these ligands and the heme, specificity-conferring amino acid residues lining the interior cavity of the protein, and with key water molecules. Also visualized were interactions affected by species-specific substitutions that confer inherent resistance to some classes of azole drugs (512) or mutations that confer acquired resistance to some or all classes of azole drugs (513, 514). The structural and phenotypic impacts of these substitutions and mutations are important drivers of efforts to identify novel antifungals that will circumvent these target-based azole-resistant phenotypes (503).

A feature invoked for rational antifungal design is the similarity across phyla of CYP51 structures and the absence of major structural rearrangements in complexes with various inhibitory ligands or structural analogs (503, 515). However, structures obtained for full-length and truncated CYP51s in complex with the short-tailed tetrazole inhibitor VT-1161 and the long-tailed triazole inhibitor posaconazole suggest that the disposition of the mouth of the substrate entry channel required for broad spectrum antifungal activity may be compromised in truncated structures liganded with the short-tailed azole due to structure distorting intersubunit crystal lattice interactions (509). Furthermore, poor substrate binding for both truncated and full-length CYP51 molecules led to conflicting proposals for substrate orientation. The likely orientation of sterol substrates was recently clarified using an I105 F mutant of *Trypanosoma cruzi* CYP51 (516). The mutation converted a fungi-like eburicol-specific CYP51 to a plant-like obtusifoliol-specific enzyme but with substrate occupancy increased to ∼85%. This allowed reliable visualization of this substrate in the binding cavity formed by the B–C loop, helix C, and helix I, with the obtusifoliol hydroxyl group oriented into the substrate access channel. Comparable visualization of the substrate lanosterol was achieved with the human CYP51 D231A H314A mutant (Fig. 13, *B* and *C*) that has the salt bridge involved in proton delivery ablated (517). Furthermore, with productive substrate binding by both the protozoan and human enzyme, a significant reorientation of helix C occurred; the heme propionate-helix C ionic linkage was lost and the freed basic side chain projected outward from the enzyme surface.

Membrane-bound cytochrome P450s operate in a more complex environment than the soluble enzymes and may perform as molecular machines. They interact with a cognate NADPH-cytochrome P450 reductase (CPR) that supplies pairs of electrons, in some instances with cytochrome *b*_*5*_ and, when taking part in metabolic pathways, with multiple downstream enzymes that use the product of the cytochrome P450. For example, an additional channel in the fungal CYP51 enzymes bifurcates from the substrate entry channel (508). This channel has been proposed to mediate product egress and interaction with enzymes immediately downstream in the ergosterol biosynthesis pathway, such as the Erg24 reductase and the demethylating complex of Erg25, Erg26, and Erg27 that are mounted on the Erg28 scaffold protein. Some of these enzyme–enzyme interactions have been mapped in yeast systems using the split-ubiquitin system (518).

A recent study using docking techniques and molecular dynamics has modeled possible interactions between membrane-bound mammalian CPR and membrane-bound CYP1A1 (449). The mimicking of complementary ionic, van der Waals, and hydrophobic interactions between the CPR FMN domain and the residues on the B, C, and L-helices, the J-K loop and the loop structure in the proximal surface near the CYP1A1 heme, plus the inclusion of a hydrogen bond between the FMN phosphate group and the Q139 sidechain in helix C, appeared to enable efficient electron transfer to the heme. Crystallographic and NMR analysis of the bacterial cytochrome P450s, the camphor binding CYP101A, and mycinamicin biosynthetic enzyme MycG, indicate the movement of particular secondary structure elements during substrate binding (450, 451). This finding has been validated by site-directed mutagenesis experiments and used to suggest a generally conserved mechanism for substrate binding and recognition in the P450 superfamily. In CYP51s, accommodation of the substrate in a catalytically competent position is now expected to drive reorientation of helix C and CPR binding, close the substrate entrance channel, and activation of the proton relay machinery *via* F-F”-G arm repositioning and the His-acid salt-bridge opening required for the O–O bond heterolysis (Fig. 13, *B* and *C*). This process has been suggested to prepare the CYP51 catalytic machinery for the three consecutive reaction cycles using compound 1 characteristic of this class of cytochrome P450. It occurs without the substrate release after its first and second monooxygenation reactions, distinguishing it from most other cytochrome P450s (447).

A major challenge in the P450 field is understanding how the membrane-spanning domains impact on the mechanistic basis of eukaryotic cytochrome P450 function. For members of the membrane-bound CYP51 family this will include determining how the protein subunits of this complex molecular machine interact in an ordered fashion and how its sterol substrate is extracted from the membrane and reoriented in order to present appropriately to a series of active sites. This is now feasible through the incorporation of full-length eukaryotic cytochrome P450s, along with their cognate MP partners, into membrane-mimicking environments such as nanodiscs, together with the application of NMR and cryo-EM (452, 453). Using structural knowledge of cytochrome P450 conformational states to generate new classes of antifungal agents not susceptible to side effects caused by drug–drug interactions with drug metabolizing cytochrome P450s in humans is an exciting and much needed endeavor.

### Can structural data provide a mechanistic understanding of the loss-of-function mutations in the cystic fibrosis transmembrane-conductance regulator and guide the development of compensatory therapies?

CF is a well-known genetic disease affecting approximately 70,000 people worldwide (519). It is caused by diverse mutations in the gene coding for the ABC transporter CFTR, which leads to defective biosynthesis or misfolded or dysfunctional CFTR protein. Lack of functional CFTR in epithelial tissues disrupts salt homeostasis, leading to progressive lung disease, exocrine pancreatic insufficiency, and intestinal abnormalities, among many other symptoms. In the lung, CF is characterized by excessive mucus accumulation contributing to obstructive pulmonary disease, which is the main cause of morbidity and mortality. Although still an incurable disease, life expectancy for CF patients has steadily improved to around 40 years thanks to better disease management and treatments that resulted in part from a better understanding of CFTR (520, 521, 522).

Since the discovery of the linkage between CF and mutations in the CFTR gene in 1989 (523, 524, 525), significant progress has been made in mechanistic understanding and therapy design. CFTR is an ABC transporter that shares the two TMDs and two NBDs that hydrolyze ATP characteristics of this superfamily (see How do drugs control our minds? Molecular insights for psychiatric drugs), but includes an additional regulatory (R) domain and an N-terminal lasso motif (526). It is the only known member of the ATP-gated ion channel family, transporting chloride in an ATP-dependent and R domain phosphorylation-dependent manner (361, 527, 528). More than 350 CF-causing CFTR variants have been identified (http://cftr2.org/). Two classes of CFTR-modulator therapies have been designed to compensate for the malfunctioning protein generated by these mutations. These include correctors such as lumacaftor, tezacaftor, and elexacaftor that assist in CFTR protein folding and trafficking to the cell surface, while potentiators such as ivacaftor improve CFTR’s activity (529, 530). Without structural information available, the main discovery and optimization approaches involved intensive high-throughput screening and structure–activity relationship (SAR) campaigns (531, 532).

**Figure 14:**
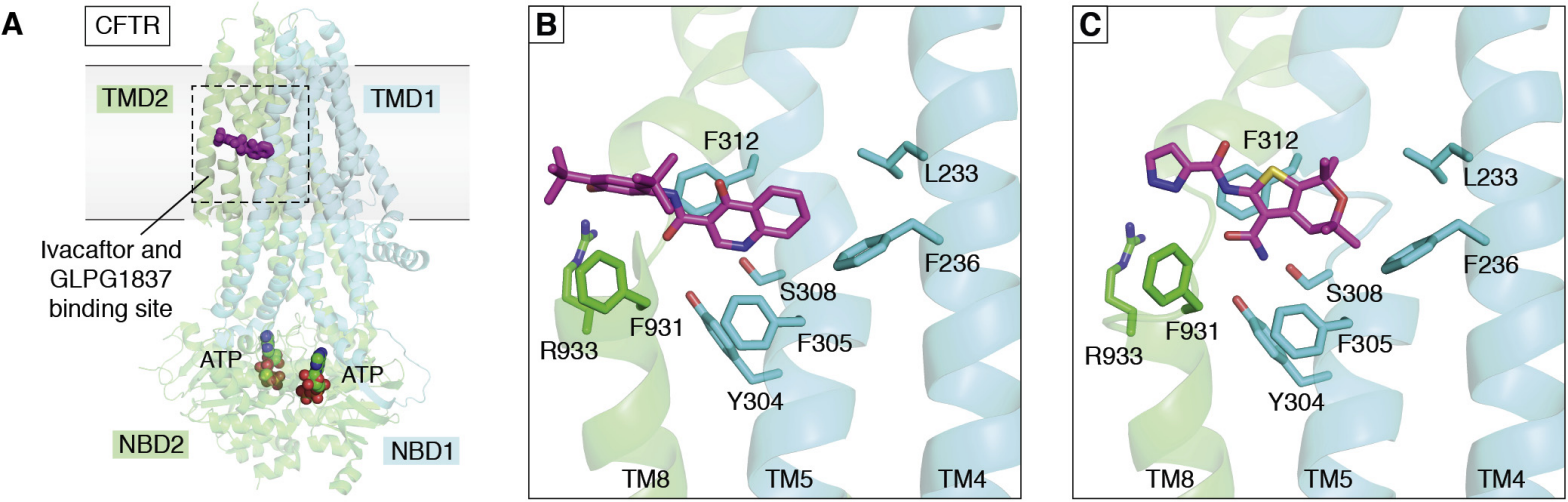
**Structural understanding of CFTR function and therapeutic targeting.***A*, structure of the phosphorylated, ATP-bound hCFTR in complex with ivacaftor (PDB 6O2P). *Ribbon* representation, with TMD1 and NBD1 colored in *cyan*, TMD2 and NBD2 in *green*. The approximate location of the lipid bilayer is shown in *gray*. Ivacaftor is shown in *magenta*, in *stick* representation. ATP-Mg^2+^ molecules are shown in *sphere* representation (carbon in *green*, nitrogen in *blue*, oxygen in *red*, Mg^2+^ in *yellow*). *B*, close-up image of main interactions between hCFTR and Ivacaftor (PDB 6O2P). A selected number of interacting residues are shown in *stick* representation, carbons are colored in *cyan* (TMD1) or *green* (TMD2), nitrogen is *blue*, oxygen is *red*. *C*, close-up image of main interactions between hCFTR and GLPG1837 (PDB 6O1V). Main interacting residues are shown in *stick* representation, carbons are colored in *cyan* (TMD1) or *green* (TMD2), nitrogen is *blue*, oxygen is *red*, sulfur is *yellow*.

Recent structural efforts by Chen and colleagues are changing that scenario, by solving CFTR structures in the dephosphorylated ATP-free form (379, 381), phosphorylated ATP-bound form (380, 533), and phosphorylated ATP-bound form in complex with potentiators (534). Many CF-causing mutations could be mapped into these structures, and their negative effect rationalized as affecting the pore construction, protein folding, the ATPase site, or the NBD/TMD interface. The cryo-EM structure of the phosphorylated ATP-bound human CFTR (hCFTR) E1371Q mutant in complex with potentiators ivacaftor (an FDA-approved drug developed by Vertex Pharmaceuticals) and GLPG1837 (an investigational drug developed by Galapagos) shed light on how these molecules improve hCFTR activity in CF patients (Fig. 14*A*) (534). Ivacaftor-bound E1371Q, phosphorylated and ATP-bound, exhibits the same conformation as the ivacaftor-free structure. Interestingly, ivacaftor binds CFTR at the protein–lipid interface in a clef formed by TM helices 4, 5 and 8. As shown in previous structures, TM8 rotates upon phosphorylation and ATP binding, and ivacaftor appears to stabilize this rotation. Ivacaftor might thus function by favoring the open conformation of the pore. Residues L233, F236. Y304, F305, S308, F312, F931, and R933 are identified as key in the CFTR–ivacaftor interaction (Fig. 14*B*) and correlate with previously published SAR studies that led to ivacaftor’s development (531). The elucidation of GLPG1837-bound E1371Q, phosphorylated and ATP-bound structure (Fig. 14*C*) showed that although chemically dissimilar to ivacaftor, its interaction with CFTR largely overlaps ivacaftor’s binding site. Both ivacaftor and GLPG1837 were discovered by extensive high-throughput screening, followed by further optimizations through SAR (531, 532). The availability of these potentiator-bound structures, and future corrector-bound structures, could represent a new avenue for the development of more efficient CF treatments.

Other structures of ABC transporters have contributed to therapy. It is noteworthy that the breast cancer resistance protein (BCRP or ABCG2) and P-glycoprotein efflux a number of drugs, affecting their pharmacokinetics and bioavailability and mediate multidrug resistance. Their structures were elucidated in different conformations, in the presence and absence of drugs, paving the way for the development of a newer generation of drugs that can inhibit or evade multidrug resistance associated with these transporters (386, 387, 388, 389, 390, 391, 392).

### How does understanding of a glucose transporter selectivity and conformational changes inform the development of GLUT1-specific inhibitors?

Glucose is an essential source of energy used by tumor cells to produce ATP, maintain the reduction–oxidation balance, and generate biomass (535). Glucose transporter GLUT1 is overexpressed in many types of cancers, including the brain, colon, kidney, lung, ovary, and prostate, and represents a promising target for therapy (536). However, there are 14 GLUT transporters in humans, and due to their high sequence homology, many GLUT inhibitors lack isoform specificity (537, 538, 539, 540, 541). The development of GLUT1-specific inhibitors would thus benefit from the understanding of its atomic structure and the identification of key residues that could be targeted for selective inhibition.

Initial structural and mechanistic understanding of glucose transport by GLUTs was provided by studies focusing on bacterial homologs (see How do drugs control our minds? Molecular insights for psychiatric drugs). The first human GLUT1 (hGLUT1) structure was determined by Deng *et al.* (403). It contained mutations N45T, which removed a glycosylation site, and E329Q, which blocked the transporter in an inward-facing conformation. Interestingly, a molecule of *β*-nonyl glucoside, the detergent used during purification, was present in the inward-open cavity, with the D-glucopyranoside headgroup bound to the C-terminal domain near the center of the membrane, overlapping with the glucose-binding site observed in the bacterial homolog XylE structure in complex with glucose (434). The structure provided a molecular basis for interpreting mutations linked to disease. These inactivating variants could be clustered within the substrate-binding site, at the interface between the TMDs and the intracellular helix (ICH) domain, and throughout the lining of the transport path.

**Figure 15:**
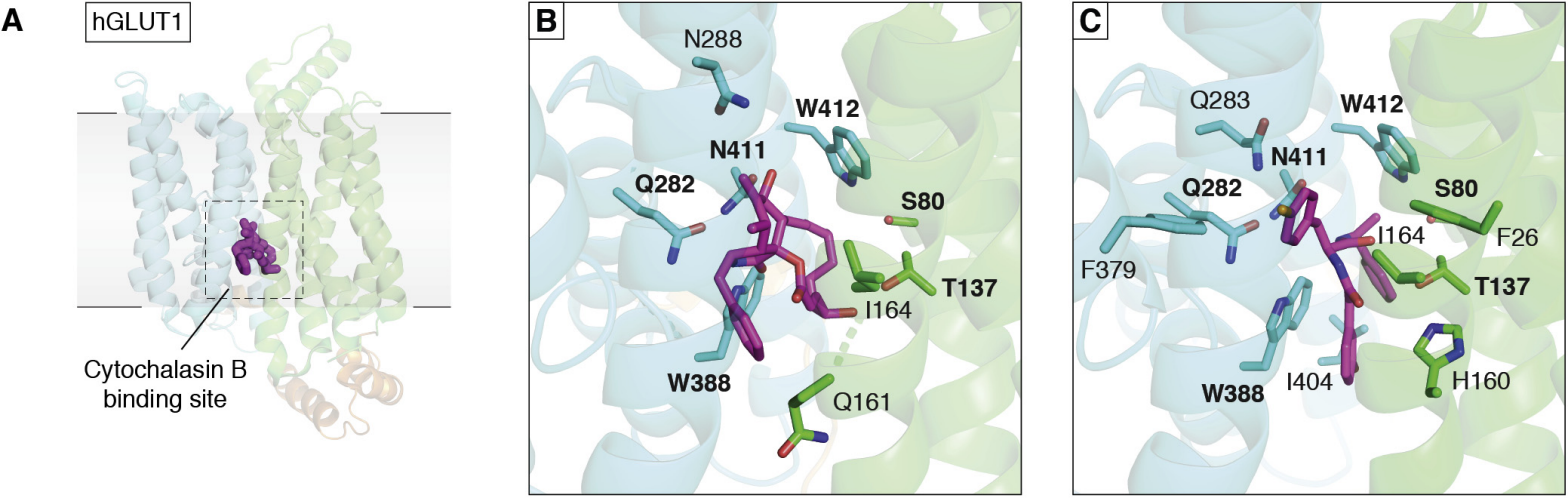
**Structural understanding of the function and transport inhibition of human GLUTs.***A*, structure of hGLUT1 bound to cytochalasin B (PDB 5EQI). hGLUT1 is in *cartoon* representation, with its N-terminal domain colored *green*, ICH *orange*, and the C-terminal domain *cyan*. Cytochalasin B is shown in *stick* representation, colored in *magenta*. The approximate location of the lipid bilayer is shown in *gray*. *B*, close-up image of main interactions between hGLUT1 and cytochalasin B (PDB 5EQI). Main hGLUT1 interacting residues and ligand are shown in *stick* representation, carbons are colored in *green* (N-terminal domain), *cyan* (C-terminal), or *magenta* (ligand), nitrogen is *blue*, oxygen is *red*. Residue labels in *bold* highlight common interacting residues with cytochalasin B, GLUT1-i1, and GLUT-i2. *C*, close-up image of main interactions between hGLUT1 and GLUT1-i1 (PDB 5EQG). Same labeling and coloring scheme as in (*B*), with the addition of fluorine (*orange*).

Kapoor *et al.* (405) reported three structures of wild-type hGLUT1 cocrystallized with drug leads, the cell-permeable mycotoxin cytochalasin B, and two new inhibitors identified by high-throughput screening, GLUT-i1 and GLUT-i2. Despite different chemical structures, the three inhibitors bind to the proposed glucose-binding site in the central cavity, with hGLUT1 residues S80, T137, Q282, W388, N411, and W412 interacting with all three ligands (Fig. 15). The IC_50_ values of cytochalasin B, GLUT-i1, and GLUT-i2 bound to GLUT2-4 highlighted differences in specificity. When combining the crystal structure of GLUT1 bound to these compounds with the corresponding structure-derived molecular models for hGLUT2-4 bound to them, comparison of interacting residues and potential differences could inform the development of future, more-specific compounds.

The structural information generated represents a practical basis for the development of more specific GLUT1 inhibitors. While the previous development of GLUT inhibitors was based on high-throughput screening and SAR-based optimizations (536), activity-based and *in silico* screening campaigns to identify specific GLUT inhibitors relying on structural information are starting to bear fruit (542, 543, 544). The structure of the main sugar transporter *Pf*HT1 from the malaria parasite was determined recently by two groups (545, 546). In one of the studies, the high-resolution structure was used to allow rational design of more potent and selective inhibitors of *Pf*HT1 that could be used to treat malaria. With an increasing number of GLUT structures being determined, one can expect a new generation of potent and specific GLUT inhibitors developed with similar structural-based strategies to treat diverse diseases. Public access of structures through PDB is no doubt a pillar for such endeavors.

### How do drugs control our minds? Molecular insights for psychiatric drugs

The majority of signal transduction events in our brain are mediated by small molecules known as neurotransmitters. Neurotransmitters in the cytosol must be packaged into synaptic vesicles by vesicular neurotransmitter transporters before they can be released from the presynaptic axon by exocytosis upon action potential stimulation (547, 548, 549). The released neurotransmitter then elicits their effects by binding to two kinds of membrane receptors on the postsynaptic dendrite: ionotropic ligand-gated ion channels (LGICs) for fast response or metabotropic GPCRs for slow effects (550, 551). To ensure proper synaptic transmission, it is equally important to terminate the signal promptly by rapid removal of neurotransmitters from the synapses after their release. This is often achieved by high-affinity neurotransmitter transporters located on the plasma membrane of presynaptic termini and in the surrounding glial cells (552, 553) or by diffusion and hydrolysis of the neurotransmitter (554).

Use of chemical substances to change mood and behavior dates back several thousands of years with well-known examples including coca (cocaine), coffee (caffeine), alcohol, and marijuana (cannabis). Modern research on these compounds as psychiatric drugs began after World War II with advances in chemical synthesis and animal testing. In parallel, critical advancements in neuroscience and molecular biology allowed the identification of the target proteins for these drugs and understanding of their effects. It is not a surprise that most of these compounds target MPs located at the synapse. In fact, almost all MPs that bind neurotransmitters are intensively studied as drug targets. Particularly, LGICs and neurotransmitter sodium symporters (NSSs) are amongst the top ten families of all FDA-approved drugs, accounting for 7.9% and 2.7% respectively (555). On the other hand, the recent epidemic of drug abuse spotlights a lack of advancement in this area. In fact, most currently used psychiatric drugs were developed in the mid-to-late 20th century. Their use/misuse is associated with side effects, and they are criticized for contributing to addiction. This highlights the need to develop more specific drugs with less side effects and that are less likely to be addictive. The availability of structures of these MPs in complex with their endogenous ligands and/or drugs in the PDB is the basis for such efforts. Research on psychiatric drugs over the past 50 years is a good example of how structures of MPs have helped to elucidate the mechanisms of action of drugs.

**Figure 16:**
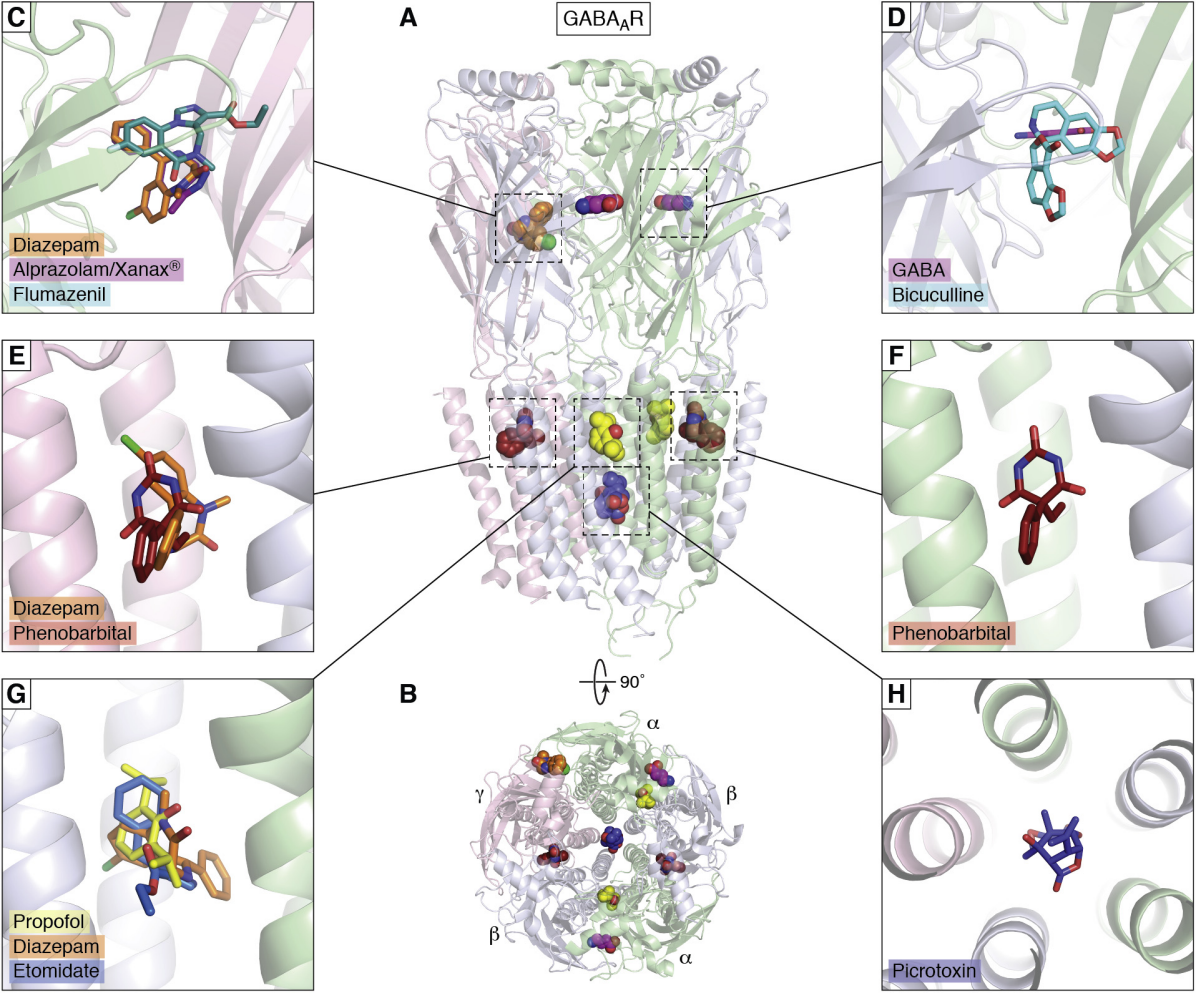
**Structural basis of psychiatric drugs targeting GABA**_**A**_**R.***A* and *B*, overall structure of GABA_A_R with one representative ligand at each distinctive binding site shown in *sphere* representation. *C*–*H*, composite structure of GABA_A_R with ligands binding at different sites (PDB 6HUO, 6HUP, 6HUK, 6HUJ, 6HUG, 6I53, 6D6U, 6X3X, 6X3U, 6X3Z, 6X40, 6X3S, 6X3W, 6X3V, 6X3T).

LGICs located on the postsynaptic cells are the major effectors of neurotransmitters. They underlie the rapid phasic synaptic transmission. The receptor for the major inhibitory neurotransmitter GABA is a Cl^−^ channel that hyperpolarizes the postsynaptic cell and reduces the firing probability of the receptor neuron when activated (550). As a result, ligands of GABA_A_R are highly sought-after as psychiatric drugs. GABA_A_R belongs to the Cys-loop receptor family together with the receptors for glycine and acetylcholine, all of which have had their structure and pharmacology intensively investigated (41, 75, 550, 556, 557, 558, 559, 560, 561, 562, 563, 564, 565, 566, 567, 568, 569). The structure of the Cys-loop receptors forms a pentameric ion channel made of five similar but nonidentical subunits (Fig. 16, *A* and *B*). Each heteropentameric channel is typically assembled with 2 *α*, 2 *β* and 1 *γ* (or *δ*) subunits. There are 19 genes encoding for the *α*, *β*, and *γ* subunits of the GABA_A_R in humans that are differentially expressed in different regions of the brain. The assembly of different subunits of GABA_A_R results in complex heterogeneity in their structures and is the major determinant of their differing functions in distinct regions of the brain and their complex pharmacological profile (570, 571). Several recent structures of GABA_A_R in complex with various ligands have provided unprecedented insight into the molecular mechanism of some commonly used psychiatric drugs (556, 564, 566, 567, 568). For instance, both Valium (benzodiazepine) and Xanax (alprazolam) bind to GABA_A_R at the benzodiazepine site between subunit *α* and *γ* on the extracellular ligand-binding domain (Fig. 16*C*) while the endogenous ligand GABA as well as bicuculline binds at the interface between the *α* and *β* subunits (Fig. 16*D*). Structures of GABA_A_R revealed that Benzodiazepines potentiate the effect of GABA by improving the connectivity between subunits allosterically while bicuculline competes with GABA at the same binding site to prevent the channel from opening. On the other hand, Flumazenil, which is used clinically to treat benzodiazepine overdose, competes with benzodiazepines at the same binding site (Fig. 16*C*). The neurotoxin picrotoxin, which is used as stimulant and convulsant, directly blocks the ion-conducting pore (Fig. 16*H*). The structures also revealed a previously unknown second binding site for the benzodiazepine at the transmembrane region (Fig. 16*E*) as well as several binding sites for anesthesia agents in the transmembrane region (Fig. 16, *F* and *G*). This information highlights new possibilities for drug interaction. The structures of GABA_A_R bound with substrate and drug ligands are major step forward toward better psychiatric drugs.

Another way of modulating synaptic transmission is by regulating the concentration of neurotransmitters at the synaptic cleft. In fact, the most popular marketed antidepressants, such as Prozac (Fluoxetine), Zoloft (Sertraline), Celexa (Citalopram), all use this strategy. The selective serotonin reuptake inhibitors (SSRIs) are a group of drugs targeting serotonin transporters on the plasma membrane of the presynaptic neurons (572). As deficiency of serotonin and dopamine (DA) is implicated in depressive disorders, increasing their availability at the synapse through blocking their reuptake is commonly used to treat depression. The dopamine pathway has been targeted for the treatment of depression since the early 1950s with the use of monoamine oxidase inhibitors (MAOIs) and tricyclic antidepressants (TCAs) (572). However, they have undesirable adverse effects and a high potential for toxicity. With the identification and cloning of the transporters for serotonin (SERT), dopamine (DAT), and noradrenaline (NET), SSRIs are designed to specifically inhibit SERT but with minimal inhibition of NET, which was the source of side effect of antihistamine used earlier. Major chemical synthesis efforts together with *in vitro/in vivo* screening and testing from major pharmaceutical companies successfully identified several SSRIs that were much more selective and with less adverse effects compared with TCAs. Since 1987, an increasing number of SSRIs have been marketed and used to treat depression till the current day and hailed as a great success for rational drug design. However, the secret of success remains somewhat mysterious, as no one really understood how these drugs were able to interact selectively with SERT, but not the very similar DAT and NET, at the molecular level. The availability of structures of these transporters in complex with substrates and drug ligands is starting to provide the answer.

**Figure 17:**
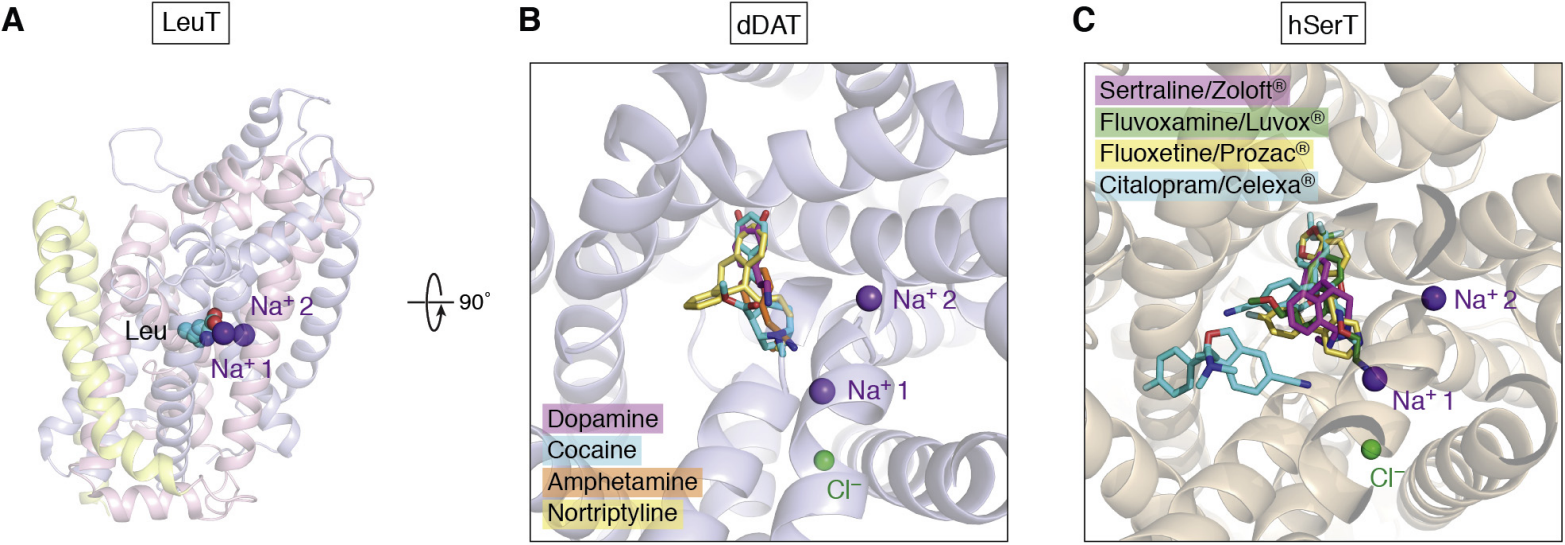
**Structural basis of antidepressants targeting SLC6 family NSSs.***A*, structure of LeuT (PDB 2A65) defines the common structure of the SLC6 family transporters. LeuT is composed of 12 TMs. TM1-10 forms the signature “5 + 5” inverted repeat with TM1-5 (*light blue*) and TM6-10 (*pink*) forming the core domain repeating each other with a pseudo twofold symmetry along the substrate binding site. TM11 and TM 12 (*yellow*) are located at the peripheral of the core domain. The substrate leucine is shown as *cyan sphere* while the two sodiums are shown in *purple*. *B*, ligand-binding site structure of dDAT in complex with substrate dopamine as well as other psychostimulants (PDB 4M48, 4XP6, 4XP9, 4XP1, 4XP4, 4XP5). *C*, ligand-binding site structure of hSERT in complex with common antidepressants (PDB 6AWO, 5I73, 5I6X, 6AWP). Na^+^ is shown in *purple* while Cl^−^ is shown in *green*.

SERT, DAT, NET all belongs to the SLC6 family of NSSs. They are high-affinity transporters for biogenic amines (dopamine, serotonin, and noradrenaline) driven by the sodium gradient across the plasma membrane. Following intense efforts, the structure of a bacterial homolog of SLC6 family transporters, a leucine transporter from *Aquifex aeolicus*, LeuT, was first determined in 2005 (60). LeuT revealed the “5 + 5” inverted repeat structural fold for the first time (Fig. 17*A*), which is highly conserved throughout evolution. The structure of LeuT also identified two highly conserved Na^+^-binding sites and elucidated the substrate transport mechanism. More importantly, the structure of LeuT provided a blueprint for understanding the substrate specificity of SLC6 transporters, which are central for the selectivity and efficacy of the SSRI antidepressants. In one approach, the substrate-binding site of LeuT, which binds to leucine naturally, was engineered to harbor residues that would bind to the biogenic amines. Structures of the chimeric transporter LeuBAT in complex with four classes of antidepressants (SSRIs, serotonin norepinephrine reuptake inhibitor, TCA, and Mazindol) were determined, and this provided the first glimpses of how these drugs interact with NSS transporters (573). The structures of *Drosophila* DAT in complex with endogenous substrate dopamine, as well as cocaine, amphetamines, and TCA antidepressants were subsequently determined in 2013 and 2015 (574, 575) (Fig. 17*B*). Finally, the structure of the human SERT was determined recently in complex with several TCAs and SSRIs by both X-ray crystallography and cryo-EM (576, 577, 578, 579) (Fig. 17*C*). These structures clearly illustrate that the majority of antidepressants targeting NSSs function through competing with the endogenous substrates at the central binding site. However, a second allosteric binding site at the entrance to the central binding site was observed in several structures, suggesting additional factors that may influence the selectivity. These structures also further elucidated the transport mechanism of eukaryotic NSSs, which are also regulated by Cl^−^, K^+^, and membrane lipids. These structures provide a much better understanding of how antidepressants act upon NSSs. Their availability in the PDB provides an invaluable resource for structure-based drug discovery and development that will surely lead to better antidepressants.

### How can membrane proteins be engineered to advance biological discovery?

Neuroscientists have dreamed for decades of controlling specific groups of neurons in free-living animals to interrogate functions of neuronal circuits. Optogenetics enables exactly that, with the help of a family of light-sensitive algal membrane channels called Channelrhodopsins (ChRs). Protein engineering is enabling fine-tuning of the properties of ChRs to allow an ever-growing range of applications (https://web.stanford.edu/group/dlab/optogenetics/index.html). This topic has been reviewed previously by the founders of the field, Deisseroth and Hegemann (580, 581). Here we briefly illustrate the contribution of PDB in the exciting endeavor to understand the human brain and behavior.

**Figure 18:**
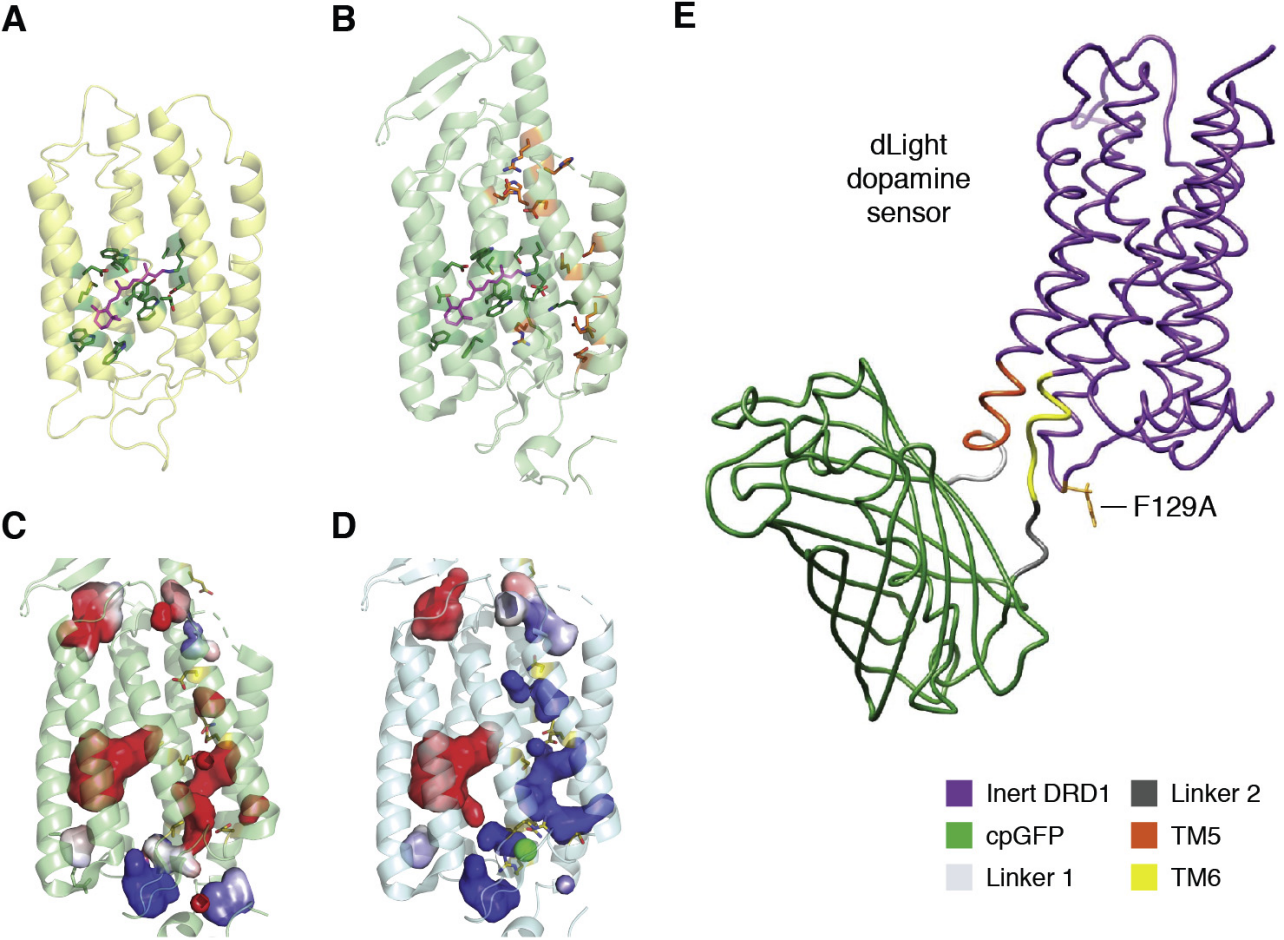
**Protein engineering applications based on membrane proteins.***A*, structure of the bacteriorhodopsin (PDB 1AP9). *B*, structure of the C1C2 ChR (PDB 3UG9). In both (*A*) and (*B*), the retinal is shown as *magenta sticks* while residues forming the retinal binding site are shown as *dark green sticks*. Residues along the ion-conducting pathway of ChRs are shown as *orange sticks* on the C1C2 structure in (*B*). Refer to Ref. (580) for more details. *C*, electrostatics of the vestibule of C1C2 ChR. *D*, electrostatics of the vestibule of the designed anion channel iC^++^ (PDB 6CSN). *E*, design of the dLight dopamine sensor (used with permission (602)).

Although naturally existing ChRs have been used in pioneering optogenetic studies since 2005 (582), it was really structure-based protein engineering that fueled the tremendous developments that allowed it to become a powerful and versatile tool for neuroscience. ChRs are seven TM integral MPs that use retinal as chromophore and function as cation channels. Fortunately, the structure of their remote cousin, bacteriorhodopsin, was among the first MPs to be determined. Bacteriorhodopsin and ChRs share the same 7-TM structure fold with a similar binding site for the chromophore retinal (Fig. 18, *A* and *B*). With hundreds of structures already determined and detailed biophysical characterization available, bacteriorhodopsin allowed models of ChRs to be built. These helped in optimizing the optical properties and resulted in several useful modified ChRs. For instance, the ChETA variant was obtained by mutating E123 of ChR2 and allows fast-firing at 200 Hz or more (583). Modification of TM3-TM4 interaction at the CD pair (C128-D156 in ChR2) resulted in the step-function opsins, SFOs, which enable bistable excitation photon-spike logic (584, 585, 586). A diverse family of red-shifted C1V1 ChRs were created by modification of the ChR from alga *Volvox carteri* (587, 588, 589, 590). The crystal structure of a chimera between ChR1 and ChR2, C1C2, determined in 2012 (591, 592) (Fig. 18*B*) further clarified the location and structure of the light-activated pore and made even more designer ChRs possible. In particular, the structure revealed that the internal vestibules of the cation conducting ChRs are lined with negatively charged residues (Fig. 18*C*). Guided by the structure, mutagenesis of residues lining the vestibule reversed the charge. This resulted in a highly Cl^−^ selective ChRs (iC^++^) (Fig. 18*D*) (593, 594) now widely used to elicit inhibitory stimulation. Optogenetic is a fast-growing field with applications requiring ChRs with a wide range of properties. As illustrated here, the availability of the structures of the natural and designed ChRs will continue to be a major driving force for further advancements.

Genetically encoded sensors are widely used in biophysical and cell biological investigations. It is not an overstatement that fluorescent proteins, such as the green fluorescent proteins (GFP), enabled modern cell biology. Structure-guided protein engineering that combines a fluorescent protein with another functional group has allowed various physiological processes to be monitored and investigated by fluorescent methods in cell cultures and in animals. For instance, the calcium sensor GCaMP was created by fusing of the GFP with the calcium-binding protein calmodulin and an M13 peptide from myosin light chain kinase (595). Similarly, the glutamate sensor iGluSnFR (intensity-based glutamate-sensing fluorescent reporter) was obtained by fusion of a circularly permutated enhanced green fluorescent protein (cpEGFP) with a bacterial glutamate-binding protein (596, 597). Both GCaMP and iGluSnFR, as well as their variants, are now widely used to study calcium signaling and glutamate neurotransmission. Due to the difficulties with MPs, applications of genetically encoded MPs lagged behind those of soluble proteins by a few decades. In recent years, however, with the exponential growth in the available structures of MPs, applications of MPs as genetically encoded sensors are gradually emerging.

Dopamine is an important neuromodulator of complicated behaviors such as motivation, rewards, learning, and motor control. There is thus a large demand for monitoring its concentration in cells and in animals. However, unlike other neurotransmitters, such as glutamate and GABA, dopamine exclusively activates the metabotropic GPCRs. Without eliciting any direct electrical response, it is hard to monitor dopamine by using electrophysiological techniques. Fortunately, the structures of three out of the four dopamine receptors (all members of the GPCR family) were determined recently (598, 599, 600). The structures allowed two groups to independently design fluorescence sensors for dopamine based on the dopamine receptors. Tan’s group designed several dopamine fluorescent sensors (dLight) by replacing the third intracellular loop of human dopamine receptor D1, D2, and D4 with the cpGFP module from the GCaMP6 (Fig. 18*E*). The first generation of dLight sensors have already been successfully used in cell cultures, slice preparations, and free-moving mice. Similarly, Li’s group obtained the GPCR-activation-based dopamine sensors, GRAB_DA_, by inserting the cpEGFP into the intracellular loop 3 of the D2 dopamine receptor. This construct enabled successful measurement of dopamine concentration in mice, fishes, and flies (601).

With an increasing number of MP structures determined in recent years, there is no doubt that more applications in addition to those illustrated here will emerge. The free availability of structures to all protein engineers is critical knowledge that will allow this field to thrive.

## Future perspective

The Coronavirus Disease 2019 (COVID-19) pandemic dominated world attention in 2020 (603, 604). The main routes to eliminate this infectious disease currently focus on two MPs: the viral spike protein (605, 606), and the membrane-bound form of the Angiotensin Converting Enzyme 2 (ACE2) on the lung epithelial cell (607). Vaccines target the viral spike protein to activate an immune response. Peptides attempt to target the ACE2 site without altering its essential human function (608). The primary preventative focus now includes mRNA-based and virus-based vaccines that encode the Spike protein and other membrane proteins that then excite the immune response ahead of encounter with the virus. Vaccines encoding the Spike protein mRNA use lipid-based micelles or viral vectors for entry into human cells. The lessons learned in 2020 provide a template to combat pandemics. Can we now develop general strategies for COVID-19, and for inevitable future infectious agents, by preventing the early-stage interactions required for pathogenesis? Prevention of the spike protein–ACE2 interaction, a feature that was swiftly determined at the structural level, has the potential to provide stop-gap therapy until a much-needed cure can be discovered and implemented. For example, monoclonal antibodies or VH domains could block critical entry interactions. Structural science has produced several exciting templates for this important direction.

The newest exciting tool in this endeavor is a protein folding program, *AlphaFold 2*, created by Google’s DeepMind group and showing revolutionary accuracy in predicting protein structure directly from sequence in a blinded competition (the 14th Critical Assessment of Structural Prediction competition, CASP14) (609). The program, utilizing “*Artificial Intelligence*” trained on the high-quality structures available in the PDB, follows closely on the heels of the cryo-EM revolution and heralds a future with rapid access to more precise MP structures, potentially solving the bottleneck in structure-based drug design. The structures of MPs are, so far, less accurately predicted, but the Tidow group reports that after 2 years of unsuccessful work on solving the crystal structure of the MP FoxB, they succeeded using DeepMind’s prediction in a few hours by molecular replacement (610). Undoubtedly the program will become even more precise and valuable for MPs as new structures are solved by the powerful technological advances discussed above.

Traditionally, many long-standing and emerging diseases have been accorded low priority by the pharmaceutical industry due to the limited commercial value of potential therapies. We might imagine that MP-based strategies that target key first steps in the pathogenesis of such diseases could become a focus of the World Health Organization. Given the experience of 2020, there should be good reason to hope that scientific funding organizations will have the insight and preparedness to accelerate funding for approaches that investigate the key membrane-associated mechanisms defining the pathogenicity of each newly found infectious agent, be it viral or microbial, as soon as its danger is recognized. There are some critical limitations in readily obtaining the structures of MPs. These include “pulling out” the full complement of molecular machines from their wild-type living organism in action, expressing functional MP at the levels required for structural studies, addressing the underrepresentation of the full-length structures of low-membrane pass proteins, and the ongoing challenge of obtaining relevant structures that reveal the key conformational changes during reaction or transport cycles. Advances in X-ray laser-based structure determination for small crystals and cryo-EM already provide valuable technological additions to the current armamentarium of biologists interested in MP structure and function (611, 612).

Few proteins work alone! Proteomic mapping has expanded the prospects for understanding MP function in greater detail, particularly in contexts in which complexes of proteins come together to regulate and coordinate multiple regulated functions. Mass spectrometry is key to identifying the proteins that associate with each other over time and circumstance (603, 604). One very exciting approach is to ask such questions in a normal cell without the overexpression of proteins. This can be achieved by genetically adding an affinity tag then “pulling it down” with the proteins actively associated with it (613). Cryo-EM is well suited to determining structures of larger complexes of proteins, including those at low endogenous levels (Most extreme translocation: Effector protein and virulence factor secretion in pathogens). This process will dramatically expand horizons over coming years, with affinity purification of large complexes of proteins revealing the mechanisms of such assemblies. Furthermore, cryo-EM can visualize multiple structural states at different stages in their reaction cycles and even model multiple and anharmonically coupled states. In principle the distribution of these states provides snapshots of the system in action and can be used to estimate the free energy difference between states of similar probability. Such approaches promise numerous new structures and insights that will close the gap between the functions of proteins acting as individuals and their roles within the complexes in which they function, in different states across time within a normal cellular environment.

Determining the relationships between structure and function of MPs relies on communication, deposition, and dissemination of structural coordinates. Here we highlight a small selection of discoveries from a plethora that have stemmed from these endeavors. We provide a glimpse into how MP structure determination has been enabled by parallel technological developments, how these structures inform about biological function and mechanism, and how MP structures supply foundations for diverse fields including protein engineering, the simulation, and further exploration of biological functions and processes associated with membranes, as well as drug discovery and design. The PDB provides an ever-growing treasury of MP structures—an everlasting, powerful, and enriching product of global efforts.

## Conflict of interest

The authors declare that they have no conflicts of interest with the contents of this article.
